# Supplementary material for: Spatial transcriptomics reveals altered communities and drivers of aberrant epithelia and pro-fibrotic fibroblasts in interstitial lung diseases
Source: Cell Genom. 2026 Jan 22;6(3):101066. doi: 10.1016/j.xgen.2025.101066 (PMC12985369; doi:10.1016/j.xgen.2025.101066)
Supplement: Document S1. Figures S1–S9 [file mmc1.pdf]

Cell Genomics, Volume *6*

Supplemental information

Spatial transcriptomics reveals altered communities and drivers of aberrant epithelia and pro-fibrotic fibroblasts in interstitial lung diseases

Alok Jaiswal, Tristan Kooistra, Vladislav Pokatayev, Hélder N. Bastos, Rita F. Santos, Tresa R. Sarraf, Åsa Segerstolpe, Crystal Lin, Liat Amir-Zilberstein, Shaina Twardus, Kevin Shannon, Shane P. Murphy, Rachel Knipe, Ingo K. Ganzleben, Katharine E. Black, Toni M. Delorey, Daniel B. Graham, Yin P. Hung, Lida P. Hariri, Jacques Deguine, Agostinho Carvalho, Benjamin D. Medoff, and Ramnik J. Xavier

Supplemental Information

Spatial transcriptomics reveals altered communities and drivers of aberrant epithelia and pro-fibrotic fibroblasts in interstitial lung diseases

Alok Jaiswal,[1,2] Tristan Kooistra,[3,4] Vladislav Pokatayev,[1,2,5] Hélder N. Bastos,[6,7,8] Rita F. Santos,[7,9] Tresa R. Sarraf,[3,4] Asa Segerstolpe,[10] Crystal Lin,[10] Liat Amir-Zilberstein,[10] Shaina Twardus,[11] Kevin Shannon,[11] Shane P. Murphy,[10] Rachel Knipe,[3,4] Ingo K. Ganzleben,[3,4] Katharine E. Black,[3,4] Toni M. Delorey,[10] Daniel B. Graham,[1,2,5,10], Yin P. Hung,[12] Lida P. Hariri,[3,12] Jacques Deguine,[1,10] Agostinho Carvalho,[13,14] Benjamin D. Medoff,[3,4] Ramnik J. Xavier[1,2,5,10,15,16,17*]

[1]Broad Institute of MIT and Harvard, Cambridge, MA, 02142, USA
[2]Department of Molecular Biology, Massachusetts General Hospital, Boston, MA, 02114, USA
[3]Division of Pulmonary and Critical Care Medicine, Massachusetts General Hospital and Harvard Medical School, Boston, MA, 02114, USA
[4]Center for Immunology and Inflammatory Diseases, Massachusetts General Hospital and Harvard Medical School, Boston, MA, 02114, USA
[5]Center for Computational and Integrative Biology, Massachusetts General Hospital and Harvard Medical School, Boston, MA, 02114, USA
[6]Department of Pneumology, Hospital de São João, 4200-319 Porto, Portugal
[7]i3S – Instituto de Investigação e Inovação em Saúde, Universidade do Porto, 4200-135 Porto, Portugal
[8]Faculty of Medicine/RISE-Health, University of Porto, 4200-319 Porto, Portugal
[9]School of Health Sciences – Polytechnic of Porto, 4200-072 Porto, Portugal
[10]Klarman Cell Observatory, Broad Institute of MIT and Harvard, Cambridge, MA, 02142, USA
[11]Division of Gastroenterology, Massachusetts General Hospital and Harvard Medical School, Boston, MA, 02114, USA
[12]Department of Pathology, Massachusetts General Hospital and Harvard Medical School, Boston, MA, 02114, USA
[13]Life and Health Sciences Research Institute (ICVS), School of Medicine, University of Minho, 4710-057 Braga, Portugal
[14]ICVS/3B's - PT Government Associate Laboratory, 4710-057 Braga/4806-909 Guimarães, Portugal
[15]Center for the Study of Inflammatory Bowel Disease, Massachusetts General Hospital, Boston, MA, 02114, USA
[16]The Gene Lay Institute of Immunology and Inflammation, Brigham and Women's Hospital, Massachusetts General Hospital, Harvard Medical School, Boston, MA, 02115, USA
[17]Lead contact
*Correspondence: xavier@molbio.mgh.harvard.edu

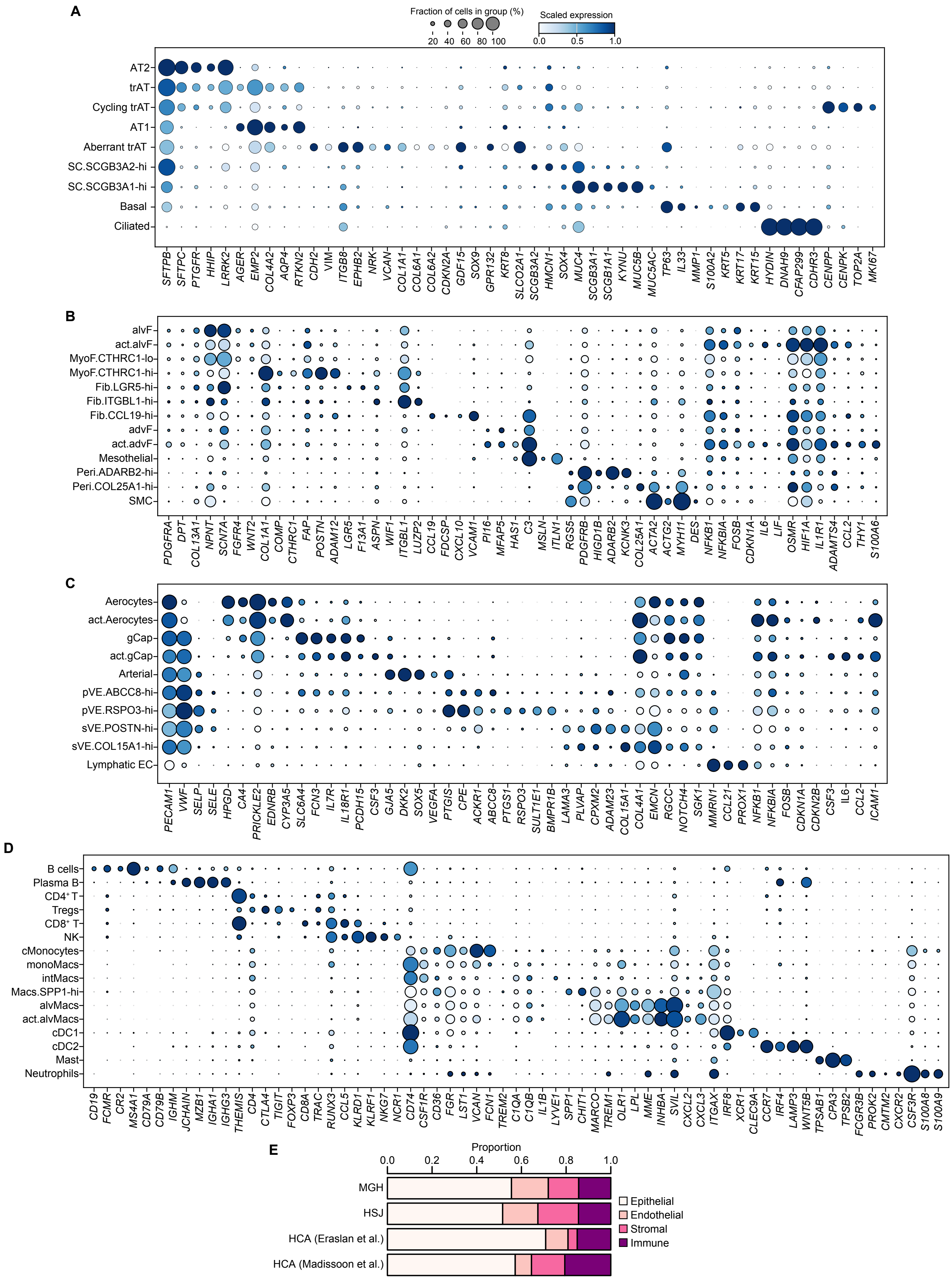
A
Fraction of cells in group (%)
20 40 60 80 100
Scaled expression
0.0 0.5 1.0
AT2
trAT
Cycling trAT
AT1
Aberrant trAT
SC.SCGB3A2-hi
SC.SCGB3A1-hi
Basal
Ciliated
SFTPB SFTPC PTGFR HHIP LRRK2 AGER EMP2 COL4A2 AQP4 RTKN2 CDH2 VIM ITGB8 EPHB2 NRK VCAN COL1A1 COL6A1 COL6A2 CDKN2A GDF15 SOX9 GPR132 KRT8 SLCO2A1 SCGB3A2 HMCN1 SOX4 MUC4 SCGB3A1 SCGB1A1 KYNU MUC5B MUC5AC TP63 IL33 MMP1 S100A2 KRT5 KRT17 KRT15 HYDIN DNAH9 CFAP299 CDHR3 CENPP CENPK TOP2A MKI67
B
alvF
act.alvF
MyoF.CTHRC1-lo
MyoF.CTHRC1-hi
Fib.LGR5-hi
Fib.ITGBL1-hi
Fib.CCL19-hi
advF
act.advF
Mesothelial
Peri.ADARB2-hi
Peri.COL25A1-hi
SMC
PDGFRA DPT COL13A1 NPNT SCN7A FGFR4 WNT2 COL1A1 COMP CTHRC1 FAP POSTN ADAM12 LGR5 F13A1 ASPN WIF1 ITGBL1 LUZP2 CCL19 FDCSP CXCL10 VCAM1 PI16 MFAP5 HAS1 C3 MSLN ITLN1 RGS5 PDGFRB HIGD1B ADARB2 KCNK3 COL25A1 ACTA2 ACTG2 MYH11 DES NFKB1 NFKBIA FOSB CDKN1A IL6 LIF OSMR HIF1A IL1R1 ADAMTS4 CCL2 THY1 S100A6
C
Aerocytes
act.Aerocytes
gCap
act.gCap
Arterial
pVE.ABCC8-hi
pVE.RSPO3-hi
sVE.POSTN-hi
sVE.COL15A1-hi
Lymphatic EC
PECAM1 VWF SELP SELE HPGD CA4 PRICKLE2 EDNRB CYP3A5 SLC6A4 FCN3 IL7R IL18R1 PCDH15 CSF3 GJA5 DKK2 SOX5 VEGFA PTGIS CPE ACKR1 ABCC8 PTGS1 RSPO3 SULT1E1 BMPR1B LAMA3 PLVAP CPXM2 ADAM23 COL15A1 COL4A1 EMCN RGCC NOTCH4 SGK1 MMRN1 CCL21 PROX1 NFKB1 NFKBIA FOSB CDKN1A CDKN2B CSF3 IL6 CCL2 ICAM1
D
B cells
Plasma B
CD4+ T
Tregs
CD8+ T
NK
cMonocytes
monoMacs
intMacs
Macs.SPP1-hi
alvMacs
act.alvMacs
cDC1
cDC2
Mast
Neutrophils
CD19 FCMR CR2 MS4A1 CD79A CD79B IGHM JCHAIN MZB1 IGHA1 IGHG3 THEMIS CD4 CTLA4 TIGIT FOXP3 CD8A TRAC RUNX3 CCL5 KLRD1 KLRF1 NKG7 NCR1 CD74 CSF1R CD36 FGR LST1 VCAN FCN1 TREM2 C1QA C1QB IL1B LYVE1 SPP1 CHIT1 MARCO TREM1 OLR1 LPL MME INHBA SVIL CXCL2 CXCL3 ITGAX IRF8 XCR1 CLEC9A CCR7 IRF4 LAMP3 WNT5B TPSAB1 CPA3 TPSB2 FCGR3B PROK2 CMTM2 CXCR2 CSF3R S100A8 S100A9
E
Proportion
0.0 0.2 0.4 0.6 0.8 1.0
MGH
HSJ
HCA (Eraslan et al.)
HCA (Madissoon et al.)
Epithelial
Endothelial
Stromal
Immune

Figure S1: Annotation of snRNA-seq atlas subsets, related to Figure 1.
A-D. Cell type-specific markers used to annotate snRNA-seq clusters among epithelial (A), stromal (B), endothelial (C), and immune (D) compartments. Dot size indicates fractions of cells expressing the gene and color indicates scaled expression values.
E. Proportions of cells in the epithelial, endothelial, stromal, and immune compartments in each cohort used to generate the snRNA-seq atlas.

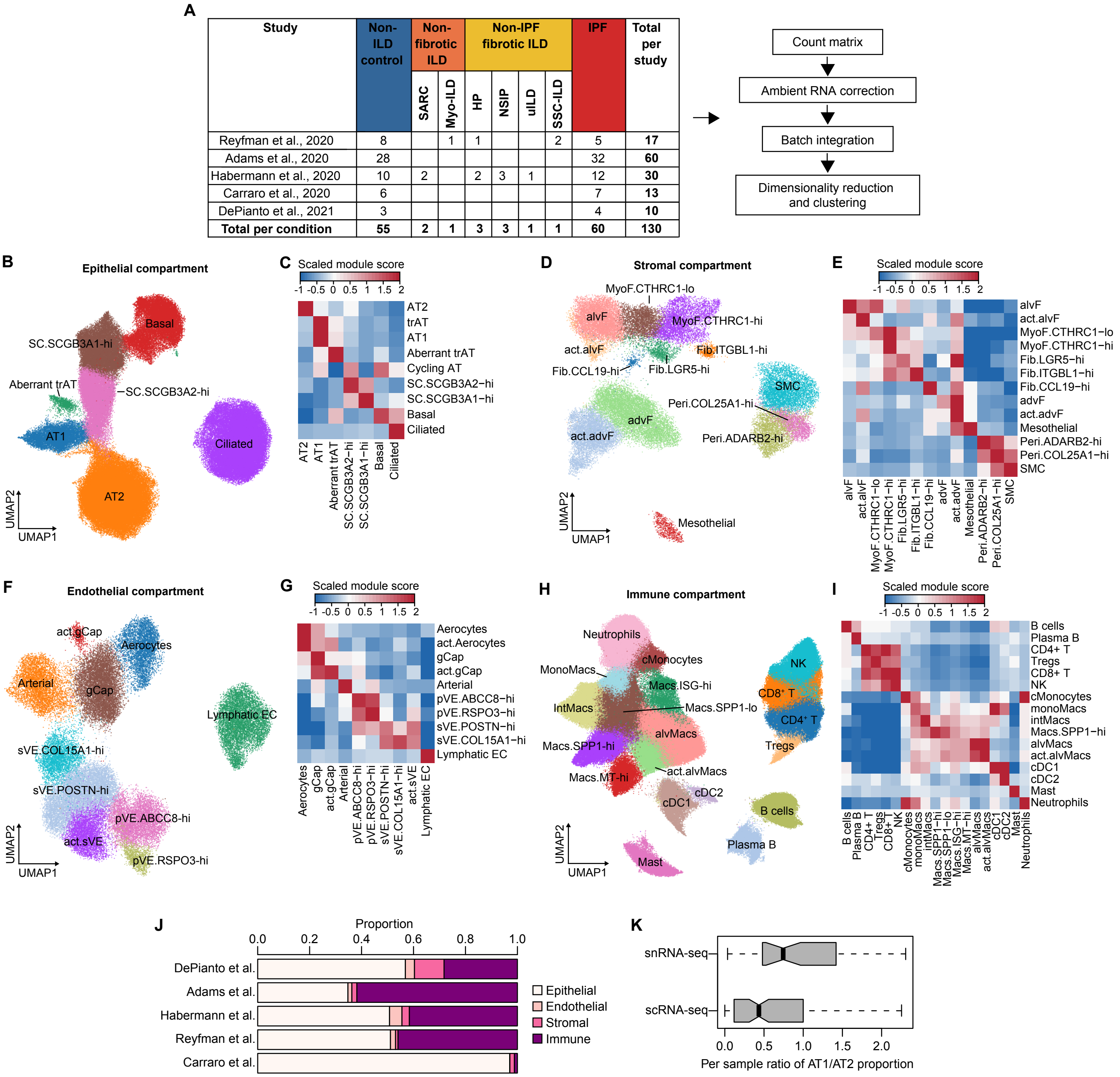

Study	Non-ILD control	Non-fibrotic ILD		Non-IPF fibrotic ILD				IPF	Total per study
		SARC	Myo-ILD	HP	NSIP	uILD	SSC-ILD		
Reyfman et al., 2020	8		1	1			2	5	**17**
Adams et al., 2020	28							32	**60**
Habermann et al., 2020	10	2		2	3	1		12	**30**
Carraro et al., 2020	6							7	**13**
DePianto et al., 2021	3							4	**10**
**Total per condition**	**55**	**2**	**1**	**3**	**3**	**1**	**1**	**60**	**130**

Figure S2: Assembly of a scRNA-seq atlas of ILD from published datasets, related to Figure 1.
A. Left: Sample sizes, by disease status, of published scRNA-seq datasets included in a meta-analysis for comparison with the snRNA-seq atlas. Right: Meta-analysis pipeline overview.
B-H. UMAP representation of the epithelial (B), stromal (D), endothelial (F) and immune (H) compartments of the joint scRNA-seq atlas, alongside the heatmap of module scores derived from the snRNA-seq atlas populations (y-axis) and calculated across the scRNA-seq subsets (x-axis) for the same epithelial (C), stromal (E), endothelial (G) and immune (I) compartments. **J.** Compartment proportions in the scRNA-seq data, separated by study.
K. Box plot of AT1/AT2 cell proportion per patient, showing that snRNA-seq has better recovery of AT1 cells compared to scRNA-seq. Bar plots represent the quartiles and whiskers represent the 10-90% range of values.

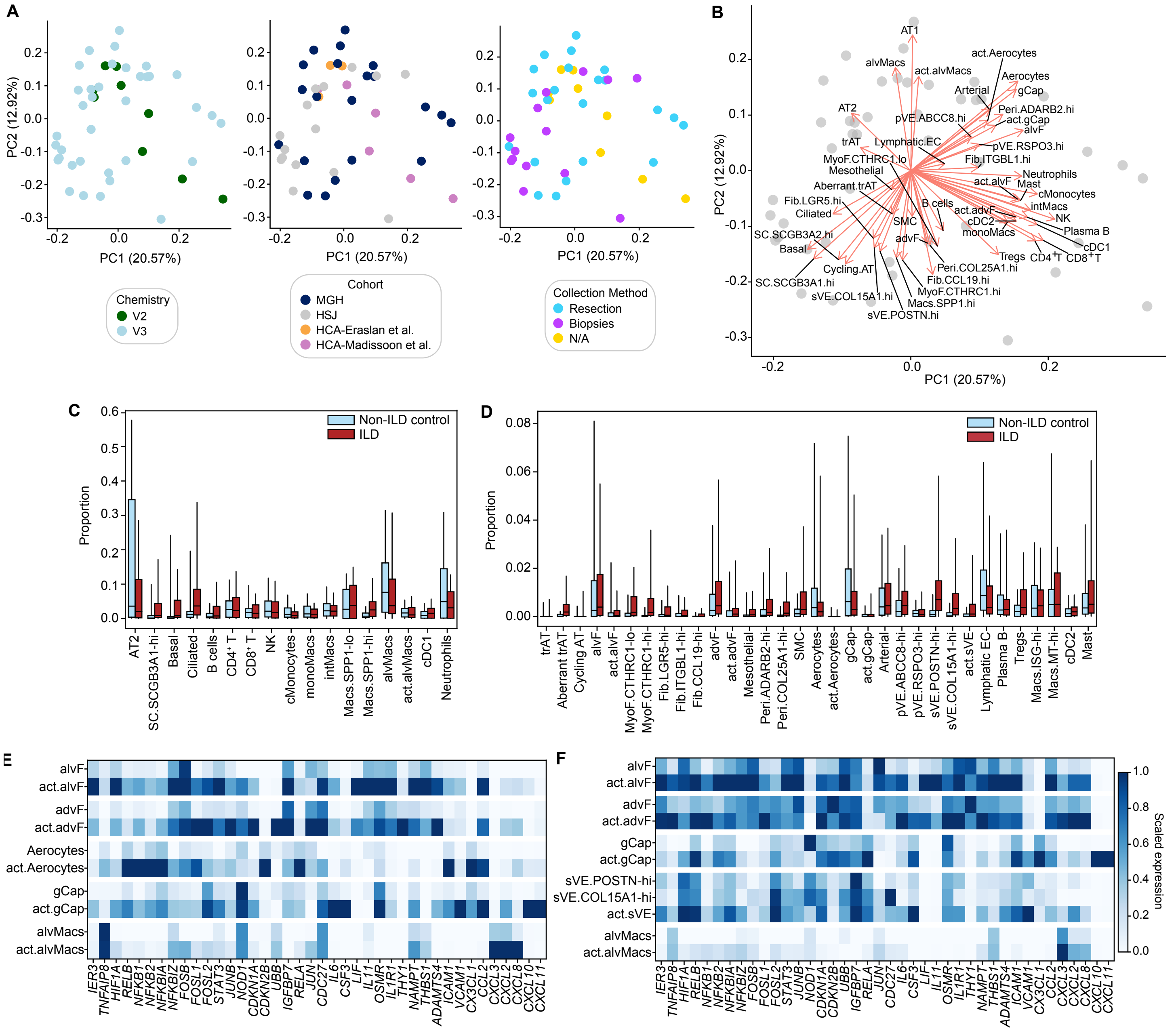
A
PC2 (12.92%)
PC1 (20.57%)
Chemistry
V2
V3
Cohort
MGH
HSJ
HCA-Eraslan et al.
HCA-Madissoon et al.
Collection Method
Resection
Biopsies
N/A
B
PC2 (12.92%)
PC1 (20.57%)
AT1
alvMacs
act.alvMacs
act.Aerocytes
Aerocytes
Arterial
gCap
AT2
pVE.ABCC8.hi
Peri.ADARB2.hi
act.gCap
alvF
trAT
Lymphatic.EC
pVE.RSPO3.hi
MyoF.CTHRC1.lo
Fib.ITGBL1.hi
Mesothelial
Neutrophils
act.alvF
Mast
Aberrant.trAT
cMonocytes
Fib.LGR5.hi
B cells
intMacs
Ciliated
act.advF
NK
SMC
cDC2
Plasma B
SC.SCGB3A2.hi
monoMacs
advF
cDC1
Basal
Tregs
CD4+T
CD8+T
Cycling.AT
Peri.COL25A1.hi
Fib.CCL19.hi
SC.SCGB3A1.hi
MyoF.CTHRC1.hi
sVE.COL15A1.hi
Macs.SPP1.hi
sVE.POSTN.hi
C
Proportion
Non-ILD control
ILD
AT2
SC.SCGB3A1-hi
Basal
Ciliated
B cells
CD4+ T
CD8+ T
NK
cMonocytes
monoMacs
intMacs
Macs.SPP1-lo
Macs.SPP1-hi
alvMacs
act.alvMacs
cDC1
Neutrophils
D
Proportion
Non-ILD control
ILD
trAT
Aberrant trAT
Cycling AT
alvF
act.alvF
MyoF.CTHRC1-lo
MyoF.CTHRC1-hi
Fib.LGR5-hi
Fib.ITGBL1-hi
Fib.CCL19-hi
advF
act.advF
Mesothelial
Peri.ADARB2-hi
Peri.COL25A1-hi
SMC
Aerocytes
act.Aerocytes
gCap
act.gCap
Arterial
pVE.ABCC8-hi
pVE.RSPO3-hi
sVE.POSTN-hi
sVE.COL15A1-hi
act.sVE
Lymphatic EC
Plasma B
Tregs
Macs.ISG-hi
Macs.MT-hi
cDC2
Mast
E
alvF
act.alvF
advF
act.advF
Aerocytes
act.Aerocytes
gCap
act.gCap
alvMacs
act.alvMacs
IER3
TNFAIP8
HIF1A
RELB
NFKB1
NFKB2
NFKBIA
NFKBIZ
FOSB
FOSL1
FOSL2
STAT3
JUNB
NOD1
CDKN1A
CDKN2B
UBB
IGFBP7
RELA
JUN
CDC27
IL6
CSF3
LIF
IL11
OSMR
IL1R1
THY1
NAMPT
THBS1
ADAMTS4
ICAM1
VCAM1
CX3CL1
CCL2
CXCL3
CXCL2
CXCL8
CXCL10
CXCL11
F
alvF
act.alvF
advF
act.advF
gCap
act.gCap
sVE.POSTN-hi
sVE.COL15A1-hi
act.sVE
alvMacs
act.alvMacs
Scaled expression

Figure S3: Compositional analyses of the snRNA-seq atlas, related to Figure 1.

A. Principal component analysis of annotated cell type composition colored by 10x chemistry, cohort, and tissue collection method (left to right).

B. Cell type loading weights on principal components. Arrow lengths are proportional to weights

C-D. Proportion changes of all cell types stratified by disease condition. Bar plots represent the quartiles and whiskers represent the 10-90% range of values.

E-F. Heatmaps of expression of selected genes specific to activated subsets and their respective basal cell states, in the (**E**) snRNA-seq and (**F**) scRNA-seq atlases. Genes that were differentially enriched in at least one activated subset were plotted. Differential expression analysis was performed using the Wilcoxon test in each broader cell type and genes with adjusted P-value < 0.05 were considered to be statistically significant.

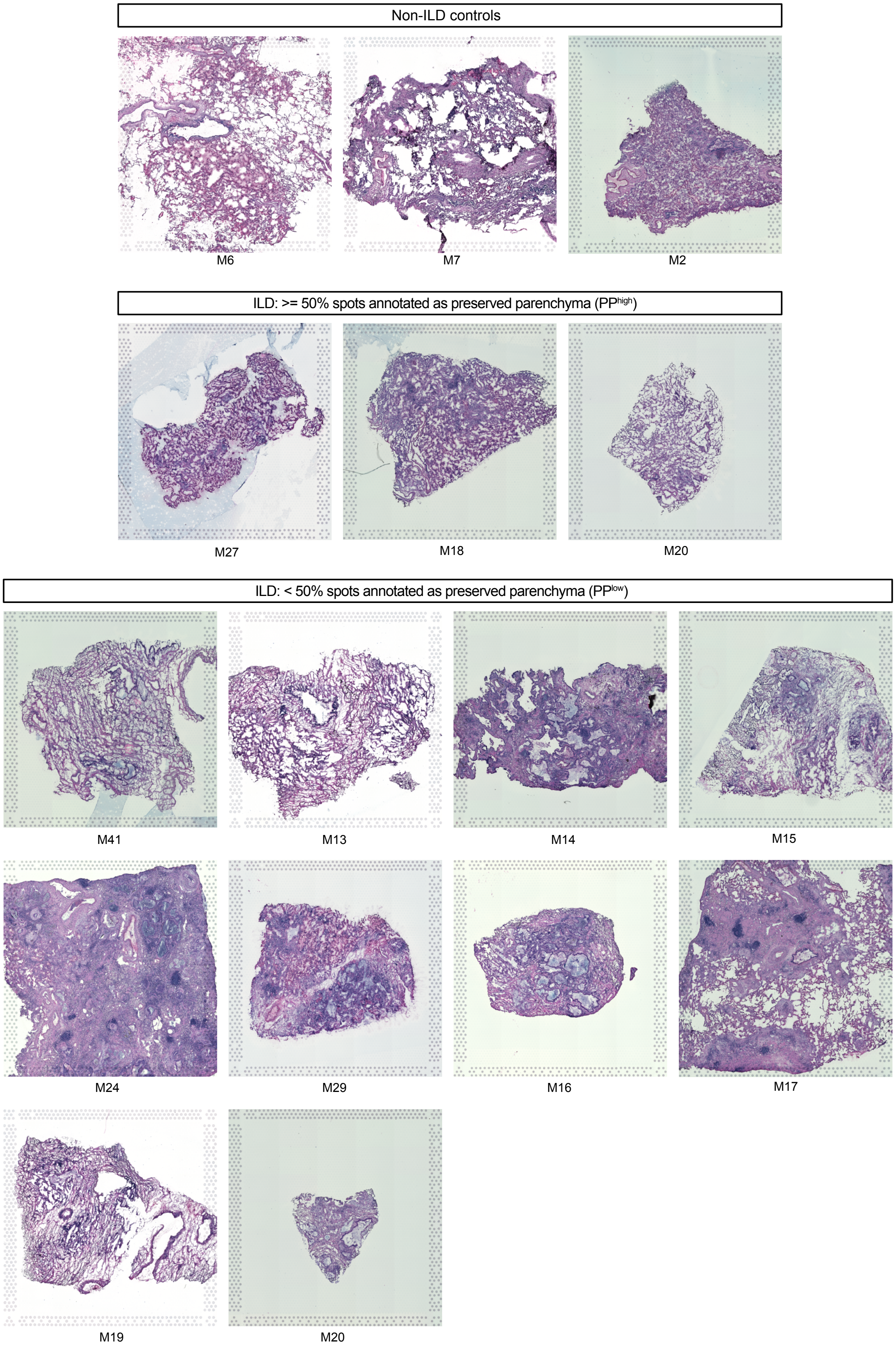
Non-ILD controls
M6
M7
M2
ILD: >= 50% spots annotated as preserved parenchyma (PP^{high})
M27
M18
M20
ILD: < 50% spots annotated as preserved parenchyma (PP^{low})
M41
M13
M14
M15
M24
M29
M16
M17
M19
M20

Figure S4: Histological images of tissues analyzed by spatial transcriptomics, related to Figure 2. H&E stains of tissues profiled by 10x Visium. Each ILD tissue block was inspected and categorized as ILD-PP^{high} (with >= 50% of spots annotated as preserved parenchyma) or ILD-PP^{low} (with < 50% of spots annotated as preserved parenchyma).

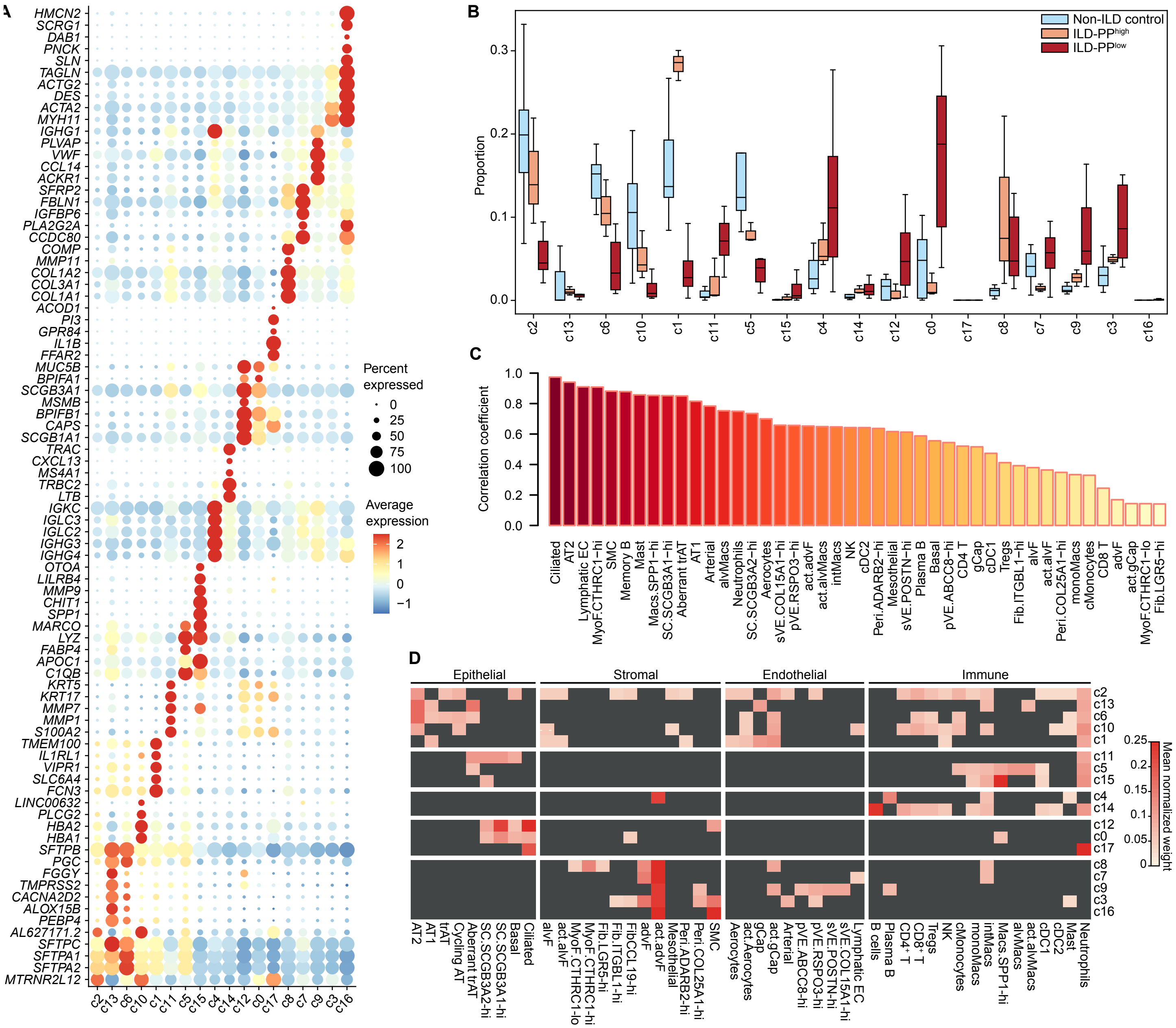
A
B
C
D
Percent expressed
Average expression
Proportion
Non-ILD control
ILD-PPhigh
ILD-PPlow
Correlation coefficient
Epithelial
Stromal
Endothelial
Immune
Mean normalized weight

Figure S5: Annotation of cellular communities detected by spatial transcriptomics, related to Figure 2.

A. Dot plot of expression for the top five genes specific to each cellular community identified in Figure 2B. Dot size represents the percentage of spots with at least 1 count of the gene, and color represents scaled expression values.

B. Proportion of all communities present in individual samples, grouped by disease condition and tissue histology. Bar plots represent the quartiles and whiskers represent the 10-90% range of values for individual samples.

C. Pearson correlation coefficients of RCTD-derived normalized cell type weights at each spot using either the scRNA-seq atlas or the snRNA-seq atlas as the reference.

D. Mean RCTD-estimated normalized weights of each cell type and cellular community using the snRNA-seq reference atlas. Cell type weights were statistically compared for each cellular community group to the weights of the rest using the one-sided Student's t-test. Legend color scale represents the mean normalized weight in the respective category for statistically significant (Benjamini-Hochberg adjusted P-value < 0.05) comparisons; insignificant comparisons are colored dark grey.

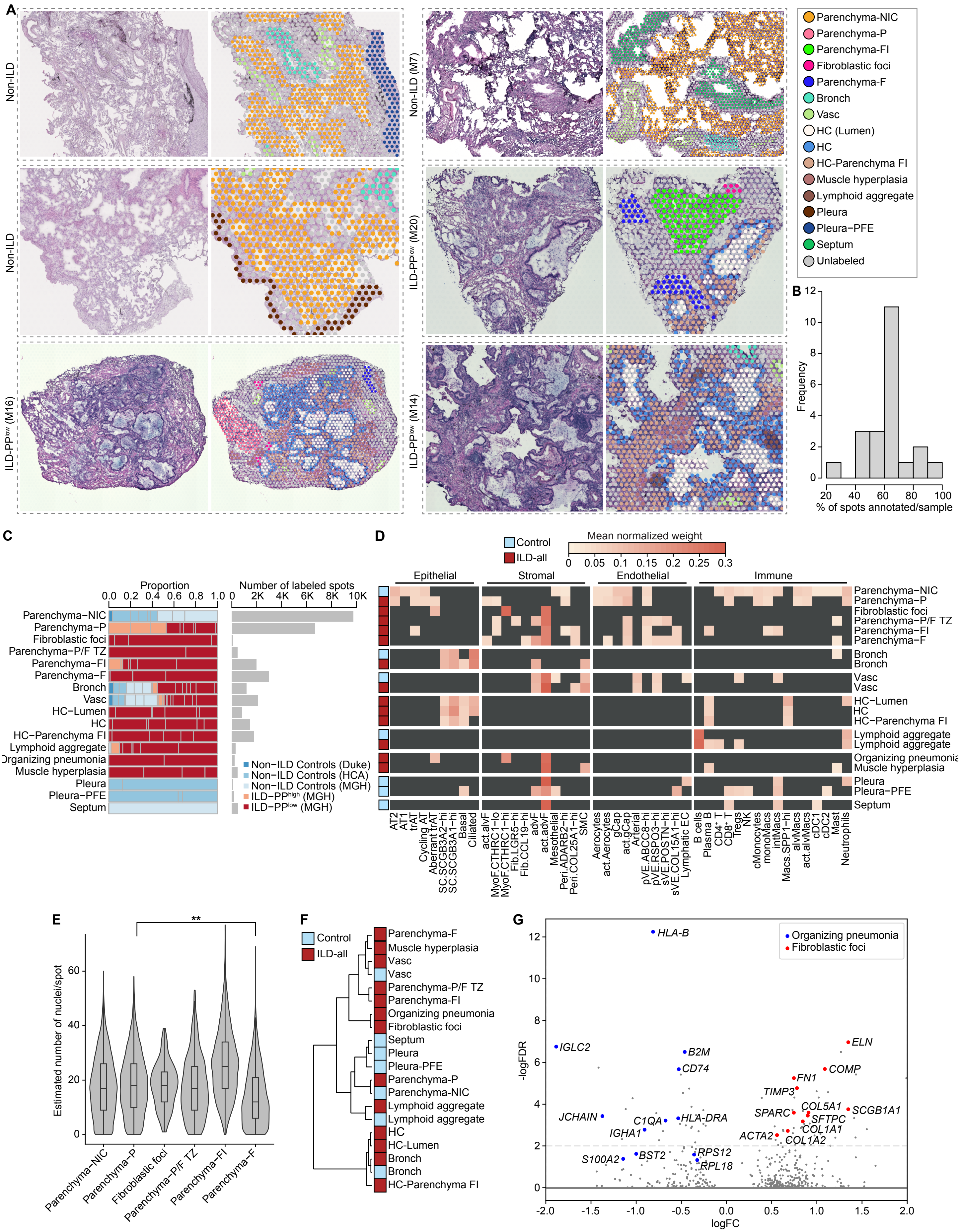

A
Non-ILD
Non-ILD
ILD-PP^low (M16)
Non-ILD (M7)
ILD-PP^low (M20)
ILD-PP^low (M14)
Parenchyma-NIC
Parenchyma-P
Parenchyma-FI
Fibroblastic foci
Parenchyma-F
Bronch
Vasc
HC (Lumen)
HC
HC-Parenchyma FI
Muscle hyperplasia
Lymphoid aggregate
Pleura
Pleura−PFE
Septum
Unlabeled
B
Frequency
% of spots annotated/sample
C
Proportion
Number of labeled spots
Parenchyma−NIC
Parenchyma−P
Fibroblastic foci
Parenchyma−P/F TZ
Parenchyma−FI
Parenchyma−F
Bronch
Vasc
HC−Lumen
HC
HC−Parenchyma FI
Lymphoid aggregate
Organizing pneumonia
Muscle hyperplasia
Pleura
Pleura−PFE
Septum
Non−ILD Controls (Duke)
Non−ILD Controls (HCA)
Non−ILD Controls (MGH)
ILD−PP^high (MGH)
ILD−PP^low (MGH)
D
Control
ILD-all
Mean normalized weight
Epithelial
Stromal
Endothelial
Immune
AT2
AT1
trAT
Cycling AT
Aberrant trAT
SC.SCGB3A2−hi
SC.SCGB3A1−hi
Basal
Ciliated
act.alvF
MyoF.CTHRC1−lo
MyoF.CTHRC1−hi
Fib.LGR5−hi
Fib.CCL19−hi
advF
act.advF
Mesothelial
Peri.ADARB2−hi
Peri.COL25A1−hi
SMC
Aerocytes
act.Aerocytes
gCap
act.gCap
Arterial
pVE.ABCC8−hi
pVE.RSPO3−hi
sVE.POSTN−hi
sVE.COL15A1−hi
Lymphatic EC
B cells
Plasma B
CD4+ T
CD8+ T
Tregs
NK
cMonocytes
monoMacs
intMacs
Macs.SPP1−hi
alvMacs
act.alvMacs
cDC1
cDC2
Mast
Neutrophils
E
Estimated number of nuclei/spot
**
Parenchyma-NIC
Parenchyma-P
Fibroblastic foci
Parenchyma-P/F TZ
Parenchyma-FI
Parenchyma-F
F
Control
ILD-all
Parenchyma-F
Muscle hyperplasia
Vasc
Vasc
Parenchyma-P/F TZ
Parenchyma-FI
Organizing pneumonia
Fibroblastic foci
Septum
Pleura
Pleura-PFE
Parenchyma-P
Parenchyma-NIC
Lymphoid aggregate
Lymphoid aggregate
HC
HC-Lumen
Bronch
Bronch
HC-Parenchyma FI
G
Organizing pneumonia
Fibroblastic foci
-logFDR
logFC
HLA-B
IGLC2
B2M
CD74
JCHAIN
C1QA
HLA-DRA
IGHA1
S100A2
BST2
RPS12
RPL18
ELN
COMP
FN1
TIMP3
SPARC
COL5A1
SCGB1A1
SFTPC
COL1A1
ACTA2
COL1A2

Figure S6: Analysis of disease-associated cellular communities, related to Figure 2.

A. Schema of identification of anatomical and histopathological features in representative H&E images for each sample.

B. Histogram of the number of samples, binned by fraction of annotated spots.

C. Left: Normalized proportion of sample level histopathological features colored by diseased status and cohort. Right: Number of annotated spots for each histopathological feature in the total dataset.

D. Mean RCTD-estimated normalized weights of each cell type and histopathological structure using the snRNA-seq reference atlas. Cell type weights were statistically compared for each group vs. the rest using the one-sided Student’s t-test. Legend color scale represents the mean normalized weight in the respective category for statistically significant (Benjamini-Hochberg adjusted P-value < 0.05) comparisons; insignificant comparisons are colored dark gray.

E. Violin plot of estimated number of nuclei per spot in the annotated histopathological regions pertaining to the distal parenchyma. Nuclei segmentation was performed using the watershed algorithm. Nuclei counts per spot were statistically compared between Parenchyma-P and Parenchyma-F using the Wilcoxon test; **P < 0.001.

F. Dendrogram of hierarchical clustering of aggregate transcriptomic profiles of annotated histopathological features separated by patient groups. Dendrogram analysis was performed on the top 35 principal components.

G. Volcano plot showing differentially expressed genes between organizing pneumonia and fibroblastic foci. Wilcoxon test was performed and genes with adjusted P-value < 0.05 were considered statistically significant. Selected genes are highlighted.

For panels A, C-F: NIC - non-ILD control; P - preserved zone; F - fibrotic zone; TZ - transitional zone; FI - fibrotic inflamed zone; Bronch - bronchiole; Vasc - vasculature; HC - honeycomb cyst.

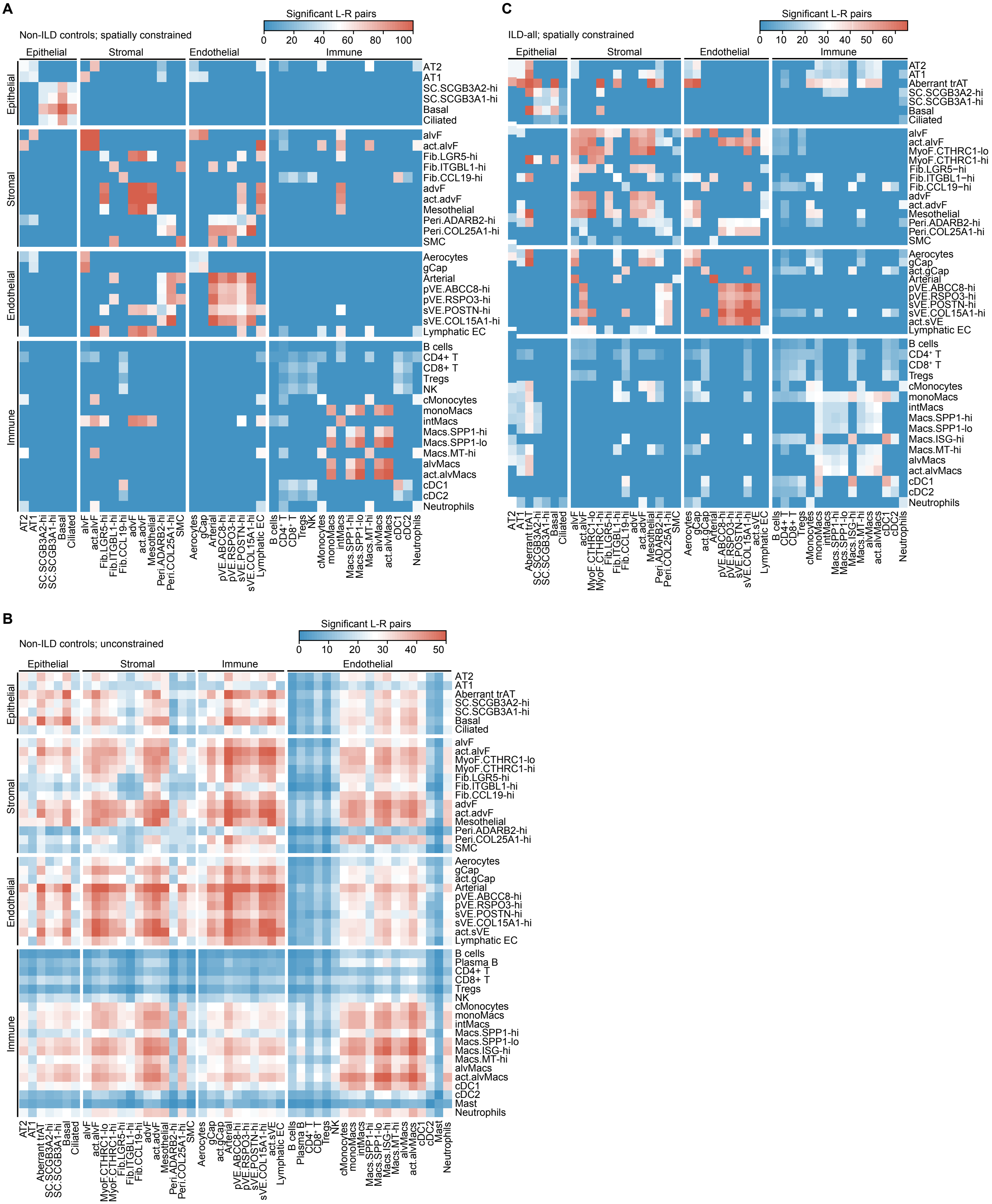
A
Non-ILD controls; spatially constrained
Significant L-R pairs
0 20 40 60 80 100
Epithelial
Stromal
Endothelial
Immune
AT2
AT1
SC.SCGB3A2-hi
SC.SCGB3A1-hi
Basal
Ciliated
alvF
act.alvF
Fib.LGR5-hi
Fib.ITGBL1-hi
Fib.CCL19-hi
advF
act.advF
Mesothelial
Peri.ADARB2-hi
Peri.COL25A1-hi
SMC
Aerocytes
gCap
Arterial
pVE.ABCC8-hi
pVE.RSPO3-hi
sVE.POSTN-hi
sVE.COL15A1-hi
Lymphatic EC
B cells
CD4+ T
CD8+ T
Tregs
NK
cMonocytes
monoMacs
intMacs
Macs.SPP1-hi
Macs.SPP1-lo
Macs.MT-hi
alvMacs
act.alvMacs
cDC1
cDC2
Neutrophils
B
Non-ILD controls; unconstrained
Significant L-R pairs
0 10 20 30 40 50
Epithelial
Stromal
Immune
Endothelial
AT2
AT1
Aberrant trAT
SC.SCGB3A2-hi
SC.SCGB3A1-hi
Basal
Ciliated
alvF
act.alvF
MyoF.CTHRC1-lo
MyoF.CTHRC1-hi
Fib.LGR5-hi
Fib.ITGBL1-hi
Fib.CCL19-hi
advF
act.advF
Mesothelial
Peri.ADARB2-hi
Peri.COL25A1-hi
SMC
Aerocytes
gCap
act.gCap
Arterial
pVE.ABCC8-hi
pVE.RSPO3-hi
sVE.POSTN-hi
sVE.COL15A1-hi
act.sVE
Lymphatic EC
B cells
Plasma B
CD4+ T
CD8+ T
Tregs
NK
cMonocytes
monoMacs
intMacs
Macs.SPP1-hi
Macs.SPP1-lo
Macs.ISG-hi
Macs.MT-hi
alvMacs
act.alvMacs
cDC1
cDC2
Mast
Neutrophils
C
ILD-all; spatially constrained
Significant L-R pairs
0 10 20 30 40 50 60
Epithelial
Stromal
Endothelial
Immune
AT2
AT1
Aberrant trAT
SC.SCGB3A2-hi
SC.SCGB3A1-hi
Basal
Ciliated
alvF
act.alvF
MyoF.CTHRC1-lo
MyoF.CTHRC1-hi
Fib.LGR5-hi
Fib.ITGBL1-hi
Fib.CCL19-hi
advF
act.advF
Mesothelial
Peri.ADARB2-hi
Peri.COL25A1-hi
SMC
Aerocytes
gCap
act.gCap
Arterial
pVE.ABCC8-hi
pVE.RSPO3-hi
sVE.POSTN-hi
sVE.COL15A1-hi
act.sVE
Lymphatic EC
B cells
CD4+ T
CD8+ T
Tregs
cMonocytes
monoMacs
intMacs
Macs.SPP1-hi
Macs.SPP1-lo
Macs.ISG-hi
Macs.MT-hi
alvMacs
act.alvMacs
cDC1
cDC2
Neutrophils

Figure S7: Cellular interaction analyses, related to Figure 3.

A. Heatmap of total sum of interaction scores between cell types, representing the landscape of intercellular signaling in scRNA-seq atlas profiles of non-ILD controls. Significant ligand-receptor interaction scores were computed using *cellphonedb* with constraints on spatial communities.

B. As in **A**, but scores were computed without constraints on spatial communities.

C. As in **A,** but using scRNA-seq atlas profiles of all ILD patients, with constraints on spatial communities.

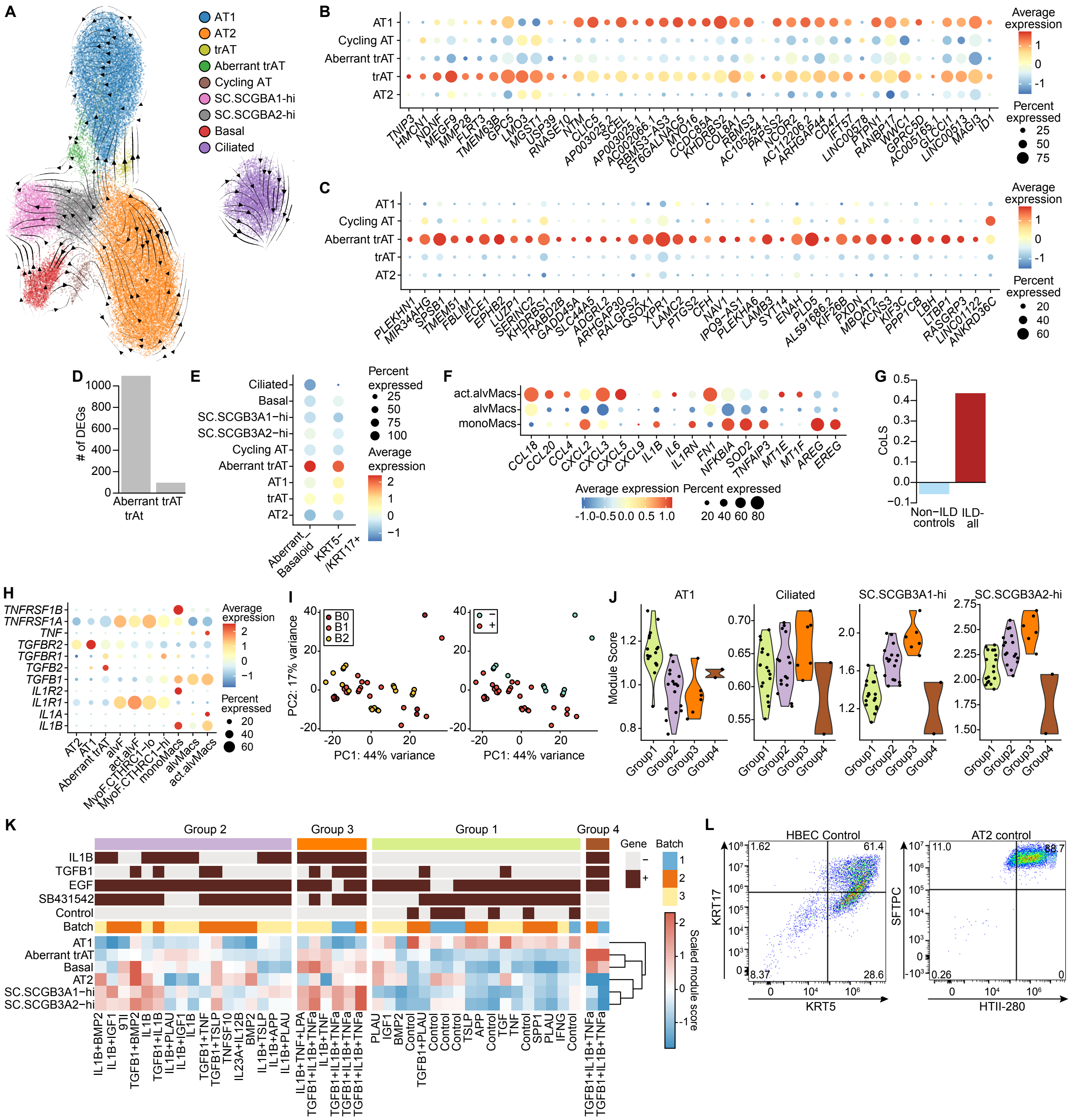
A
AT1
AT2
trAT
Aberrant trAT
Cycling AT
SC.SCGBA1-hi
SC.SCGBA2-hi
Basal
Ciliated
B
C
D
# of DEGs
Aberrant trAT
trAt
E
F
G
CoLS
Non−ILD controls
ILD-all
H
I
PC1: 44% variance
PC2: 17% variance
J
Module Score
Group1
Group2
Group3
Group4
K
Group 2
Group 3
Group 1
Group 4
L
HBEC Control
AT2 control
KRT17
KRT5
SFTPC
HTII-280

Figure S8: Identification and validation of aberrant trAT cell programs, related to Figure 4.
A. UMAP of epithelial cells from snRNA-seq data embedded with RNA velocity vector fields, suggesting that Aberrant trAT and trAT cells differentiate from AT2 cells.
B-C. Dot plots of expression of selected (**B**) trAT-enriched and (**C**) Aberrant trAT-enriched markers.
D. Bar plot of number of differentially expressed genes enriched in Aberrant trAT and trAT cells.
E. Cell type signatures of previously published aberrant epithelial populations mapped to current snRNA-seq atlas.
F. Dot plots of expression of selected chemokines, cytokines and growth factors in selected cell types in the scRNA-seq data.
G. Bar plot of co-localization scores (CoLS) of AT1 and AT2 cells in non-ILD controls and all ILD patients.
H. Dot plots of expression of selected TGF-β, IL-1β and TNF-α family ligands and receptors in selected cell types in the scRNA-seq data.
I. Principal component analysis of transcriptional profiles in Figure 3F labeled by batch and presence (+) or absence (-) of TGF-β signaling inhibitor SB431542 in the culture media.
J. Violin plot of groupwise cell type-specific signature scores computed on transcriptome profiles using cell type-specific markers from the scRNA-seq atlas.
K. Heatmap of cell type-specific signatures for each stimulation.
L. FACS plots showing (left) validating gates for KRT5 and KRT17 in HBECs and (right) a strategy for isolating AT2 cells from human lung tissue by gating for SPTPC+ HTII280+ cells.

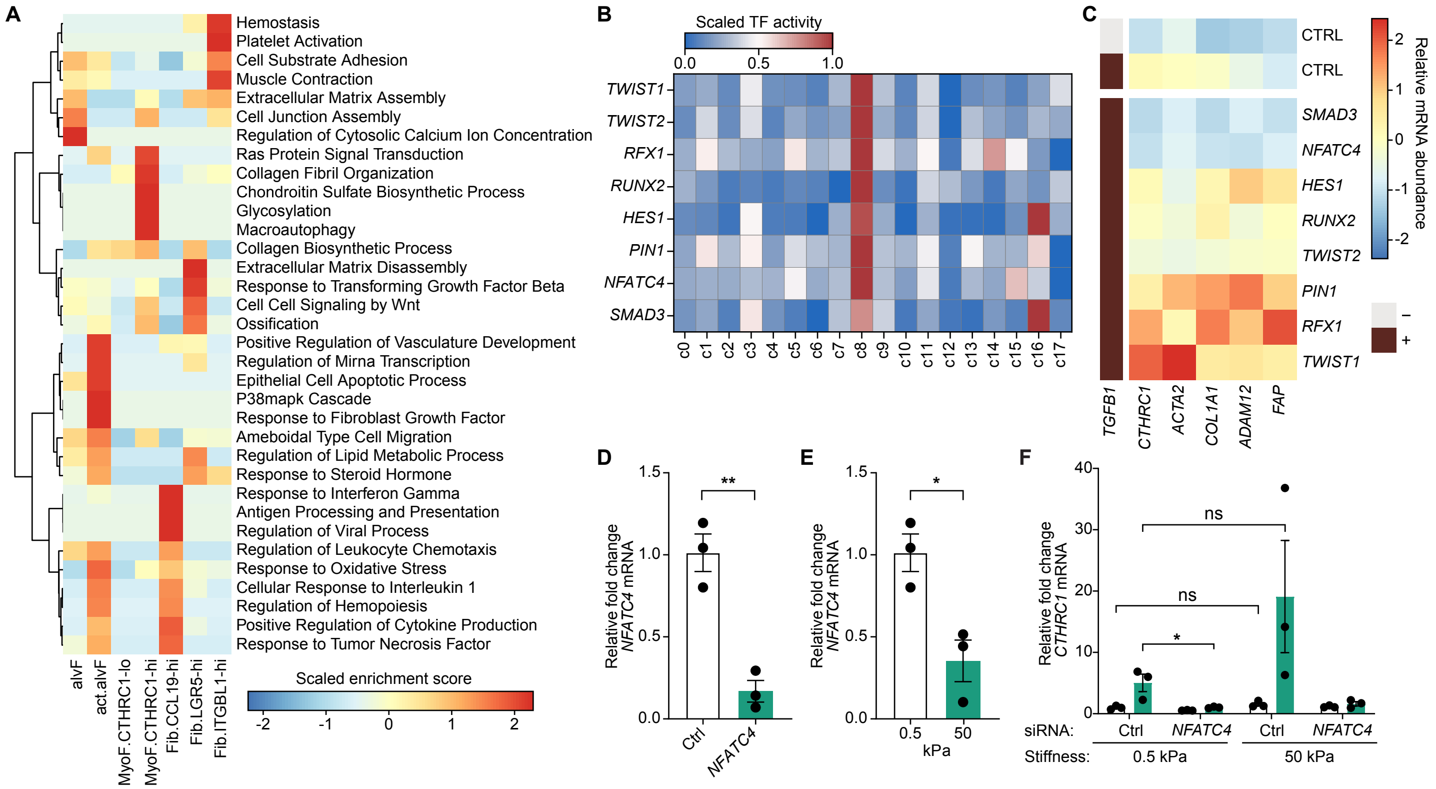
A
Hemostasis
Platelet Activation
Cell Substrate Adhesion
Muscle Contraction
Extracellular Matrix Assembly
Cell Junction Assembly
Regulation of Cytosolic Calcium Ion Concentration
Ras Protein Signal Transduction
Collagen Fibril Organization
Chondroitin Sulfate Biosynthetic Process
Glycosylation
Macroautophagy
Collagen Biosynthetic Process
Extracellular Matrix Disassembly
Response to Transforming Growth Factor Beta
Cell Cell Signaling by Wnt
Ossification
Positive Regulation of Vasculature Development
Regulation of Mirna Transcription
Epithelial Cell Apoptotic Process
P38mapk Cascade
Response to Fibroblast Growth Factor
Ameboidal Type Cell Migration
Regulation of Lipid Metabolic Process
Response to Steroid Hormone
Response to Interferon Gamma
Antigen Processing and Presentation
Regulation of Viral Process
Regulation of Leukocyte Chemotaxis
Response to Oxidative Stress
Cellular Response to Interleukin 1
Regulation of Hemopoiesis
Positive Regulation of Cytokine Production
Response to Tumor Necrosis Factor
alvF
act.alvF
MyoF.CTHRC1-lo
MyoF.CTHRC1-hi
Fib.CCL19-hi
Fib.LGR5-hi
Fib.ITGBL1-hi
Scaled enrichment score
-2
-1
0
1
2
B
Scaled TF activity
0.0
0.5
1.0
TWIST1
TWIST2
RFX1
RUNX2
HES1
PIN1
NFATC4
SMAD3
c0
c1
c2
c3
c4
c5
c6
c7
c8
c9
c10
c11
c12
c13
c14
c15
c16
c17
C
CTRL
CTRL
SMAD3
NFATC4
HES1
RUNX2
TWIST2
PIN1
RFX1
TWIST1
TGFB1
CTHRC1
ACTA2
COL1A1
ADAM12
FAP
Relative mRNA abundance
2
1
0
-1
-2
–
+
D
Relative fold change
NFATC4 mRNA
1.5
1.0
0.5
0.0
**
Ctrl
NFATC4
E
Relative fold change
NFATC4 mRNA
1.5
1.0
0.5
0.0
*
0.5
50
kPa
F
Relative fold change
CTHRC1 mRNA
40
30
20
10
0
ns
ns
*
siRNA:
Ctrl
NFATC4
Ctrl
NFATC4
Stiffness:
0.5 kPa
50 kPa

Figure S9: Regulation of fibroblast gene programs, related to Figure 5.

A. Heatmap of scaled enrichment scores of selected gene ontology (GO) pathways enriched in fibroblast cell types. Enrichment analysis was performed using *clusterprofiler* R package. Enrichment scores were derived by -log (P-values) and mean scaled.

B. Heatmap of selected transcription factors (TFs) enriched in estimated activity in cellular communities identified in the spatial transcriptomics data.

C. Heatmap of ECM-related genes detected by qPCR after siRNA-mediated knockdown of indicated TFs (X-axis).

D-E. Levels of *NFATC4* mRNA in healthy fibroblasts detected by qPCR (**D**) without (Ctrl) or with knockdown of *NFATC4* (**E**), stimulated with TGF-β, and cultured in soft (0.5 kPa) or rigid (50 kPa) substrates. *P < 0.05, **P < 0.001. Error bars represent SEM. Unpaired Student's t-test (two-sided). n = 3.

F. Relative levels of *CTHRC1* mRNA after TGFβ stimulation of IPF patient-derived Ctrl and *NFATC4*-knockdown fibroblasts cultured in soft (0.5 kPa) or rigid (50 kPa) substrates. *P < 0.05. Error bars represent SEM. Unpaired Student's t-test (two-sided). n = 3.
