# Supplementary material for: Spatial transcriptomics reveals altered communities and drivers of aberrant epithelia and pro-fibrotic fibroblasts in interstitial lung diseases
Source: Cell Genom. 2026 Jan 22;6(3):101066. doi: 10.1016/j.xgen.2025.101066 (PMC12985369; doi:10.1016/j.xgen.2025.101066)
Supplement: Document S2. Article plus supplemental information [file mmc4.pdf]

Cell Genomics

Article

Spatial transcriptomics reveals altered communities and drivers of aberrant epithelia and pro-fibrotic fibroblasts in interstitial lung diseases

Graphical abstract

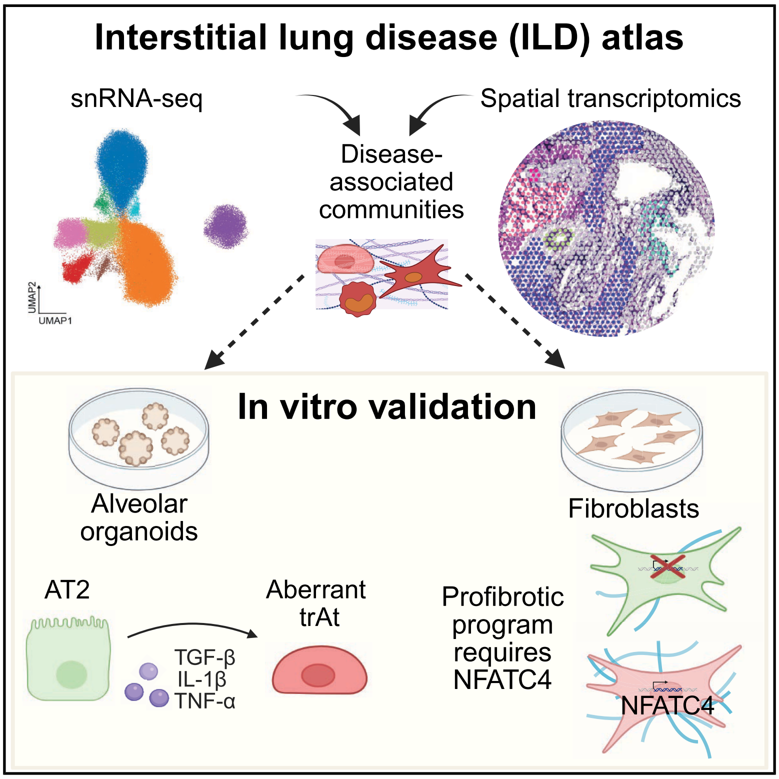

Highlights

- Multimodal atlas (snRNAseq/spatial/histology) reveals gradients in fibrotic activity
- Fibrotic niches harbor *CTHRC1*-hi fibroblasts and aberrant epithelial cells
- TGF-β, IL-1β, and TNF-α drive maladaptive aberrant transitional cell differentiation
- Transcription factor *NFATC4* regulates pro-fibrotic gene induction in fibroblasts

Authors

Alok Jaiswal, Tristan Kooistra, Vladislav Pokatayev, ..., Agostinho Carvalho, Benjamin D. Medoff, Ramnik J. Xavier

Correspondence

xavier@molbio.mgh.harvard.edu

In brief

Using single-nucleus RNA sequencing and spatial transcriptomics, Jaiswal et al. identify disease-associated cell communities that include aberrant transitional alveolar epithelium and pro-fibrotic fibroblasts. Using *in vitro* culture systems, they validate molecular drivers of these pathogenic cell states, including pro-inflammatory cytokines and the transcription factor *NFATC4*.

Jaiswal et al., 2026, Cell Genomics *6*, 101066
March 11, 2026 © 2025 The Author(s). Published by Elsevier Inc.
https://doi.org/10.1016/j.xgen.2025.101066

CellPress

Cell Genomics

CellPress
OPEN ACCESS

Article

Spatial transcriptomics reveals altered communities and drivers of aberrant epithelia and pro-fibrotic fibroblasts in interstitial lung diseases

Alok Jaiswal,[1,2] Tristan Kooistra,[3,4] Vladislav Pokatayev,[1,2,5] Hélder N. Bastos,[6,7,8] Rita F. Santos,[7,9] Tresa R. Sarraf,[3,4] Åsa Segerstolpe,[10] Crystal Lin,[10] Liat Amir-Zilberstein,[10] Shaina Twardus,[11] Kevin Shannon,[11] Shane P. Murphy,[10] Rachel Knipe,[3,4] Ingo K. Ganzleben,[3,4] Katharine E. Black,[3,4] Toni M. Delorey,[10] Daniel B. Graham,[1,2,5,10] Yin P. Hung,[12] Lida P. Hariri,[3,12] Jacques Deguine,[1,10] Agostinho Carvalho,[13,14] Benjamin D. Medoff,[3,4] and Ramnik J. Xavier[1,2,5,10,15,16,17,*]

[1]Broad Institute of MIT and Harvard, Cambridge, MA 02142, USA
[2]Department of Molecular Biology, Massachusetts General Hospital, Boston, MA 02114, USA
[3]Division of Pulmonary and Critical Care Medicine, Massachusetts General Hospital and Harvard Medical School, Boston, MA 02114, USA
[4]Center for Immunology and Inflammatory Diseases, Massachusetts General Hospital and Harvard Medical School, Boston, MA 02114, USA
[5]Center for Computational and Integrative Biology, Massachusetts General Hospital and Harvard Medical School, Boston, MA 02114, USA
[6]Department of Pneumology, Hospital de São João, 4200-319 Porto, Portugal
[7]i3S – Instituto de Investigação e Inovação em Saúde, Universidade do Porto, 4200-135 Porto, Portugal
[8]Faculty of Medicine/RISE-Health, University of Porto, 4200-319 Porto, Portugal
[9]School of Health Sciences – Polytechnic of Porto, 4200-072 Porto, Portugal
[10]Klarman Cell Observatory, Broad Institute of MIT and Harvard, Cambridge, MA 02142, USA
[11]Division of Gastroenterology, Massachusetts General Hospital and Harvard Medical School, Boston, MA 02114, USA
[12]Department of Pathology, Massachusetts General Hospital and Harvard Medical School, Boston, MA 02114, USA
[13]Life and Health Sciences Research Institute (ICVS), School of Medicine, University of Minho, 4710-057 Braga, Portugal
[14]ICVS/3B's - PT Government Associate Laboratory, 4710-057 Braga/Guimarães, Portugal
[15]Center for the Study of Inflammatory Bowel Disease, Massachusetts General Hospital, Boston, MA 02114, USA
[16]The Gene Lay Institute of Immunology and Inflammation, Brigham and Women's Hospital, Massachusetts General Hospital, Harvard Medical School, Boston, MA 02115, USA
[17]Lead contact
*Correspondence: xavier@molbio.mgh.harvard.edu
https://doi.org/10.1016/j.xgen.2025.101066

SUMMARY

Interstitial lung diseases (ILD) are characterized by fibrotic scarring of the lung parenchyma with remarkably unfavorable prognosis. Using single-nucleus RNA sequencing and spatial transcriptomics, we generated a comprehensive cellular network of the distal lung and its alterations in fibrosis. Integration with histopathology revealed that the transformation of normal parenchyma into fibrotic tissue is accompanied by ectopic bronchiolization and decellularization. Areas of active fibrosis were characterized by co-localization of pro-fibrotic *CTHRC1*-hi fibroblasts and aberrant transitional epithelial cells. We modeled this maladaptive differentiation of alveolar epithelial cells using organoids, demonstrating that all three pro-inflammatory ligands present in this pathogenic niche, TGF-β, IL-1β, and TNF-α, are jointly required for their induction. Additionally, we identified a requirement for the transcription factor *NFATC4* during myofibroblast differentiation driven by soluble factors or mechanosensing. Collectively, this work identifies essential molecular drivers of the cellular interactions underlying lung fibrosis.

INTRODUCTION

Fibrosis is a pathology characterized by the excessive deposition of extracellular matrix (ECM) components, leading to stiffness, loss of tissue integrity, and ultimately compromised organ function.[1,2] Fibrosis can affect many organs[1,3] and is primarily an outcome of dysfunctional tissue repair. Scarless healing requires a coordinated response among cells localized in the wound bed. Injury-induced transient epithelial cells initiate the inflammatory response phase by recruiting neutrophils and macrophages; subsequently, fibroblasts are activated to synthesize ECM components, promoting the reestablishment of tissue integrity.[1,4–6] Recent studies have shown the importance of fibroblasts in tuning the dynamics of spatial organization in efficient wound repair.[7] However, repetitive tissue injury can lead to persistent inflammation and ECM deposition, shifting the balance from healing toward fibrosis.[8]

The distal lung alveolar epithelial lining consists of alveolar type II (AT2) and type I (AT1) cells. AT2 cells secrete surfactants to regulate alveolar surface tension and prevent alveolar collapse,

© 2025 The Author(s). Published by Elsevier Inc.
This is an open access article under the CC BY license (http://creativecommons.org/licenses/by/4.0/).

while AT1 cells are flat and thin, facilitating gas exchange. AT2 cells also function as resident stem cells, differentiating into AT1 cells to maintain the epithelial barrier. Progressive scarring of the distal parenchyma reduces tissue compliance and destroys gaseous-exchange units, causing respiratory failure.[9] Lung fibrosis may result from a diverse group of interstitial lung diseases (ILDs), including idiopathic pulmonary fibrosis (IPF).[10] Single-cell RNA sequencing (scRNA-seq)-based profiling of human fibrotic lungs has identified several cell types highly enriched in ILD tissues, including *CTHRC1*+ pro-fibrotic fibroblasts, bronchial epithelial and endothelial populations, and *KRT5*−/*KRT17*+ aberrant transitional epithelial cells.[11–17] Transitional epithelial cells exhibiting an intermediate phenotype between AT2 and AT1 cells with basal-cell-like morphology have also been observed following acute lung injury,[18–21] highlighting the parallels between post-injury lung repair and some aspects of pulmonary fibrosis. Spatial transcriptomics allows reconstruction of tissue organization in order to interrogate how spatial neighborhoods and intercellular communication are altered in lung fibrosis.[22] Although recent studies have begun to explore the spatial transcriptomic landscape of ILD,[23–25] the spatial topography of cellular neighborhoods around these disease-enriched populations and their drivers are not well understood.

Here, using single-nucleus RNA sequencing (snRNA-seq) of lung tissues from 36 subjects, alongside an integrative meta-analysis of published scRNA-seq studies, we first create a comprehensive census of the cell types and cellular states present in the distal ILD lung. Using 10× Visium-based spatial transcriptomics profiling, we then map the spatial topography of cellular neighborhoods in non-ILD control and ILD lungs. We integrate histopathology, single-cell transcriptomics, and spatial transcriptomics to reveal that the gradual transformation of normal lung parenchyma into fibrotic tissue is accompanied by a transient wave of pro-fibrotic activity, ultimately leading to a tissue with decreased cellularity. Further, we systematically characterize the intercellular communication landscape within healthy and diseased tissues to identify a pathogenic niche of aberrant epithelial cells interacting with *CTHRC1*+ myofibroblasts. By analyzing the molecular programs and composition of cellular neighborhoods around aberrant transitional epithelial cells, and following screening in AT2 organoids, we identify and validate pro-inflammatory signaling from the three ligands TGF-β, IL-1β, and TNF-α as responsible for driving the maladaptive AT2 differentiation toward the aberrant state. Lastly, we identify a mechanosensitive transcription factor, *NFATC4*, involved in regulating the differentiation of pro-fibrotic *CTHRC1*-hi myofibroblasts, and validate that the loss of *NFATC4* limits collagen deposition in an *in vitro* model.

RESULTS

An snRNA-seq-based census of the distal lung reveals altered cellular states and compositional rewiring during ILD

Frozen lung tissues obtained from 36 individuals across two sites (Massachusetts General Hospital [MGH] and Hospital de São João [HSJ], Portugal) were subjected to snRNA-seq profiling. The cohort included data from 25 ILD patients, 7 non-ILD control subjects, and 4 non-ILD diseased patients diagnosed with other chronic lung conditions (STAR Methods and Table S1). ILD subjects were further categorized into patients with IPF (n = 6), patients with other fibrotic forms of ILD (non-IPF fibrotic ILD, n = 17), and those with non-fibrotic ILD (n = 2) (Figure 1A). We also incorporated published snRNA-seq datasets generated from non-ILD control subjects (n = 9)[26,27] into our analyses (Figure 1A).

After quality control and clustering (STAR Methods), we retained a dataset of 227,680 nuclei across 48 distinct cell types (Figures 1B–1E; S1A–S1D). Epithelial cells comprised approximately 57%, endothelial cells 13%, stromal cells 13%, and immune cells 17% of the total abundance (Figure S1E), which was largely consistent across cohorts. Moreover, meta-analysis of ∼890,000 cells from published scRNA-seq datasets,[11–15] analyzed using an identical workflow yielded clustering of cellular states in broad agreement with the snRNA-seq atlas (Figures S2A–S2I). The scRNA-seq datasets exhibited more variability in tissue composition, possibly explained by study-specific differences in tissue collection and processing (Figure S2J). We observed notably improved recovery of AT1 cells with snRNA-seq (Figure S2K); these cells are likely prone to under-sampling in scRNA-seq due to their elongated morphology. Notably, only by snRNA-seq were we able to identify several clusters of transitional alveolar epithelial cells (trAT), including Aberrant trAT cells, which exhibit elevated *SOX9* expression (Figure S1A). While *SOX9* is transiently expressed in injury-induced regenerating epithelial cells, its persistent expression is linked to fibrosis.[8]

To parse the contributions of technical factors and disease states to cell type composition, we performed a principal component analysis (PCA) of cell-type frequencies. The cellular composition of non-ILD control tissues was significantly different from that of ILD patient tissues (PERMANOVA $R^2 = 0.44$, $p < 10^{-3}$); however, we did not observe statistical differences in composition between IPF and other ILD disease subtypes (Figure 1F). Differences in 10X chemistry, site of collection, study, and method of tissue collection did not significantly contribute to compositional changes (Figure S3A). The loadings of each cell type on the top principal components suggested contributions of distinct cell types to disease (Figure S3B), with cells associated with alveolar spaces (AT1, AT2, and alvMacs) enriched in non-ILD communities.

To further explore cell-type-specific differential abundances between non-ILD controls and all ILD patients, including IPF patients, we combined the patient-level composition data from both modalities (STAR Methods). After controlling for study-specific and technical effects, we identified changes in several cell types that recapitulate previous observations,[11–15] as well as previously unreported changes (Figures 1G; S3C and S3D). We observed a significant loss of alveolar AT2 and AT1 cells in disease (false discovery rate [FDR] <20%), owing to destruction of the respiratory unit. This was accompanied by an increased proportion of ciliated, basal, and secretory club cells (SCs) expressing higher levels of *SCGB3A2* or *SCGB3A1* (Figures 1G; S3C). We observed that Aberrant trATs were expanded in ILD, but trATs remained unchanged. trATs expressed several genes enriched in AT2 and AT1 cells, whereas Aberrant trAT expressed higher levels of the basal cell lineage factor *TP63* (Figures 1H; S1A). Although a previous study reported that transient cells

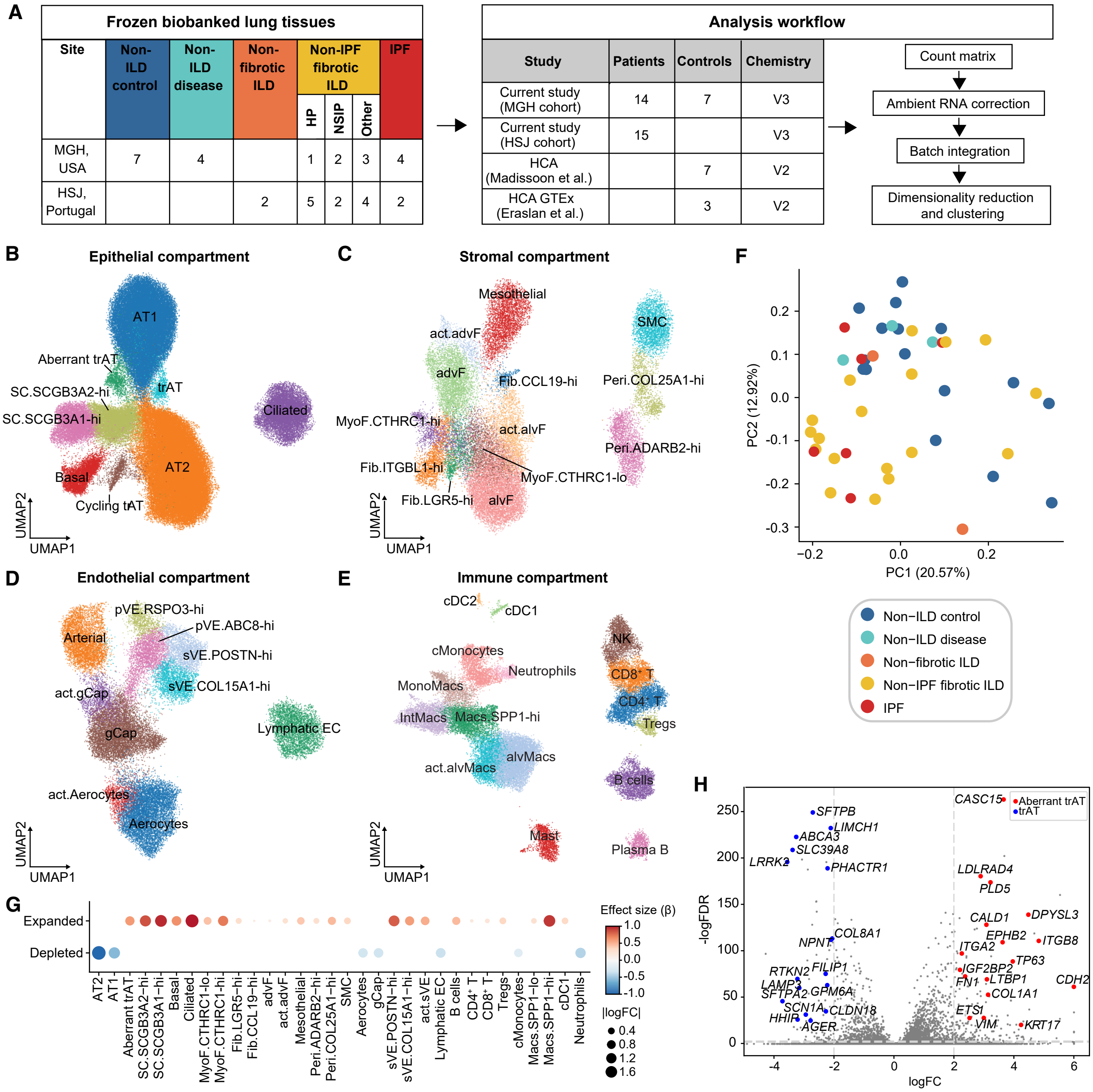

Figure 1. An snRNA-seq-based census of distal lung reveals altered cellular states and compositional rewiring during ILD

(A) Overview table of snRNA-seq cohort composition and analysis pipeline description.

(B–E) Uniform manifold approximation and projection for dimension reduction (UMAP) representations of the epithelial (B), stromal (C), endothelial (D), and immune (E) compartments in our snRNA-seq dataset. SC, secretory cell; alvF, alveolar fibroblast; advF, adventitial fibroblast; act., activated cell; gCap, general capillary; Fib., fibroblast; MyoF., myofibroblast; Peri., pericyte; SMC, smooth muscle cells; pVE, pulmonary venous endothelial; sVE, systemic venous endothelial; cMonocytes, classical monocyte; monoMacs, monocyte derived macrophage; Macs., macrophage; intMacs, interstitial macrophage; alvMacs, alveolar macrophage; cDC, conventional dendritic cell.

(F) Principal component analysis (PCA) of cell type composition. Each dot represents one sample, colored by diagnostic category. Principal component loadings and other categories are presented in Figures S3A–S3B.

(G) Dot plot showing effect size and absolute log2 fold change (logFC) for cell type abundance by disease status. Blue indicates enrichment in non-ILD controls and red indicates enrichment in ILD patients (FDR <20% by *scCODA*).

(H) Volcano plot showing differentially expressed genes (Wilcoxon test, adjusted $p < 0.05$) between trAT and Aberrant trAT cells. Selected genes are highlighted.

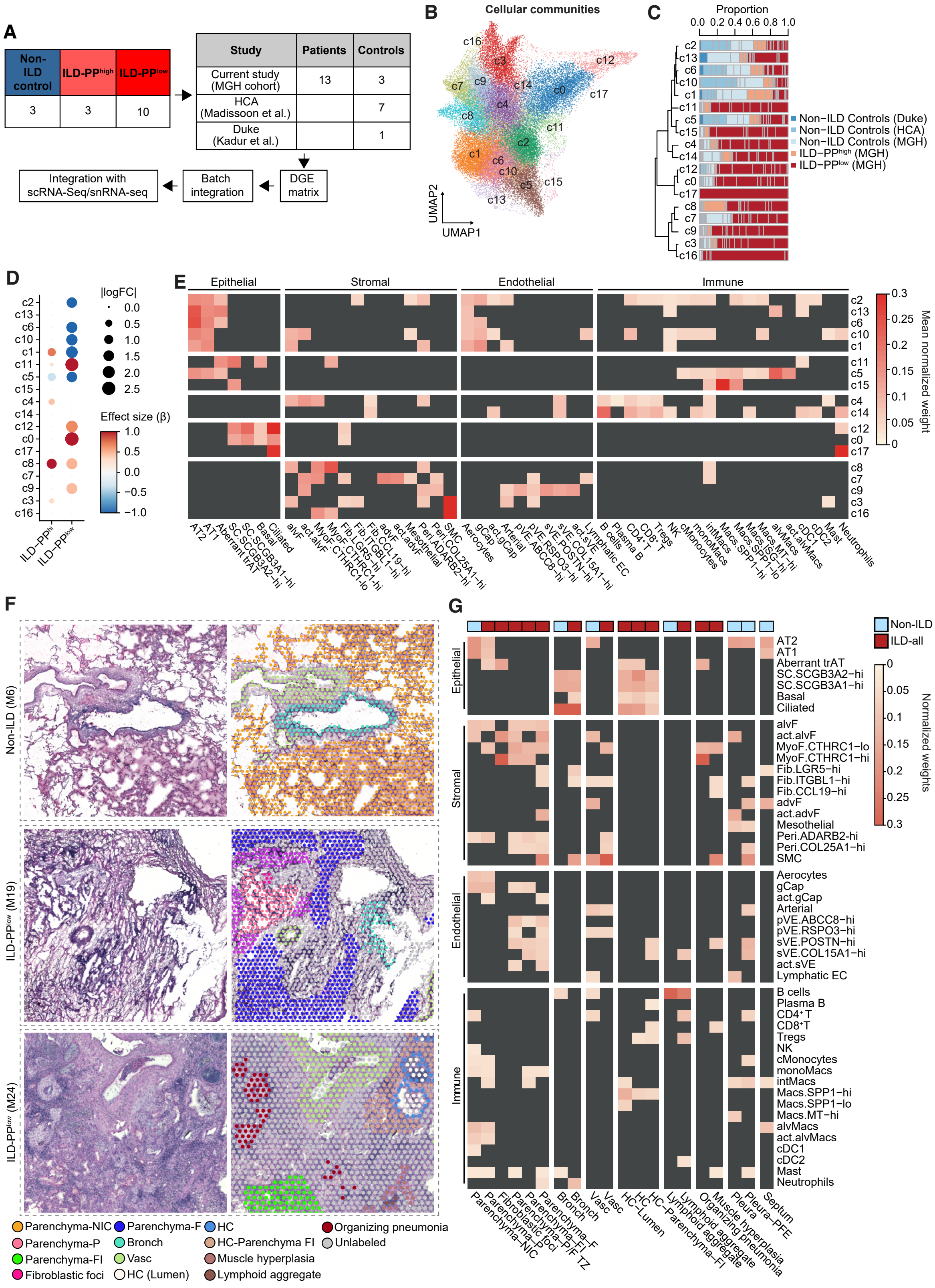

(legend on next page)

CellPress
OPEN ACCESS

exhibiting signatures shared with AT2, AT1, and SC.*SCGB3A2*-hi cells were expanded in lung injury and IPF,[28] trATs were not expanded in ILD patients in our data.

Within the stromal and endothelial compartments, our analysis revealed distinct cell types and their finer cellular states that changed with disease. We identified major fibroblast subsets, such as alveolar fibroblasts (alvF) and adventitial fibroblasts (advF), along with myofibroblasts (MyoF) expressing *FAP* and elevated levels of *ACTA2* (Figures 1C; S1B; S2D). We further delineated MyoF into *CTHRC1*-hi and *CTHRC1*-lo subsets based on their expression levels of *CTHRC1* and other genes involved in ECM organization. Similarly, we identified multiple subsets of pulmonary venous endothelial (pVE) cells, *RSPO3*-hi and *ABCC8*-hi; systemic venous endothelial (sVE) cells, *POSTN*-hi and *COL15A1*-hi (Figures 1D; S2F); and perivascular pericytes, *COL25A1*-hi and *ADARB2*-hi (Figures 1C; S2D), uncovering previously unappreciated and potentially functionally relevant heterogeneity in the vasculature of the distal lung. Importantly, snRNA-seq and scRNA-seq datasets agreed on the cell-type-specific signatures for both stromal and endothelial cells (Figures S2E and S2G).

We noted a significant loss of Aerocytes and general capillaries (gCap) (at FDR <20%) (Figures 1G; S3D) in ILD, accompanied by expansion of the bronchial sVE cell, pericytes, and MyoF subsets (Figures 1G; S3D) as observed previously.[12,29] We also observed enrichment of Fib.*LGR5*-hi fibroblasts, mesothelial cells, and smooth muscle cells (SMCs) in ILD (Figures 1G; S3D). In the immune compartment (Figures 1E; S2H), we observed significant expansion of *SPP1*+ macrophages (Macs *SPP1*-hi/lo), memory B cells, and regulatory T cells (Tregs) (Figures 1G; S3C and S3D).

In both atlases, we observed activated states (act.) of many cell types, including act.Aerocytes, act.gCaps, act.alvFs, act.advFs, act.alvMacs (alveolar macrophages) (Figures 1C–1E; S2D–S2I), and act.sVE cells (Figure S2F). We defined these as activated cell subsets due to higher expression of genes involved in stress responses (*IER3* and *HIF1A*), pro-inflammatory processes (NF-κB members, *FOSB*, and *JUNB*), immune trafficking (chemokines and adhesion receptors), and senescence[30] (*CDKN1A*, *RELA*, *IL6*, and *UBB*) relative to their normal counterparts (Figures S3E and S3F), suggesting they may be induced by the inflammatory milieu of the diseased tissues. Some of these activated cell states, such as act.advFs and act.sVE, were also expanded in ILD (Figures 1G; S3D).

Altogether, we generated a comprehensive profile of cell types and states that are altered in ILD, revealing a systematic expansion of pro-fibrotic fibroblasts and bronchovascular cells in ILD patients. We also observed activated cell states exhibiting a pro-inflammatory and stress-response gene program, underscoring the role of inflammation in lung fibrosis.

Extensive remodeling of the spatial topography of distal lung in ILD

To investigate the changes in tissue organization caused by ILD, we performed spatial transcriptomics on 16 flash-frozen distal lung tissues obtained from 15 individuals, including 13 blocks from end-stage ILD explants and 3 from non-ILD control subjects, which showed no evidence of radiological abnormalities (Figures 2A and S4). We supplemented the number of non-ILD controls by incorporating data generated in previous studies[26,28] (Figure 2A). After performing quality control, dimensionality reduction, clustering, and accounting for batch effects, we recovered 49,841 spots (42,119 generated in the current study), which segregated into 18 clusters representing distinct spatial communities or tissue domains (c0–c17) (Figure 2B). These spatial communities exhibited distinct gene expression patterns (Figure S5A) and differential abundance by grade of fibrosis (Figures 2B–2D; S5B), leading us to group ILD samples based on the fraction of preserved parenchyma (PP): ILD-PP^{high} for samples with $\geq$50% of spots annotated as PP by histologic examination and ILD-PP^{low} otherwise (Figure S4).

To gain more insight into the cellular composition of the spatial communities, we integrated the spatial transcriptomic data with sn/scRNA-seq atlases to map the spatial location of each cell type. There was a general concordance in estimates of the relative proportions of cell types at each spot using both atlases (Figure S5C), except for the fibroblast subsets, likely due to their lower representation of fibroblasts in the snRNA-seq data. For subsequent analyses, we therefore utilized estimates derived using the scRNA-seq atlas (STAR Methods).

Differential abundance analysis of communities suggested that c1, c2, c6, and c10 were significantly depleted (at FDR <20%) in ILD-PP^{low} samples (Figures 2D; S5B). Compositionally,

Figure 2. Extensive remodeling of the spatial topography of the distal lung in ILD

(A) Spatial transcriptomics data collection and analysis pipeline description.

(B) UMAP embedding and annotation of spot level transcriptomics profiles, with each cluster defined as a distinct cellular community.

(C) Normalized proportions of sample-level cellular communities by disease status and tissue histology. Each bar indicates one sample colored by cohort and, for ILD samples, by histological annotation: ILD-PP^{high} includes tissues with $\geq$ 50% of spots annotated as PP; ILD-PP^{low} includes tissues with <50% of spots annotated as PP. Communities are organized based on the hierarchical clustering of their aggregate gene expression (left dendrogram).

(D) Dot plot showing effect size and absolute log2 fold change (logFC) for cellular community abundances in ILD-PP^{high} and ILD-PP^{low} samples vs. non-ILD controls. Blue indicates enrichment in non-ILD controls, red in ILD-PP^{high}/ILD-PP^{low} samples (FDR <20% by *scCODA*).

(E) Mean robust cell-type decomposition (RCTD)-estimated normalized weights of each cell type and cellular community using the scRNA-seq reference atlas. Legend color scale represents the mean normalized weight in the respective category for statistically significant (Benjamini-Hochberg adjusted p < 0.05) comparisons; insignificant comparisons are colored dark gray.

(F) Schema of identification of anatomical and histopathological features in representative hematoxylin and eosin (H&E) images including raw images (left) and images overlayed with Visium spots (right, colored by annotation as indicated in the legend).

(G) Mean RCTD-estimated normalized weights of each cell type and histopathological structure using the scRNA-seq reference atlas. Legend color scale represents the mean normalized weight in the respective category for statistically significant (Benjamini-Hochberg adjusted p < 0.05) comparisons; insignificant classes are colored dark gray.

In (F) and (G), NIC, non-ILD control; P, preserved zone; F, fibrotic zone; TZ, transitional zone; FI, fibrotic inflamed zone; Bronch, bronchiole; Vasc, vasculature; HC, honeycomb cyst; ILD-all, ILD-PP^{high} and ILD-PP^{low} samples combined.

these communities were enriched for AT2, AT1, and alvF (Figures 2E; S5D) (adjusted $p < 0.05$, Student's t test), consistent with the loss of PP in these samples. gCaps and Aerocytes were also enriched in these communities, consistent with a previous report[31] suggesting that the human alveolar endothelium is a mosaic mesh of two distinct specialized capillary cell types. In contrast, c11 was most expanded in ILD-PP^{low} samples and was composed of *CTHRC1*-hi MyoF and multiple epithelial populations, including Aberrant trAT, SC.*SCGB3A2*-hi, and basal cells, denoting the spatial association between these disease-enriched populations and disease severity (Figures 2D and 2E). c0 and c12 were enriched for basal, ciliated, and SC epithelial cells, marking the distal bronchiolar epithelium, as well as *LGR5*-hi fibroblasts, marking the peribronchiolar space. Interestingly, c0 and c12 were enriched only in ILD patients (Figures 2D; S5B), revealing the extent of alveolar parenchyma takeover by ectopic epithelial bronchiolization.

Communities c3 and c9 expanded in ILD-PP^{high} and ILD-PP^{low}, respectively (Figures 2D; S5B), were compositionally enriched for larger non-capillary vasculature (pVE, sVE, and arterial), advF, and SMCs (Figures 2E, S5D), which are known to be localized in the adventitia of bronchovascular bundles in homeostasis. c4 was enriched for B and T lymphocytes, along with alvF and *CCL19*-hi fibroblasts (Figure 2E), indicative of regions of lymphocyte infiltration. Further, c4 was significantly expanded in ILD-PP^{high} tissues and nominally, though not significantly, in ILD-PP^{low} (Figures 2D; S5B), highlighting areas of inflammation in less fibrotic areas.

In our scRNA-seq atlas, we observed two clusters of disease-enriched *SPP1*+ macrophages defined by *SPP1* expression levels, *SPP1*-lo and *SPP1*-hi, suggesting that these cells exist along a polarization spectrum (Figures 1G; S1D; S3C). *SPP1*+ macrophages are monocyte-derived macrophages associated with fibrotic scarring in many tissues.[32–37] Both *SPP1*-hi/lo macrophage subsets were enriched in communities c5 and c15, with c5 being co-inhabited by alveolar macrophages, AT2 and AT1 cells, and significantly reduced in both ILD-PP^{high} and ILD-PP^{low} tissues. However, c15 showed a trend for expansion in ILD patients and was also enriched for secretory SC.*SCGB3A2*-hi cells (Figures 2D and 2E; S5B). The appearance of bronchiolar epithelial cells alongside *SPP1*-hi macrophages hints at ongoing ectopic bronchiolization (Figure 2E).

In summary, our data provide insights into the spatial topography of distal lung cell types and their relation to distinct spatial communities and also highlight the magnitude of tissue remodeling inflicted by ILD.

An integrated histo-cellular map reveals links between tissue morphology and cellular communities

To better understand disease-related changes in tissue topology and organization, we leveraged a top-down, histopathology-based approach to connect cell types to distinct anatomical regions (alveolar parenchyma, distal airways, and bronchovascular bundles and pleura), and to pathological morphologies associated with ILD (fibroblastic foci, honeycomb cysts [HCs], muscle hyperplasia, lymphoid aggregates, and organizing pneumonia) (Figures 2F; S6A; see STAR Methods). We manually annotated 30,988 spots into distinct categories, averaging ∼62% of total spots annotated per sample, with distal parenchyma being the most frequently annotated feature (Figures S6B and S6C).

As expected, parenchyma from non-ILD controls (Parenchyma-NIC) was enriched for alveolar epithelial AT2 and AT1 cells (Figures 2G; S6D), which were depleted in the parenchyma from ILD tissues. ILD parenchyma was categorized as preserved zone (Parenchyma-P), fibrotic zone (Parenchyma-F), transitional zone (Parenchyma-P/F TZ; located between the former two), or inflamed fibrotic zone (Parenchyma-FI). Aberrant trAT cells were significantly enriched in Parenchyma-P and fibroblastic foci (adjusted $p < 0.05$, Student's t test), consistent with abnormal epithelial remodeling in these regions. MyoF.*CTHRC1*-hi cells were enriched in fibroblastic foci, Parenchyma-FI, and Parenchyma P/F TZ, but not in Parenchyma-F, suggesting higher matrix remodeling activity ongoing in the remnant parenchymal tissues. In the transitional zone and fibrotic parenchyma, the alveolar capillary cells were substituted by larger venous endothelial cells (sVE and pVE) and SMCs, indicating a transformation driven by ectopic vascularization and smooth muscle proliferation (Figure 2G). While the fibrotic parenchyma was enriched for neutrophils, most immune cells, such as lymphocytes, monocytes, intMacs, and cDC1, were enriched in Parenchyma-NIC (Figure S6D). Parenchyma-F had a lower estimated number of total cells, indicating that as the tissue became fibrotic, it also became decellularized ($p < 0.001$, Wilcoxon test) (Figure S6E).

Hierarchical clustering of the transcriptomic profiles from annotated histopathological structures suggested that HCs resembled the bronchiolar epithelium (Figure S6F). Interestingly, the inflamed fibrotic adventitial tissue surrounding HCs, defined as HC-Parenchyma-FI, was significantly enriched for both cytotoxic T lymphocytes and sVE cells (Figure 2G). HCs were also enriched for Aberrant trAT cells (Figure 2G), suggesting abnormal epithelial remodeling happening in these areas. Notably, *CTHRC1*-hi MyoF were enriched in the fibroblastic foci, which are known to be located near HCs, but were absent in the HC-Parenchyma-FI, suggesting a heterogeneous distribution of fibrotic activity in HCs (Figures 2G; S6D). Surprisingly, organizing pneumonia was also enriched for *CTHRC1*-hi MyoF and was similar in transcriptional profile to fibroblastic foci (Figure S6F). However, differential expression analysis between the two regions revealed elevated expression of ECM-related genes in the fibroblastic foci, indicating that fibroblasts localized in the fibroblastic foci may have higher pro-fibrotic activity. Conversely, plasma cell- and macrophage-enriched genes were elevated in expression in organizing pneumonia, suggesting a more pro-inflammatory environment (Figure S6G).

Collectively, we generated a highly resolved view of the enriched cell types in different anatomical and pathological regions in the distal ILD lung. We decipher how changes in the cellular milieu are juxtaposed with changes in tissue architecture during the progressive fibrosis of the lung parenchyma, with most fibrotic regions showing limited pro-fibrotic activity, decellularization, ectopic vascularization, and smooth muscle proliferation.

Loss of tissue structure and organization in lung fibrosis

To enable comparisons between tissue organization in homeostasis and disease,[38] we constructed a network graph of cellular neighborhoods based on colocalization scores (STAR Methods).

CellPress
OPEN ACCESS

In non-ILD control tissues, clustering of the network revealed a graph structure consistent with the known anatomical structures of the distal lung (Figures 3A and 3B).

Assessment of the network structure in ILD-PP^{low} samples revealed significant rewiring of the cellular neighborhoods (Figure 3C). Quantifying the partitioning of the graph showed that diseased tissues had significantly lower network modularity ($p < 0.05$, Wilcoxon test) as demonstrated by an increased number of cross-cluster edges, suggesting a loss of the tissue compartmentalization that exists in the healthy lung (Figures 3A–3D). The loss of physical contact between AT1 cells and structural cells in the interstitium, i.e., alvF, Aerocytes, and gCaps, indicates a breakdown of the alveolar walls (Figures 3A and 3C). Concurrently, cells normally located in the alveolar interstitium were now much more likely to colocalize with systemic and pVE cells, possibly suggesting ectopic vascularization (Figure 3C). We also observed an increased number of edges between bronchial and alveolar epithelial cells, indicating epithelial bronchiolization of the alveoli. A community of DCs, T and B lymphocytes, and *CCL19*-hi fibroblasts also appeared in the ILD-PP^{low} samples, highlighting increased lymphoid aggregate formation and immune cell infiltration (Figure 3C). Overall, we find that progressive scarring disrupts the anatomical and functional compartmentalization in healthy lungs.

Distortions in the intercellular signaling landscape reveal contributors to disease pathogenicity

Next, we investigated how intercellular signaling is altered in ILD by integrating the scRNA-seq atlas with spatial communities, as cross-talk between cells is dictated by short-range signaling and physical proximity.[40–42] Utilizing the spatial communities defined earlier (Figure 2B), we constructed a signaling network based on ligand-receptor expression between cell types located within the same communities in non-ILD controls (STAR Methods). We observed interactions between alveolar epithelial cells, alvFs, and alveolar endothelial cells, but no interactions of these with cells residing in the bronchovascular bundles (Figure S7A). By contrast, the signaling network that was constructed without constraining on spatial proximity showed interaction edges between cell types located in distinct anatomical regions (Figure S7B).

Examination of the constrained signaling network in ILD patients revealed substantial rewiring of the signaling landscape (Figure S7C). AT2 and AT1 cells had significantly more edges with myeloid cells, specifically monocyte-derived macrophages (monoMacs), *SPP1*-hi/-lo macrophages, and alveolar macrophages. SC.*SCGB3A2*-hi cells also showed edges with *SPP1*-hi/lo macrophages. The most notable change was an increase in the number of interactions involving alvF, MyoF, or advF (Figure S7C). We also observed an increased number of significant edges between non-immune and immune cell types, highlighting the pro-inflammatory environment in diseased tissue. Further, the increased number of edges in the diseased signaling network suggests an increase in non-canonical intercellular signaling, mirroring the loss of anatomical compartmentalization (Figure 3C).

Centrality analysis of intercellular signaling networks in healthy and ILD subjects identified cell types contributing to distortions in the signaling landscape (Figure 3E). Surprisingly, AT2, AT1, alvF, gCap, Aerocytes, and alveolar macrophages occupied more influential positions in the signaling networks in ILD vs. healthy tissues (Figure 3E) despite being depleted in ILD (Figure 1G). Conversely, stromal cell types in the bronchovascular bundles, such as advF, sVE *COL15A1*-hi, and *COL25A1*-hi pericytes, while expanded in ILD tissues (Figure 1G), did not have increased influence in the ILD-specific signaling network (Figure 3E), suggesting that their expansion reflects a response to ongoing structural changes rather than an increased role in signaling. In contrast, Aberrant trAT and *CTHRC1*-hi/-lo MyoF occupied highly influential positions in the ILD communication network (Figure 3E). Likewise, bronchiolar epithelial basal cells and *SC.SCGB3A2*-hi cells, activated cells such as act.sVE and act.alvMacs, and macrophages like monoMacs and *SPP1*-hi/-lo subsets had higher centrality scores in ILD patients (Figure 3E). These cell types, with increased enrichment and influence in disease, could constitute the pathogenic cell types driving disease progression.

Aberrant trAT localization is linked to pro-fibrotic *CTHRC1*-hi fibroblasts

Given the increased influence of Aberrant trAT cells in the disease network, we further explored the epithelial compartment changes in ILD in more detail. Projection of estimated RNA velocity vector fields into the UMAP embedding supported that trAT and Aberrant trAT cells were intermediate stages of differentiation from AT2 to AT1 cells (Figure S8A). Notably, genes enriched for expression in trAT cells were also generally expressed in AT1 cells (Figures S8B and S8C), while Aberrant trAT cells had a more distinct transcriptome with unique genes (Figures S8C and S8D). While trAT cells were not enriched in diseased subjects, Aberrant trAT cells were significantly expanded (Figure 1G). Previous studies have reported abnormal *KRT5*−/*KRT17*+ epithelial cells in fibrotic lungs,[12,13] whose transcriptomic signature overlapped with those of Aberrant trAT cells (Figure S8E).

Next, we explored the spatial neighborhoods of AT2 and Aberrant trAT cells by colocalization analysis to gain insight into the processes driving their differentiation. Strikingly, the neighborhood of AT2 cells was significantly rewired in ILD vs. non-ILD tissues (Figures 4A and 4B). AT2 cells were found in physical proximity to neutrophils, *SPP1*-hi, and alveolar macrophages in non-ILD controls (Figure 4A), whereas in ILD, colocalization with alveolar macrophages and act.alvMacs increased dramatically (Figure 4B). act.alvMacs expressed higher levels of pro-inflammatory chemoattractants, such as *CCL4*, *CCL20*, *CCL18*, and *CXCL2*, and cytokines such as *IL1B* and *IL6* (Figure S8F). Intriguingly, AT2 cells co-localized strongly with AT1 cells in ILD patients (Figure 4B), but not in non-ILD controls (Figure S8G), suggesting increased differentiation into AT1 cells. monoMacs and act.alvMacs expressed higher levels of epidermal growth factor ligands, such as AREG and EREG (Figure S8F), which could provide the signal for AT2 differentiation.[43] Aberrant trAT cells were strongly co-localized with AT2 and AT1 cells, as well as basal cells and *CTHRC1*-hi MyoF in diseased tissues (Figures 4A–4C). Aberrant trAT and *CTHRC1*-hi MyoF were enriched in c11 and regions of higher fibrotic activity such as

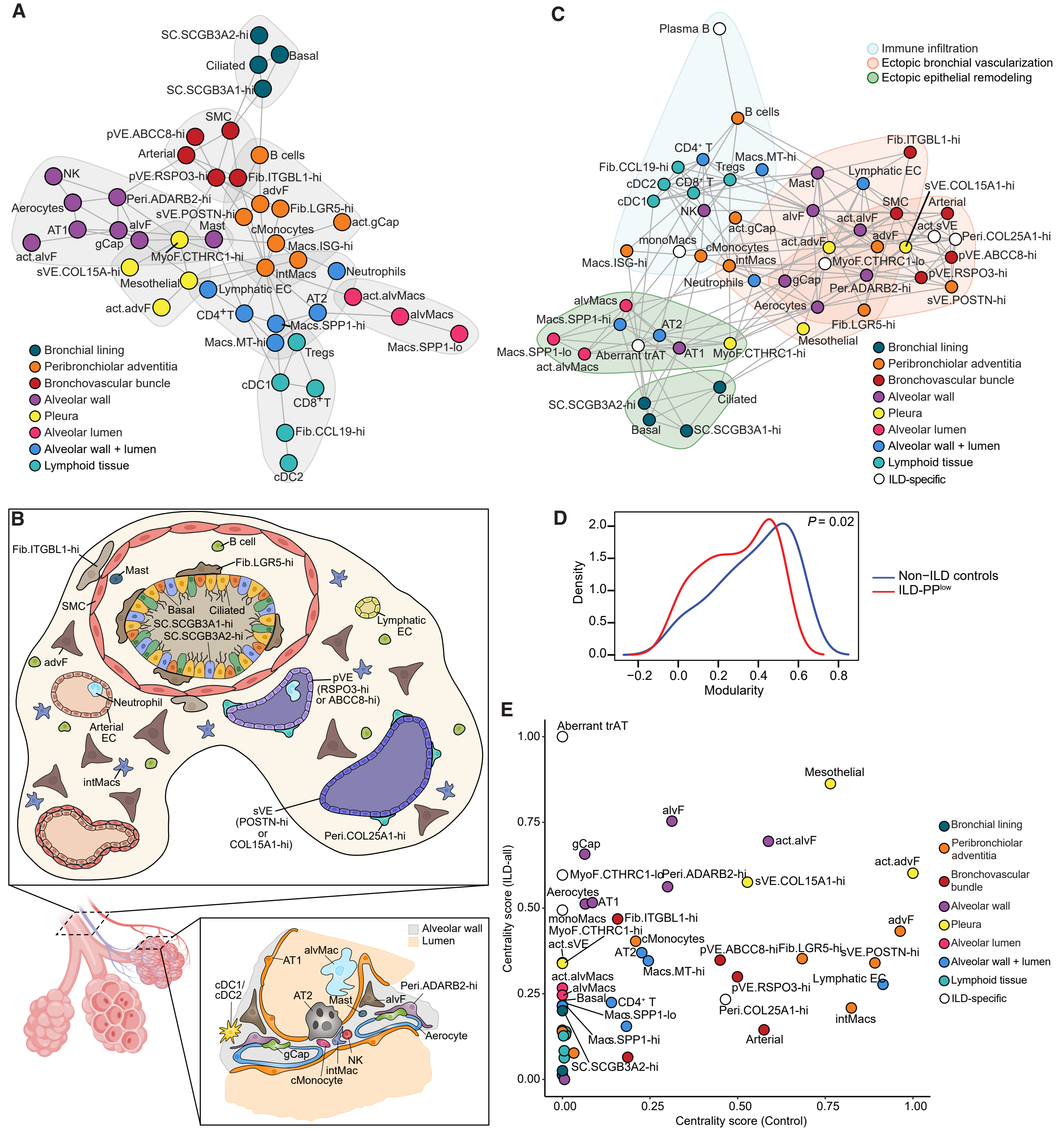

Figure 3. Loss of tissue structure and organization in lung fibrosis

(A) Graph plot showing cell types as nodes based on community detection analysis. Edge between any two nodes indicates that the two cell types are significantly likely to be co-localized in the same spot. The gray shaded areas delineate distinct communities, manually colored based on the known anatomical units of the lung.

(B) Schematics of anatomical localization of cell types in cross-sections of a healthy lung bronchiole (top) and alveolus (bottom right).[39]

(C) Graph plot showing cell types as nodes based on community detection analysis. Nodes are colored according to their associated anatomical unit as in (A); white represents nodes that are not present in the non-ILD control network. Shaded areas delineate distinct communities and are color-coded by the disease process with which they are associated. Edge between any two nodes indicates the two cell types are significantly likely to be co-localized in the same spot.

(D) Distribution of modularity scores on each node shown in (A) and (C), demonstrating loss of modularity in ILD tissues. A two-sided Wilcoxon test was performed to assess for statistical difference between the two distributions.

(E) Scatterplot of eigenvector centrality scores computed for each cell type on non-ILD control and ILD-specific intercellular signaling networks.

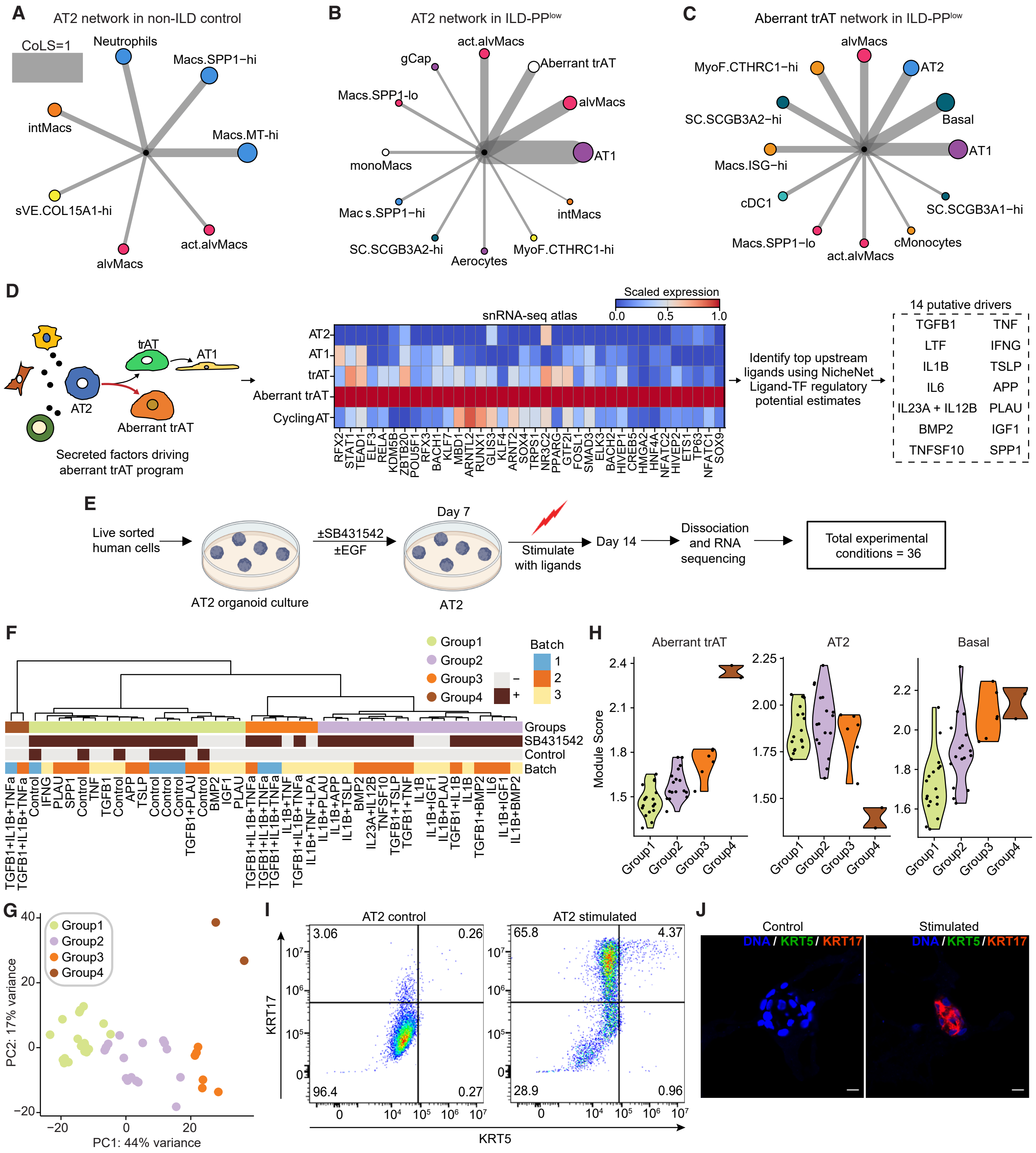

Figure 4. Cellular neighborhoods and drivers of Aberrant trAT cells

(A–C) Cellular neighborhoods of (A) AT2 cells in non-ILD controls, (B) AT2 cells in ILD-PP^{low} tissues, and (C) Aberrant trAT cells in ILD-PP^{low} tissues based on spot colocalization analysis. Edge thickness represents the strength of co-localization (CoLS) normalized to 1. Node colors are harmonized to Figures 3A and 3B.

(D and E) Schematics of (D) computational workflow and selection strategy and (E) experimental workflow for identifying and experimentally validating upstream drivers of Aberrant trAT cells.[44] The heatmap (D, center) depicts the scaled gene expression of TFs significantly enriched in Aberrant trAT cells, and their potential inducing ligands (D, right).

(legend continued on next page)

fibroblastic foci and Parenchyma-P (Figure 2G), linking the presence of Aberrant trAT to *CTHRC1*-hi MyoF.

Cell-extrinsic drivers of Aberrant trAT cells

Next, we hypothesized that secreted factors present in pro-inflammatory environments may skew AT2 differentiation toward the maladaptive Aberrant trAT state. We implemented a computational workflow based on inferred regulon activity of transcription factors (TFs) (STAR Methods; Figure 4D) to prioritize 14 ligands potentially driving the Aberrant trAT gene program. We employed a feeder-free primary human AT2 organoid culture system[45] to screen for the effects of ligands (Figure 4E). Because *CTHRC1*-hi MyoF, alveolar macrophages, and monoMacs colocalized with Aberrant trAT cells (Figure 4C), we prioritized dual combinations with the known pro-fibrotic ligands TGF-β and IL-1β, whose receptors were also expressed by Aberrant trAT cells (Figure S8H). We also tested other parameters, such as the presence of the TGF-β signaling inhibitors, including SB431542, and epidermal growth factor (EGF) in the culture media, yielding a total of 36 unique experimental conditions (Figure 4E) assayed by bulk RNA-seq.

Hierarchical clustering of transcriptome profiles revealed four major groups (Figures 4F and 4G). PCA analysis further revealed that most of the samples were segregated along the first principal component (PC1) axis and that group 4 was conspicuously different from other groups (Figures 4G; S8I). Group 1 consisted of unstimulated controls and individual ligand stimulation, suggesting minimal impact and, based on epithelial cell-type-specific signatures, the lowest proportions of Aberrant trAT cells (Figures 4H; S8J–S8K). Notably, individual stimulation with TGF-β, TNF-α, or IL-1β did not induce the differentiation of Aberrant trAT cells (Figure S8K). Group 2 and 3 samples had modest induction of the Aberrant trAT cell type signature, with group 2 consisting primarily of samples stimulated in dual combination with either IL-1β or TGF-β. In contrast, group 4 exhibited a strong induction of the Aberrant trAT signature and consisted of samples treated with the triplet combination of TGF-β, IL-1β, and TNF-α without SB431542. Notably, group 3 also consisted of samples stimulated with TGF-β, IL-1β, and TNF-α, but with SB431542 present in the media or with TGF-β removed from the combination, suggesting that functional TGF-β signaling was required for the induction of Aberrant trAT cells (Figures 4F and 4H). Immunophenotyping of human AT2 organoids stimulated with this combination demonstrated a strong induction of KRT17 in organoids, with limited expression of the basal marker KRT5, confirming the expansion of a population reminiscent of Aberrant trAT cells (Figures 4I and 4J).

Overall, we observe that the neighborhood of AT2 cells in ILD tissues is highly pro-inflammatory and pro-fibrotic, which in turn provides the necessary stimuli, in the form of secreted factors such as TGF-β, IL-1β, and TNF-α, to skew the normal trajectory of AT2-AT1 differentiation toward the Aberrant trAT state.

Pathogenic spectrum of *CTHRC1*+ MyoF and their relation to the divergent spatial niche

Dysregulated fibroblast activity is a key driver of lung fibrosis, so we examined the heterogeneity of fibroblast cells in the distal lung. We annotated two subsets of MyoF (*CTHRC1*-hi/-lo) that expressed higher levels of ECM remodeling genes than other fibroblasts (Figures 5A; S9A) and were expanded in disease (Figure 1G). Ablation of *Cthrc1*+ fibroblasts in the mouse lung has been shown to attenuate bleomycin-induced lung fibrosis.[17] Our data revealed an increasing gradient of ECM gene expression from the *CTHRC1*-lo to the *CTHRC1*-hi subset (Figure 5A), implying a gradual pro-fibrotic polarization. These MyoF were transcriptionally more similar to alvF than advF (Figures 1C; S2D), and RNA velocity analysis suggested that alvF differentiated into the act.alvFs, *CTHRC1*-lo, and *CCL19*-hi fibroblasts (Figure 5B), consistent with the notion that pro-fibrotic fibroblasts derive from alvF.[17]

Next, we explored whether differences in spatial topography could account for this dynamic polarization. Colocalization analysis of alvFs in healthy and ILD-PP^{low} tissues revealed an apparent transformation of their neighborhood with disease progression. In non-ILD controls, alvFs were localized adjacent to AT1s, gCaps, and Aerocytes (Figure 5C, left); whereas in diseased tissues, these neighborhoods were taken over by non-parenchymal stromal cells such as advFs, pVEs, and SMCs, as well as CD8^{+} T lymphocytes and mast cells (Figure 5C, right). The neighborhood of *CTHRC1*-lo MyoF in diseased tissues was similar to that of alvF (Figure 5D), while the *CTHRC1*-hi MyoF were strongly colocalized with Aberrant trAT, interstitial macrophages, and other alveolar resident cells (Figure 5E), suggesting that the shift in fibroblast polarization from *CTHRC1*-lo to *CTHRC1*-hi state is driven by the surrounding cellular milieu. Accordingly, Aberrant trAT and *CTHRC1*-hi MyoF had the strongest signaling crosstalk score in diseased tissues (Figure S7C). These analyses suggest that the induction of the *CTHRC1*-hi state from alveolar fibroblast or *CTHRC1*-lo MyoF is tightly linked to areas of active epithelial damage where Aberrant trAT cells are present.

NFATC4 is a mechanosensitive regulator of *CTHRC1*-hi myofibroblasts

We next sought to identify the cell-intrinsic mediators involved in the dynamic regulation of the pro-fibrotic *CTHRC1*-hi state. By integrating regulon activity estimates, we prioritized several putative TF regulators that were enriched for activity in *CTHRC1*-hi cells, as well as in spatial communities and fibroblastic foci regions where *CTHRC1*-hi cells are abundant (Figures 5F and 5G; S9B). Using *in vitro*-cultured lung fibroblasts, we first stimulated cells with TGF-β and confirmed that *CTHRC1* was induced, along with several ECM genes, including *COL1A1* and *ACTA2* (Figures 5H; S9C). We next investigated the role of prioritized

(F) Hierarchical clustering of transcriptional profiles obtained from (E), identifying 4 major groups of samples. Each column represents one experimental observation colored by group, condition, and experimental batch.
(G) PCA of the same transcriptional profiles as in (F). Each dot represents one experimental observation colored by group.
(H) Violin plot of groupwise cell-type-specific signature scores computed on transcriptome profiles using cell-type-specific markers from the scRNA-seq atlas.
(I and J) (I) Fluorescence-activated cell sorting (FACS) plot showing gates for KRT5 and KRT17 and (J) immunostaining for KRT5 and KRT17, in control and ligand cocktail TGFβ+TNFα+IL1β-stimulated AT2 organoids. Scale bar, 10μm.

CellPress
OPEN ACCESS

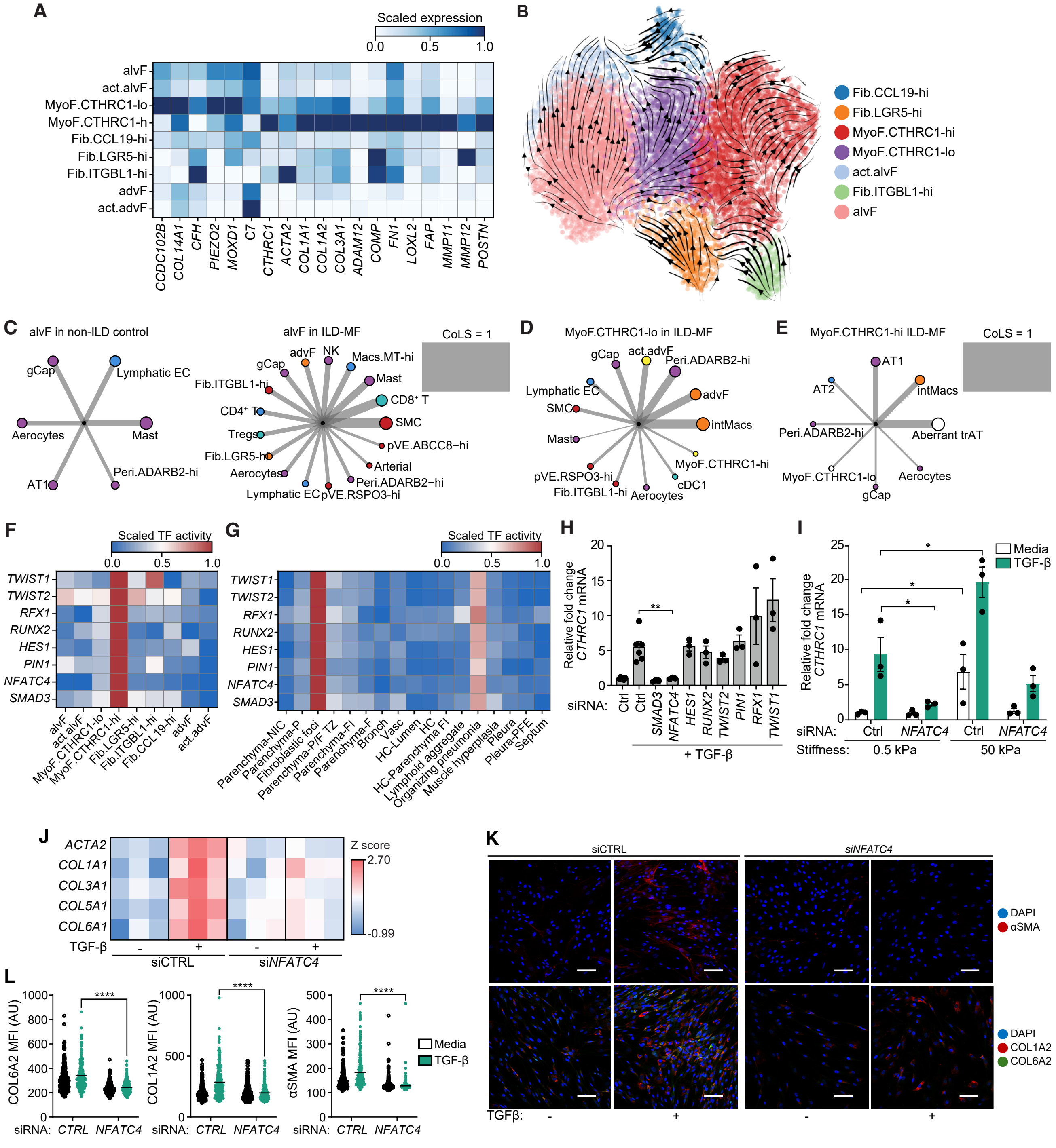

Figure 5. Spatial niche-dependent regulation of *CTHRC1*-hi MyoF

(A) Heatmap of selected genes enriched in MyoF subsets in the scRNA-seq atlas. Differential expression analysis was performed using the Wilcoxon test in each cell type and genes with adjusted $p < 0.05$ were considered to be statistically significant.

(B) UMAP of fibroblast cells embedded with RNA velocity vector fields, suggesting that alvFs differentiate into act.alvFs and MyoF.*CTHRC1*-lo/hi cells. The break in vector fields from *CTHRC1*-lo to *CTHRC1*-hi subsets suggests that the *CTHRC1*-hi clusters are dynamically regulated.

(C–E) Cellular neighborhoods, based on spot colocalization analysis of (C) alveolar fibroblasts (alvF) in non-ILD controls (left) and more fibrotic ILD patient (ILD-MF) samples (right), (D) MyoF.*CTHRC1*-lo subset in ILD-MF samples, and (E) MyoF.*CTHRC1*-hi subset in ILD-MF samples. Edge thickness represents the strength of CoLS normalized to 1. Node colors are harmonized to Figures 3A and 3B.

(legend continued on next page)

TFs by siRNA-mediated knockdown and found that, aside from *SMAD3*, which is involved in TGF-β signaling, only inhibition of *NFATC4* consistently attenuated the induction of pathogenic ECM gene programs (Figures 5H; S9C and S9D). *NFATC4* is a TF expressed in non-immune tissues, functions as a sensor of Ca^{2+} flux in the cytoplasm,[46] and translocates to the nucleus after dephosphorylation by calcineurin.

CTHRC1-hi cells were enriched in the Parenchyma-P and fibroblastic foci (Figure 2F), which are known to exhibit increased matrix stiffness relative to the normal parenchyma.[47] Given that fibroblast activation is sensitive to ECM matrix rigidity[48] and Ca^{2+} signaling is a key player in mechanotransduction,[49] we explored its relationship to *NFATC4* and TGF-β activation. Specifically, we utilized both healthy and IPF patient-derived fibroblasts that were cultured in a rigid (50 kPa) or soft (0.5 kPa) substrate (Figures 5I; S9E and S9F). Healthy fibroblasts in the rigid substrate had significantly higher levels of *CTHRC1* even without any stimulation (Figure 5I), underscoring the effect of matrix stiffness on fibroblast activation. Stimulation with TGF-β further increased the expression levels of *CTHRC1* in the rigid substrate (Figure 5I). In both the presence and absence of TGF-β, knockdown of *NFATC4* abrogated the induction of *CTHRC1* in rigid substrate, demonstrating that *NFATC4* is a mechanosensitive regulator of *CTHRC1* (Figure 5I). Interestingly, *NFATC4* transcript levels were downregulated in the stiff matrix (Figure S9E), suggesting that transcriptional regulation of *NFATC4* itself is mechanosensitive, potentially driven by negative feedback regulation. To extend our analyses beyond *CTHRC1*, we next measured the expression of *ACTA2* and multiple collagen genes and found that *NFATC4* knockdown abrogated the induction of these genes by TGF-β (Figure 5J). Finally, we directly assessed the protein levels of smooth muscle actin and collagens by immunofluorescence and again found that *NFATC4* knockdown reduced the levels of deposited collagens and actin filaments (Figures 5K–5L). In conclusion, we have identified *NFATC4* as a mechanosensitive regulator of a pro-fibrotic gene program in lung fibroblasts and validated its targeting as a potential strategy to reduce collagen deposition.

DISCUSSION

Repetitive injury to the alveolar epithelium is a major driver of lung fibrosis,[9] with genetic risk factors for IPF implicating genes involved in epithelial barrier maintenance and function.[50,51] Epithelial repair requires AT2 cell differentiation through states involving a transitional AT2-AT1 population. Here, using snRNA-seq, we identify both normal and aberrant ILD-associated trAT cells, potentially because of the higher capture efficiency of elongated cells by snRNA-seq protocols. Intermediate epithelial cells transcriptionally similar to Aberrant trATs have also been observed in acute lung injury in both humans[52] and mice.[18–21] A previous study modeling AT2 organoids co-cultured with mesenchymal cells reported that AT2 cells can transdifferentiate into KRT5+KRT17+ basal cells over a span of 21 days, with intermediate populations that resemble Aberrant trAT cells appearing at day 7, along with significant induction of KRT5+ basal cells.[53] In contrast, we did not observe the induction of KRT5+ KRT17+ basal cells at day 7 in our feeder-free AT2 organoids stimulated with TGF-β, IL-1β, and TNF-α. However, we observed basal cells localized in the neighborhood of Aberrant trAT cells in diseased tissues. Moreover, a previous study suggested that *in vivo* IL-1β signaling is required for differentiation of AT2 cells into the transient, injury-induced Aberrant trAT-like cells.[18] Here, in isolated *in vitro* settings, neither IL-1β, TGF-β, nor TNF-α signaling alone was sufficient to induce the Aberrant trAT state. We also noted that in our atlases, Aberrant trAT cells are enriched for markers of cellular senescence, which has been linked to cellular plasticity and dedifferentiation in the context of tissue regeneration,[54] but was not directly examined in our system and will require further investigation. We find that the neighborhood of AT2 cells in damaged tissues is highly pro-inflammatory and myeloid-rich, which drives AT2 cell differentiation. However, the pro-inflammatory environment may also induce stromal cells, such as alvFs, to differentiate into pro-fibrotic MyoF, which in turn may hyperactivate TGF-β signaling and skew the trajectory of AT2-trAT-AT1 differentiation toward the Aberrant trAT state, in conjunction with IL-1β and TNF-α secreted by the myeloid cells. Notably, although chronic inflammation, including the development of lymphoid aggregates, is not generally considered a hallmark of IPF,[55] multiple studies have found these structures to be enriched in this disease.[25,56]

Using lineage tracing, Tsukui et al. described the emergence of inflammatory fibroblasts early in response to injury, followed by fibrotic fibroblasts.[17] Corroborating these findings, we find that human alvFs differentiate into activated fibroblasts with an inflammatory expression profile and into *CTHRC1*-hi MyoF with a pro-fibrotic expression profile. While Tsukui et al. observed the induction of serum amyloid A3 (*Saa3*), lipocalin 2 (*Lcn2*), and *Spp1* in injury-responsive fibroblasts in mice,[17] we did not detect expression of these genes in our dataset, highlighting potential organismal differences. Expanding on this, we find that MyoFs in the distal lung exist on a spectrum of pro-fibrotic activity, and that the shift from *CTHRC1-lo* to *CTHRC1-hi* states may be linked to their spatial proximity to Aberrant trAT cells. We further identify

(F and G) Heatmaps of selected TFs enriched in estimated activity in (F) MyoF.*CTHRC1*-hi subsets in the scRNA-seq data and (G) annotated histopathological regions in the spatial transcriptomics data.

(H and I) Bar plots showing relative levels of *CTHRC1* mRNA after TGFβ stimulation of (H) wild-type control (Ctrl) and selected TF knockdown fibroblasts and (I) healthy Ctrl and *NFATC4*-knockdown fibroblasts cultured in soft (0.5 kPa) or rigid (50 kPa) substrates. *$p < 0.05$, **$p < 0.01$. Error bars represent SEM. Unpaired Student's *t* test (two-sided); $n = 3$.

(J) Quantification of mRNA levels for ECM-related genes, measured by qPCR, after TGFβ stimulation with control (Ctrl) and *NFATC4*-knockdown in healthy fibroblasts cultured in soft (0.5 kPa) substrates.

(K) Immunostaining for alpha smooth muscle actin (aSMA) (red) or COL1A2 (red) with COL6A2 (green) after TGFβ stimulation with control (Ctrl) and *NFATC4*-knockdown in healthy fibroblasts cultured in soft (0.5 kPa) substrates. Scale bar, 100 μm.

(L) Quantification of immunofluorescence of ECM-related genes shown in (K). MFI (AU), mean fluorescence intensity expressed in arbitrary units. Two-way ANOVA with Tukey's multiple comparisons was performed. ****$p < 0.0001$.

CellPress
OPEN ACCESS

a mechanosensitive TF, *NFATC4*, as a regulator of myofibroblast differentiation. *NFATC4* is selectively enriched for expression in alveolar fibroblast subsets, making it a potential therapeutic target. Interestingly, a clinical comparative study demonstrated that a combination of prednisolone with tacrolimus, an NFAT inhibitor used as an immunosuppressant, reduced ILD progression and improved lung function,[57,58] which our data suggest may reflect activity beyond the immune system alone.

The expansion of venous endothelial cells in the fibrotic parenchyma, which exhibited less fibrotic activity, is supported by a previous observation suggesting an inverse correlation between vascular density and degree of fibrosis.[59] Morphometric analysis of vasculature[60] suggests increased vessel diameter, decreased intervascular distance, and neoangiogenesis in alveolar fibroelastosis lungs. Our observations elaborate on previous findings and suggest that the fibrotic parenchyma could be a site of ectopic neovascularization and smooth muscle proliferation. Airway smooth muscle hypertrophy is known to occur in usual interstitial pneumonia (UIP) pattern of fibrosis.[61] SMCs can penetrate the alveolar walls bordering the alveolar ducts,[62] and the density of smooth muscle relative to airway diameter is much higher in the terminal bronchioles.[62,63] Remnants of alveolar septa in the fibrotic parenchyma may provide the substrate for smooth muscle migration and proliferation. Smooth muscle migration has also been observed during vascular development in response to injury,[64] which suggests that neovascularization of fibrotic parenchyma might provide the necessary signals. However, it is possible that SMC hypertrophy could be a metaplastic phenomenon associated with fibrosis and derived from other sources.

In summary, in this study we characterize the transcriptomic landscape of fibrotic ILD and create a high-resolution molecular and cellular portrait of the disease. Our in-depth analysis also provides a framework for integrating disease histopathology with spatial transcriptomics to understand the pathogenesis of lung fibrosis.

Limitations of the study

Here, we leveraged human lung tissue from ILD and non-ILD patients to identify cellular and molecular changes associated with fibrosis. Because most samples were obtained in the context of resections and transplants, one important limitation is a bias toward advanced and end-state disease states. Histological assessment enabled us to separate more- and less-fibrotic areas, but further studies using other, less-invasive, sampling approaches earlier in disease would be necessary to fully understand ILD progression across both time and space. Our spatial assessments rely on transcript capture across large spots (50 μm), which were then computationally deconvoluted, introducing a dependence on the reference atlas. Novel approaches with cellular or subcellular resolution, combined with protein stainings, will enable reference-free cellular annotations and are likely to further refine our understanding of direct cell-cell and cell-matrix interactions. Finally, our *in vitro* models highlighted the role of specific cytokines and TFs in reductionist systems, but complementary validations in animal systems that encompass the entire cellular context of ILD will be necessary to understand how these drivers interact with the complex cellular and molecular perturbations that occur in the fibrotic niche.

RESOURCE AVAILABILITY

Lead contact

Further information and requests for resources and reagents should be directed to and will be fulfilled by the lead contact, Ramnik J. Xavier (xavier@molbio.mgh.harvard.edu).

Materials availability

This study did not generate any novel reagents. All materials are commercially available.

Data and code availability

- Raw sequencing files have been deposited in dbGAP under accession code dbGAP: phs004408.v1.p1 and in SRA as SRA: PRJNA1390708. Processed sequencing data as well as raw histology files have been made available through the Broad's Single Cell Portal (https://singlecell.broadinstitute.org/single_cell) SCP2879. These data are publicly available as of the date of publication.
- This paper does not report original code.
- Any additional information required to reanalyze the data reported in this paper is available from the lead contact upon request.

ACKNOWLEDGMENTS

We thank participating patients and research staff at the Center for the Study of Inflammatory Bowel Disease. The authors also thank Luke Besse and Eric Chen for project and data management, Helena Lau for sample management and metadata collection, and Cristin McCabe and the Broad Genomics Platform for help with sequencing data generation. We thank Susana Guimarães and Margarida Saraiva for their role at managing the HSJ, Portuguese biobank. We thank Heather Kang and Stephanie Aldrich for editorial assistance with the manuscript and figures. We thank Jehan Alladina for discussions and insights. This study was supported by funding from the Manton Foundation and the Klarman Cell Observatory to R.J.X.; from the National Institutes of Health to R.J.X (U19 AI110495), J.D. (U01 HL175384), and B.D.M. (R01 HL157384 and U01 HL175384); and from the German Research Foundation to I.G. (Walter-Benjamin-Program fellowship 490745655). H.N.B., R.F.S., and A.C. were supported by the Fundação para a Ciência e a Tecnologia (FCT) (PTDC/MEC-RES/0158/2020 and PTDC/MED-OUT/1112/2021).

AUTHOR CONTRIBUTIONS

A.J., J.D., B.D.M., and R.J.X. conceived and supervised this study. A.J. wrote the manuscript with contributions from T.K., V.P., J.D., and B.D.M. T.K. supervised and conducted all organoid experiments. V.P. carried out all fibroblast experiments. T.R.S. conducted all organoid experiments. A.S. and C.L. assisted with spatial transcriptomics profiling. L.A.-Z., T.M.D., and S.P.M. assisted with all 10× sequencing data generation. S.T., K.S., L.P.H., and K.E.B. assisted with tissue collection, sample management, and metadata collection at MGH. A.J. performed the histological annotations for spatial transcriptomics with guidance from Y.P.H. and L.P.H. I.K.G. performed fibroblast cultures. R.K. and D.B.G. contributed intellectual expertise to writing the manuscript. H.N.B., R.F.S., and A.C. supervised and conducted sample collection at HSJ, Portugal.

DECLARATION OF INTERESTS

R.J.X. is board director at MoonLake Immunotherapeutics, co-founder of Convergence Bio, consultant to Nestlé, and a member of the advisory boards for Magnet Biomedicine and Arena Bioworks; B.D.M. has received sponsored research awards from Sanofi and Regeneron and has served on advisory boards for Sanofi, Regeneron, and Apogee; J.D. is a member

of Biorender's scientific advisory board; these organizations had no role in this study.

STAR★METHODS

Detailed methods are provided in the online version of this paper and include the following:

- KEY RESOURCES TABLE
- EXPERIMENTAL MODEL AND STUDY PARTICIPANT DETAILS
- METHOD DETAILS
  - Nuclei isolation and snRNA-seq
  - Sample preparation for Visium profiling
  - Histopathology annotations
  - AT2 cell isolation
  - AT2 cell culture and cryopreservation
  - AT2 organoids ligand screening
  - Transcriptome sequencing using SMART-seq2
  - Detection of KRT17+KRT5- AT2 in organoids
  - Human lung fibroblasts culture
  - siRNA knockdown in primary lung fibroblasts
  - RNA isolation and quantitative RT-PCR
  - Tissue culture plate stiffness assay
  - Immunofluorescence of pro-fibrotic markers
- QUANTIFICATION AND STATISTICAL ANALYSIS
  - snRNA-seq data pre-processing
  - Demultiplexing of pooled samples
  - snRNA-seq atlas integration and annotation
  - Generating the integratedILD scRNA-seq atlas
  - Mapping of snRNA-seq and scRNA-seq atlases
  - Cell composition analysis
  - Community composition analysis
  - Visium data pre-processing
  - Visium data from published studies
  - Spatial transcriptomics analysis and community identification
  - Estimating cell count per Visium spot
  - Integration of sc/snRNA-seq and spatial transcriptomics
  - Cell-type enrichment in spatial communities and histopathological groups
  - Cell communication network analysis
  - Co-localization analysis
  - Network analysis on CoLS estimates
  - RNA velocity analysis
  - Identification of putative driver ligands of the Aberrant trAT gene program
  - Analysis of bulk RNA sequencing of AT2 organoids
  - Selection of MyoF.CTHRC1-hi specific TFs

SUPPLEMENTAL INFORMATION

Supplemental information can be found online at https://doi.org/10.1016/j.xgen.2025.101066.

Received: March 3, 2025
Revised: August 6, 2025
Accepted: October 21, 2025
Published: January 22, 2026

REFERENCES

1. Henderson, N.C., Rieder, F., and Wynn, T.A. (2020). Fibrosis: from mechanisms to medicines. Nature *587*, 555–566.
2. Rockey, D.C., Bell, P.D., and Hill, J.A. (2015). Fibrosis–a common pathway to organ injury and failure. N. Engl. J. Med. *372*, 1138–1149.
3. Wynn, T.A., and Ramalingam, T.R. (2012). Mechanisms of fibrosis: therapeutic translation for fibrotic disease. Nat. Med. *18*, 1028–1040.
4. Nakata, T., Li, C., Mayassi, T., Lin, H., Ghosh, K., Segerstolpe, Å., Diamond, E.L., Herbst, P., Biancalani, T., Gaddam, S., et al. (2023). Genetic vulnerability to Crohn's disease reveals a spatially resolved epithelial restitution program. Sci. Transl. Med. *15*, eadg5252.
5. Stappenbeck, T.S., and Miyoshi, H. (2009). The role of stromal stem cells in tissue regeneration and wound repair. Science *324*, 1666–1669.
6. Eming, S.A., Martin, P., and Tomic-Canic, M. (2014). Wound repair and regeneration: mechanisms, signaling, and translation. Sci. Transl. Med. *6*, 265sr6.
7. Correa-Gallegos, D., Ye, H., Dasgupta, B., Sardogan, A., Kadri, S., Kandi, R., Dai, R., Lin, Y., Kopplin, R., Shenai, D.S., et al. (2023). CD201^{+} fascia progenitors choreograph injury repair. Nature *623*, 792–802.
8. Aggarwal, S., Wang, Z., Rincon Fernandez Pacheco, D., Rinaldi, A., Rajewski, A., Callemeyn, J., Van Loon, E., Lamarthée, B., Covarrubias, A.E., Hou, J., et al. (2024). SOX9 switch links regeneration to fibrosis at the single-cell level in mammalian kidneys. Science *383*, eadd6371.
9. Katzen, J., and Beers, M.F. (2020). Contributions of alveolar epithelial cell quality control to pulmonary fibrosis. J. Clin. Investig. *130*, 5088–5099.
10. Lederer, D.J., and Martinez, F.J. (2018). Idiopathic Pulmonary Fibrosis. N. Engl. J. Med. *378*, 1811–1823.
11. Reyfman, P.A., Walter, J.M., Joshi, N., Anekalla, K.R., McQuattie-Pimentel, A.C., Chiu, S., Fernandez, R., Akbarpour, M., Chen, C.-I., Ren, Z., et al. (2019). Single-Cell Transcriptomic Analysis of Human Lung Provides Insights into the Pathobiology of Pulmonary Fibrosis. Am. J. Respir. Crit. Care Med. *199*, 1517–1536.
12. Adams, T.S., Schupp, J.C., Poli, S., Ayaub, E.A., Neumark, N., Ahangari, F., Chu, S.G., Raby, B.A., Deluliis, G., Januszyk, M., et al. (2020). Single-cell RNA-seq reveals ectopic and aberrant lung-resident cell populations in idiopathic pulmonary fibrosis. Sci. Adv. *6*, eaba1983.
13. Habermann, A.C., Gutierrez, A.J., Bui, L.T., Yahn, S.L., Winters, N.I., Calvi, C.L., Peter, L., Chung, M.-I., Taylor, C.J., Jetter, C., et al. (2020). Single-cell RNA sequencing reveals profibrotic roles of distinct epithelial and mesenchymal lineages in pulmonary fibrosis. Sci. Adv. *6*, eaba1972.
14. Carraro, G., Mulay, A., Yao, C., Mizuno, T., Konda, B., Petrov, M., Lafkas, D., Arron, J.R., Hogaboam, C.M., Chen, P., et al. (2020). Single-Cell Reconstruction of Human Basal Cell Diversity in Normal and Idiopathic Pulmonary Fibrosis Lungs. Am. J. Respir. Crit. Care Med. *202*, 1540–1550.
15. DePianto, D.J., Heiden, J.A.V., Morshead, K.B., Sun, K.-H., Modrusan, Z., Teng, G., Wolters, P.J., and Arron, J.R. (2021). Molecular mapping of interstitial lung disease reveals a phenotypically distinct senescent basal epithelial cell population. JCI Insight *6*, e143626. https://doi.org/10.1172/jci.insight.143626.
16. Tsukui, T., Sun, K.-H., Wetter, J.B., Wilson-Kanamori, J.R., Hazelwood, L.A., Henderson, N.C., Adams, T.S., Schupp, J.C., Poli, S.D., Rosas, I.O., et al. (2020). Collagen-producing lung cell atlas identifies multiple subsets with distinct localization and relevance to fibrosis. Nat. Commun. *11*, 1920.
17. Tsukui, T., Wolters, P.J., and Sheppard, D. (2024). Alveolar fibroblast lineage orchestrates lung inflammation and fibrosis. Nature *631*, 627–634.
18. Choi, J., Park, J.-E., Tsagkogeorga, G., Yanagita, M., Koo, B.-K., Han, N., and Lee, J.-H. (2020). Inflammatory Signals Induce AT2 Cell-Derived Damage-Associated Transient Progenitors that Mediate Alveolar Regeneration. Cell Stem Cell *27*, 366–382.e7.
19. Wang, F., Ting, C., Riemondy, K.A., Douglas, M., Foster, K., Patel, N., Kaku, N., Linsalata, A., Nemzek, J., Varisco, B.M., et al. (2023). Regulation of epithelial transitional states in murine and human pulmonary fibrosis. J. Clin. Investig. *133*, e165612. https://doi.org/10.1172/JCI165612.
20. Strunz, M., Simon, L.M., Ansari, M., Kathiriya, J.J., Angelidis, I., Mayr, C.H., Tsidiridis, G., Lange, M., Mattner, L.F., Yee, M., et al. (2020). Alveolar regeneration through a Krt8+ transitional stem cell state that persists in human lung fibrosis. Nat. Commun. *11*, 3559.
21. Kobayashi, Y., Tata, A., Konkimalla, A., Katsura, H., Lee, R.F., Ou, J., Banovich, N.E., Kropski, J.A., and Tata, P.R. (2020). Persistence of a

regeneration-associated, transitional alveolar epithelial cell state in pulmonary fibrosis. Nat. Cell Biol. *22*, 934–946.

22. Rao, A., Barkley, D., França, G.S., and Yanai, I. (2021). Exploring tissue architecture using spatial transcriptomics. Nature *596*, 211–220.
23. Mayr, C.H., Santacruz, D., Jarosch, S., Bleck, M., Dalton, J., McNabola, A., Lempp, C., Neubert, L., Rath, B., Kamp, J.C., et al. (2024). Spatial transcriptomic characterization of pathologic niches in IPF. Sci. Adv. *10*, eadl5473.
24. Franzén, L., Olsson Lindvall, M., Hühn, M., Ptasinski, V., Setyo, L., Keith, B.P., Collin, A., Oag, S., Volckaert, T., Borde, A., et al. (2024). Mapping spatially resolved transcriptomes in human and mouse pulmonary fibrosis. Nat. Genet. *56*, 1725–1736.
25. Vannan, A., Lyu, R., Williams, A.L., Negretti, N.M., Mee, E.D., Hirsh, J., Hirsh, S., Hadad, N., Nichols, D.S., Calvi, C.L., et al. (2025). Spatial transcriptomics identifies molecular niche dysregulation associated with distal lung remodeling in pulmonary fibrosis. Nat. Genet. *57*, 647–658.
26. Madissoon, E., Oliver, A.J., Kleshchevnikov, V., Wilbrey-Clark, A., Polanski, K., Richoz, N., Ribeiro Orsi, A., Mamanova, L., Bolt, L., Elmentaite, R., et al. (2023). A spatially resolved atlas of the human lung characterizes a gland-associated immune niche. Nat. Genet. *55*, 66–77.
27. Eraslan, G., Drokhlyansky, E., Anand, S., Fiskin, E., Subramanian, A., Slyper, M., Wang, J., Van Wittenberghe, N., Rouhana, J.M., Waldman, J., et al. (2022). Single-nucleus cross-tissue molecular reference maps toward understanding disease gene function. Science *376*, eabl4290.
28. Kadur Lakshminarasimha Murthy, P., Sontake, V., Tata, A., Kobayashi, Y., Macadlo, L., Okuda, K., Conchola, A.S., Nakano, S., Gregory, S., Miller, L.A., et al. (2022). Human distal lung maps and lineage hierarchies reveal a bipotent progenitor. Nature *604*, 111–119.
29. Lang, N.J., Gote-Schniering, J., Porras-Gonzalez, D., Yang, L., De Sadeleer, L.J., Jentzsch, R.C., Shitov, V.A., Zhou, S., Ansari, M., Agami, A., et al. (2023). Ex vivo tissue perturbations coupled to single-cell RNA-seq reveal multilineage cell circuit dynamics in human lung fibrogenesis. Sci. Transl. Med. *15*, eadh0908.
30. Saul, D., Kosinsky, R.L., Atkinson, E.J., Doolittle, M.L., Zhang, X., LeBrasseur, N.K., Pignolo, R.J., Robbins, P.D., Niedernhofer, L.J., Ikeno, Y., et al. (2022). A new gene set identifies senescent cells and predicts senescence-associated pathways across tissues. Nat. Commun. *13*, 4827.
31. Gillich, A., Zhang, F., Farmer, C.G., Travaglini, K.J., Tan, S.Y., Gu, M., Zhou, B., Feinstein, J.A., Krasnow, M.A., and Metzger, R.J. (2020). Capillary cell-type specialization in the alveolus. Nature *586*, 785–789.
32. Remmerie, A., Martens, L., Thoné, T., Castoldi, A., Seurinck, R., Pavie, B., Roels, J., Vanneste, B., De Prijck, S., Vanhockerhout, M., et al. (2020). Osteopontin Expression Identifies a Subset of Recruited Macrophages Distinct from Kupffer Cells in the Fatty Liver. Immunity *53*, 641–657.e14.
33. Ramachandran, P., Dobie, R., Wilson-Kanamori, J.R., Dora, E.F., Henderson, B.E.P., Luu, N.T., Portman, J.R., Matchett, K.P., Brice, M., Marwick, J.A., et al. (2019). Resolving the fibrotic niche of human liver cirrhosis at single-cell level. Nature *575*, 512–518.
34. Ouyang, J.F., Mishra, K., Xie, Y., Park, H., Huang, K.Y., Petretto, E., and Behmoaras, J. (2023). Systems level identification of a matrisome-associated macrophage polarisation state in multi-organ fibrosis. eLife *12*, e85530. https://doi.org/10.7554/eLife.85530.
35. Morse, C., Tabib, T., Sembrat, J., Buschur, K.L., Bittar, H.T., Valenzi, E., Jiang, Y., Kass, D.J., Gibson, K., Chen, W., et al. (2019). Proliferating SPP1/MERTK-expressing macrophages in idiopathic pulmonary fibrosis. Eur. Respir. J. *54*, 1802441. https://doi.org/10.1183/13993003.02441-2018.
36. Joshi, N., Watanabe, S., Verma, R., Jablonski, R.P., Chen, C.-I., Cheresh, P., Markov, N.S., Reyfman, P.A., McQuattie-Pimentel, A.C., Sichizya, L., et al. (2020). A spatially restricted fibrotic niche in pulmonary fibrosis is sustained by M-CSF/M-CSFR signalling in monocyte-derived alveolar macrophages. Eur. Respir. J. *55*, 1900646. https://doi.org/10.1183/13993003.00646-2019.
37. Misharin, A.V., Morales-Nebreda, L., Reyfman, P.A., Cuda, C.M., Walter, J.M., McQuattie-Pimentel, A.C., Chen, C.-I., Anekalla, K.R., Joshi, N., Williams, K.J.N., et al. (2017). Monocyte-derived alveolar macrophages drive lung fibrosis and persist in the lung over the life span. J. Exp. Med. *214*, 2387–2404.
38. Hickey, J.W., Becker, W.R., Nevins, S.A., Horning, A., Perez, A.E., Zhu, C., Zhu, B., Wei, B., Chiu, R., Chen, D.C., et al. (2023). Organization of the human intestine at single-cell resolution. Nature *619*, 572–584.
39. Kang, H. (2025). Created in BioRender. https://BioRender.com/mfiyw9u.
40. Armingol, E., Officer, A., Harismendy, O., and Lewis, N.E. (2021). Deciphering cell-cell interactions and communication from gene expression. Nat. Rev. Genet. *22*, 71–88.
41. Karaiskos, N., Wahle, P., Alles, J., Boltengagen, A., Ayoub, S., Kipar, C., Kocks, C., Rajewsky, N., and Zinzen, R.P. (2017). The *Drosophila* embryo at single-cell transcriptome resolution. Science *358*, 194–199.
42. Longo, S.K., Guo, M.G., Ji, A.L., and Khavari, P.A. (2021). Integrating single-cell and spatial transcriptomics to elucidate intercellular tissue dynamics. Nat. Rev. Genet. *22*, 627–644.
43. Kaiser, K.A., Loffredo, L.F., Santos-Alexis, K.d.L., Ringham, O.R., and Arpaia, N. (2023). Regulation of the alveolar regenerative niche by amphiregulin-producing regulatory T cells. J. Exp. Med. *220*, e20221462. https://doi.org/10.1084/jem.20221462.
44. Kang, H. (2025). Created in BioRender. https://BioRender.com/se5mgki.
45. Konishi, S., Tata, A., and Tata, P.R. (2022). Defined conditions for long-term expansion of murine and human alveolar epithelial stem cells in three-dimensional cultures. STAR Protoc. *3*, 101447.
46. Yang, T., Davis, R.J., and Chow, C.-W. (2001). Requirement of Two NFATc4 Transactivation Domains for CBP Potentiation. J. Biol. Chem. *276*, 39569–39576.
47. Guo, T., He, C., Venado, A., and Zhou, Y. (2022). Extracellular Matrix Stiffness in Lung Health and Disease. Compr. Physiol. *12*, 3523–3558.
48. Liu, F., Mih, J.D., Shea, B.S., Kho, A.T., Sharif, A.S., Tager, A.M., and Tschumperlin, D.J. (2010). Feedback amplification of fibrosis through matrix stiffening and COX-2 suppression. J. Cell Biol. *190*, 693–706.
49. Rahaman, S.O., Grove, L.M., Paruchuri, S., Southern, B.D., Abraham, S., Niese, K.A., Scheraga, R.G., Ghosh, S., Thodeti, C.K., Zhang, D.X., et al. (2014). TRPV4 mediates myofibroblast differentiation and pulmonary fibrosis in mice. J. Clin. Investig. *124*, 5225–5238.
50. Partanen, J.J., Häppölä, P., Zhou, W., Lehisto, A.A., Ainola, M., Sutinen, E., Allen, R.J., Stockwell, A.D., Leavy, O.C., Oldham, J.M., et al. (2022). Leveraging global multi-ancestry meta-analysis in the study of idiopathic pulmonary fibrosis genetics. Cell Genom. *2*, 100181.
51. Allen, R.J., Guillen-Guio, B., Oldham, J.M., Ma, S.-F., Dressen, A., Paynton, M.L., Kraven, L.M., Obeidat, M., Li, X., Ng, M., et al. (2020). Genome-Wide Association Study of Susceptibility to Idiopathic Pulmonary Fibrosis. Am. J. Respir. Crit. Care Med. *201*, 564–574.
52. Delorey, T.M., Ziegler, C.G.K., Heimberg, G., Normand, R., Yang, Y., Segerstolpe, Å., Abbondanza, D., Fleming, S.J., Subramanian, A., Montoro, D.T., et al. (2021). COVID-19 tissue atlases reveal SARS-CoV-2 pathology and cellular targets. Nature *595*, 107–113.
53. Kathiriya, J.J., Wang, C., Zhou, M., Brumwell, A., Cassandras, M., Le Saux, C.J., Cohen, M., Alysandratos, K.-D., Wang, B., Wolters, P., et al. (2022). Human alveolar type 2 epithelium transdifferentiates into metaplastic KRT5^{+} basal cells. Nat. Cell Biol. *24*, 10–23.
54. Ring, N.A.R., Valdivieso, K., Grillari, J., Redl, H., and Ogrodnik, M. (2022). The role of senescence in cellular plasticity: Lessons from regeneration and development and implications for age-related diseases. Dev. Cell *57*, 1083–1101.
55. Raghu, G., Remy-Jardin, M., Richeldi, L., Thomson, C.C., Inoue, Y., Johkoh, T., Kreuter, M., Lynch, D.A., Maher, T.M., Martinez, F.J., et al. (2022). Idiopathic pulmonary fibrosis (an update) and Progressive pulmonary fibrosis in adults: An official ATS/ERS/JRS/ALAT clinical practice guideline. Am. J. Respir. Crit. Care Med. *205*, e18–e47.

56. Cocconcelli, E., Balestro, E., Turato, G., Fiorentù, G., Bazzan, E., Biondini, D., Tinè, M., Bernardinello, N., Pezzuto, F., Baraldo, S., et al. (2024). Tertiary lymphoid structures and B-cell infiltration are IPF features with functional consequences. Front. Immunol. *15*, 1437767.

57. Huang, D., Li, Y., and Liu, Y. (2023). Tacrolimus and the Treatment of Pulmonary Fibrosis. Am. J. Respir. Crit. Care Med. *208*, 1241–1242.

58. Kiboshi, T., Kotani, T., Konma, J., Makino, H., Matsuda, S., Suzuka, T., Wada, Y., Shiba, H., Hata, K., Shoda, T., and Takeuchi, T. (2022). Comparison of therapeutic effects of combination therapy with prednisolone and tacrolimus or azathioprine on progressive interstitial pneumonia with systemic sclerosis. Mod. Rheumatol. *32*, 358–364.

59. Ebina, M., Shimizukawa, M., Shibata, N., Kimura, Y., Suzuki, T., Endo, M., Sasano, H., Kondo, T., and Nukiwa, T. (2004). Heterogeneous increase in CD34-positive alveolar capillaries in idiopathic pulmonary fibrosis. Am. J. Respir. Crit. Care Med. *169*, 1203–1208.

60. Ackermann, M., Stark, H., Neubert, L., Schubert, S., Borchert, P., Linz, F., Wagner, W.L., Stiller, W., Wielpütz, M., Hoefer, A., et al. (2020). Morphomolecular motifs of pulmonary neoangiogenesis in interstitial lung diseases. Eur. Respir. J. *55*, 1900933. https://doi.org/10.1183/13993003.00933-2019.

61. Smith, M., Dalurzo, M., Panse, P., Parish, J., and Leslie, K. (2013). Usual interstitial pneumonia-pattern fibrosis in surgical lung biopsies. Clinical, radiological and histopathological clues to aetiology. J. Clin. Pathol. *66*, 896–903.

62. Cieri, R.L. (2019). Pulmonary Smooth Muscle in Vertebrates: A Comparative Review of Structure and Function. Integr. Comp. Biol. *59*, 10–28.

63. Ebina, M., Yaegashi, H., Takahashi, T., Motomiya, M., and Tanemura, M. (1990). Distribution of smooth muscles along the bronchial tree. A morphometric study of ordinary autopsy lungs. Am. Rev. Respir. Dis. *141*, 1322–1326.

64. Gerthoffer, W.T. (2007). Mechanisms of vascular smooth muscle cell migration. Circ. Res. *100*, 607–621.

65. Li, B., Gould, J., Yang, Y., Sarkizova, S., Tabaka, M., Ashenberg, O., Rosen, Y., Slyper, M., Kowalczyk, M.S., Villani, A.-C., et al. (2020). Cumulus provides cloud-based data analysis for large-scale single-cell and single-nucleus RNA-seq. Nat. Methods *17*, 793–798.

66. Fleming, S.J., Chaffin, M.D., Arduini, A., Akkad, A.-D., Banks, E., Marioni, J.C., Philippakis, A.A., Ellinor, P.T., and Babadi, M. (2023). Unsupervised removal of systematic background noise from droplet-based single-cell experiments using CellBender. Nat. Methods *20*, 1323–1335.

67. Traag, V.A., Waltman, L., and van Eck, N.J. (2019). From Louvain to Leiden: guaranteeing well-connected communities. Sci. Rep. *9*, 5233.

68. Büttner, M., Ostner, J., Müller, C.L., Theis, F.J., and Schubert, B. (2021). scCODA is a Bayesian model for compositional single-cell data analysis. Nat. Commun. *12*, 6876.

69. Palla, G., Spitzer, H., Klein, M., Fischer, D., Schaar, A.C., Kuemmerle, L.B., Rybakov, S., Ibarra, I.L., Holmberg, O., Virshup, I., et al. (2022). Squidpy: a scalable framework for spatial omics analysis. Nat. Methods *19*, 171–178.

70. Cable, D.M., Murray, E., Zou, L.S., Goeva, A., Macosko, E.Z., Chen, F., and Irizarry, R.A. (2022). Robust decomposition of cell type mixtures in spatial transcriptomics. Nat. Biotechnol. *40*, 517–526.

71. Troulé, K., Petryszak, R., Prete, M., Cranley, J., Harasty, A., Tuong, Z.K., Teichmann, S.A., Garcia-Alonso, L., and Vento-Tormo, R. (2023). CellPhoneDB v5: inferring cell-cell communication from single-cell multiomics data. Preprint at arXiv.

72. Csárdi, G., Nepusz, T., Traag, V., Horvát, S., Zanini, F., Noom, D., and Müller, K. (2024). igraph: Network Analysis and Visualization in R. https://igraph.org.

73. Harrell, F. Hmisc: Harrell Miscellaneous (Github). https://doi.org/10.32614/CRAN.package.Hmisc.

74. La Manno, G., Soldatov, R., Zeisel, A., Braun, E., Hochgerner, H., Petukhov, V., Lidschreiber, K., Kastriti, M.E., Lönnerberg, P., Furlan, A., et al. (2018). RNA velocity of single cells. Nature *560*, 494–498.

75. Weiler, P., Lange, M., Klein, M., Pe'er, D., and Theis, F. (2024). CellRank 2: unified fate mapping in multiview single-cell data. Nat. Methods *21*, 1196–1205.

76. P. Badia-i-Mompel, D. Dimitrov, C.H. Holland, L. Garcia-Alonso, A. Valdeolivas, M. Ashtiani, A. Gabor (2020). dorothea (Bioconductor). https://doi.org/10.18129/B9.BIOC.DOROTHEA.

77. Browaeys, R., Saelens, W., and Saeys, Y. (2020). NicheNet: modeling intercellular communication by linking ligands to target genes. Nat. Methods *17*, 159–162.

78. Bray, N.L., Pimentel, H., Melsted, P., and Pachter, L. (2016). Near-optimal probabilistic RNA-seq quantification. Nat. Biotechnol. *34*, 525–527.

79. Badia-I-Mompel, P., Vélez Santiago, J., Braunger, J., Geiss, C., Dimitrov, D., Müller-Dott, S., Taus, P., Dugourd, A., Holland, C.H., Ramirez Flores, R.O., and Saez-Rodriguez, J. (2022). decoupleR: ensemble of computational methods to infer biological activities from omics data. Bioinform. Adv. *2*, vbac016.

80. Love, M.I., Huber, W., and Anders, S. (2014). Moderated estimation of fold change and dispersion for RNA-seq data with DESeq2. Genome Biol. *15*, 550.

81. Bankhead, P., Loughrey, M.B., Fernández, J.A., Dombrowski, Y., McArt, D.G., Dunne, P.D., McQuaid, S., Gray, R.T., Murray, L.J., Coleman, H.G., et al. (2017). QuPath: Open source software for digital pathology image analysis. Sci. Rep. *7*, 16878.

82. Slyper, M., Porter, C.B.M., Ashenberg, O., Waldman, J., Drokhlyansky, E., Wakiro, I., Smillie, C., Smith-Rosario, G., Wu, J., Dionne, D., et al. (2020). A single-cell and single-nucleus RNA-Seq toolbox for fresh and frozen human tumors. Nat. Med. *26*, 792–802.

83. Kong, L., Pokatayev, V., Lefkovith, A., Carter, G.T., Creasey, E.A., Krishna, C., Subramanian, S., Kochar, B., Ashenberg, O., Lau, H., et al. (2023). The landscape of immune dysregulation in Crohn's disease revealed through single-cell transcriptomic profiling in the ileum and colon. Immunity *56*, 2855.

84. Zheng, G.X.Y., Terry, J.M., Belgrader, P., Ryvkin, P., Bent, Z.W., Wilson, R., Ziraldo, S.B., Wheeler, T.D., McDermott, G.P., Zhu, J., et al. (2017). Massively parallel digital transcriptional profiling of single cells. Nat. Commun. *8*, 14049.

85. Korsunsky, I., Fan, J., Slowikowski, K., Zhang, F., Wei, K., Baglaenko, Y., Brenner, M., Loh, P.-R., and Raychaudhuri, S. (2018). Fast, sensitive, and accurate integration of single cell data with Harmony. Preprint at bioRxiv. https://doi.org/10.1101/461954.

86. Hao, Y., Stuart, T., Kowalski, M.H., Choudhary, S., Hoffman, P., Hartman, A., Srivastava, A., Molla, G., Madad, S., Fernandez-Granda, C., and Satija, R. (2024). Dictionary learning for integrative, multimodal and scalable single-cell analysis. Nat. Biotechnol. *42*, 293–304.

STAR★METHODS

KEY RESOURCES TABLE

REAGENT or RESOURCE	SOURCE	IDENTIFIER
**Antibodies**		
Mouse Anti HT2-280	Terrace Biotech	Cat # TB-27AHT2-280; RRID:AB_2832931
Donkey anti-Mouse IgG (H + L) Highly Cross-Adsorbed Secondary Antibody, Alexa Fluor™ Plus 647	Thermo Fisher Scientific	Cat # A32787; RRID:AB_2762830
Rabbit Anti-SFTPC	Millipore Sigma	Cat # ab3786; RRID:AB_91588
Donkey anti-Rabbit IgG (H + L) Highly Cross-Adsorbed Secondary Antibody, Alexa Fluor™ Plus 647	Thermo Fisher Scientific	Cat # A32795; RRID:AB_2762835
Alexa Fluor 488 goat anti-mouse IgG, IgM	Thermo Fisher Scientific	Cat # A10680; RRID:AB_2768739
Rat Anti-Mouse IgM MicroBeads	Miltenyi Biotec	Cat # 130-047-302; RRID:AB_244359
Mouse Anti-Cytokeratin 17	Santa Cruz	Cat # sc-393002; RRID:AB_2893006
Recombinant Alexa Fluor® 488 Rabbit Anti-Cytokeratin 5	Abcam	Cat # ab193894; RRID:AB_2893023
COL6A2 Polyclonal Antibody	Thermo Fisher Scientific	Cat # PA5-106556; RRID:AB_2854225
COL1A2 Monoclonal Antibody (4D1A7)	Thermo Fisher Scientific	Cat # MA5-38503; RRID:AB_2898416
Anti-alpha smooth muscle Actin antibody [1A4]	Abcam	Cat# ab7817; RRID:AB_262054
Goat anti-Rabbit IgG (H + L) Highly Cross-Adsorbed Secondary Antibody, Alexa Fluor™ 488	Thermo Fisher Scientific	Cat # A-11034; RRID:AB_2576217
Goat anti-Mouse IgG (H + L) Highly Cross-Adsorbed Secondary Antibody, Alexa Fluor™ 594	Thermo Fisher Scientific	Cat # A-11032; RRID:AB_2534091
**Biological samples**		
Non-ILD control lung tissue samples	MGH Lung Tissue Biorepository	Protocol # 2020P002765
IPF and other ILD lung tissue samples, including primary AT2 cultures	MGH Transplant Lung Biorepository	Protocol # 2013P002332
IPF and other ILD lung tissue samples	HSJ FIBRALUNG cohort study	Protocol # CES72-19, NCT05635032
Human bronchial epithelial cells	Endobronchial brush	Protocol # 2007P001050
**Chemicals, peptides, and recombinant proteins**		
Protector RNase inhibitor	Millipore Sigma	Cat # 3335402001
Cultrex Reduced Growth Factor Basement Membrane Extract, Type 2, Pathclear	R&D Systems	Cat # 3533-005-02
Gibco™ TrypLE™ Select Enzyme (1X), no phenol red	Fisher Scientific	Cat # 50-591-420
Maxima H Minus Reverse Transcriptase	Thermo Fisher Scientific	Cat # EP0752
KAPA HotStart HIFI 2 × ReadyMix	Roche Sequencing Store	Cat # 07958927001
Ampure XP beads	Beckman Coulter	Cat # A63881
Sodium citrate buffer pH 6.0	Thermo Fisher Scientific	Cat # 005000
Bovine Serum Albumin	Millipore Sigma	A9647-100G
PneumaCult™-Ex Plus Medium	STEMCELL Technologies	Cat # 05041
TRIzol™ Reagent	ThermoFisher Scientific	Cat # 15596026
TRIzol™ LS Reagent	ThermoFisher Scientific	Cat # 10296010
Fluoromount-G™ Slide Mounting Medium	Electron Microscopy Sciences	Cat # 17984-25
human TGF-β	Invivogen	Cat # rcyc-htgfb1
Lipofectamine™ RNAiMAX Transfection Reagent	ThermoFisher Scientific	Cat # 13778150
iTaq™ Universal SYBR® Green Supermix	Bio-Rad Laboratories	Cat # 1725124
Advanced DMEM/F12	ThermoFisher Scientific	Cat # 12634010
B-27 Supplement (50x), serum free	ThermoFisher Scientific	Cat # 17504044
N-2 Supplement (100x)	ThermoFisher Scientific	Cat # 17502001
HEPES (1 M)	ThermoFisher Scientific	Cat # 15630130

(*Continued on next page*)

Continued

REAGENT or RESOURCE	SOURCE	IDENTIFIER
Insulin-Transferrin-Selenium (ITS-G) (100x)	ThermoFisher Scientific	Cat # 41400045
GlutaMAX™ Supplement	ThermoFisher Scientific	Cat # 35050061
Antibiotic-Antimycotic (100X)	ThermoFisher Scientific	Cat # 15240062
SB431542	Tocris Bioscience	Cat # 1614-10
CHIR99021	Tocris Bioscience	Cat # 4423
BIRB796	Tocris Bioscience	Cat # 5989
Y27632	Tocris Bioscience	Cat # 1254
Heparin	StemCell Technologies	Cat # 07980
N-Acetyl Cysteine	Sigma-Aldrich	Cat # A9165
Human EGF Recombinant Protein	ThermoFisher Scientific	Cat # PHG0313
Recombinant human FGF10	Biolegend	Cat # 559304
Softwell 12 - Collagen coating - 0.5 or 50kPa hydrogel stiffness	Matrigen	Cat # SW12-COL-0.5-PK or SW12-COL-50-PK
ProLong™ Diamond Antifade Mountant with DAPI	ThermoFisher Scientific	Cat #P36962
TWEEN® 20	Sigma-Aldrich	Cat #P1379-500ML
Human TGF-beta 1 Recombinant Protein, PeproTech®	Thermo Fisher Scientific	Cat # 100-21
Recombinant Human Holo Lactoferrin	Prospec	Cat # PRO-592
Human IL-1 beta Recombinant Protein, PeproTech®	Thermo Fisher Scientific	Cat # 200-01B
Human IL-6 Recombinant Protein, PeproTech®	Thermo Fisher Scientific	Cat # 200-06
Recombinant Human IL-23 Protein	R&D Systems	Cat # 1290-IL-010
Recombinant Human IL-12/IL-23 p40 Monomer Protein	R&D Systems	Cat # 309-IL-010
Human/Mouse/Rat BMP-2 Recombinant Protein, PeproTech®	Thermo Fisher Scientific	Cat # 120-02
Human TRAIL (TNFSF10) (soluble) Recombinant Protein, PeproTech®	Thermo Fisher Scientific	Cat # 310-04
Human TNF-alpha Recombinant Protein, PeproTech®	Thermo Fisher Scientific	Cat # 300-01A
Human IFN-gamma Recombinant Protein, PeproTech®	Thermo Fisher Scientific	Cat # 300-02
Human TSLP Recombinant Protein, PeproTech®	Thermo Fisher Scientific	Cat # 300-62
Recombinant Human APP/Protease Nexin II Protein, CF	R&D Systems	Cat # 3466-PI-010
Recombinant Human u-Plasminogen Activator/Urokinase, CF	R&D Systems	Cat # 1310-SE
Human IGF-I Recombinant Protein, PeproTech®	Thermo Fisher Scientific	Cat # 100-11
Recombinant Human Osteopontin (OPN) Protein	R&D Systems	Cat # # 1433-OP-050
**Critical commercial assays**		
Chromium Next GEM Single Cell 3′ Kit v3.1	10× Genomics	Cat # PN-1000268, PN-1000120, PN-1000215
Dual Index Kit TT Set A	10× Genomics	Cat # 1000215
Qubit dsDNA HS Assay Kit	Thermo Fisher Scientific	Cat #Q32854
Agilent High Sensitivity DNA BioAnalyzer Kit	Agilent	Cat # 5067-4626
AllPrep DNA/RNA Mini Kit	Qiagen	Cat # 80204
Direct-zol RNA Microprep Kit	Zymo Research	Cat #R2062
Nextera XT DNA Library Preparation Kit	Illumina	Cat # FC-131-1096
NextSeq 1000/2000 P2 Reagents (100 cycles)	Illumina	Cat # 20046811
iScript™ cDNA Synthesis Kit	Bio-Rad Laboratories	Cat # 1708891

(*Continued on next page*)

Continued

REAGENT or RESOURCE	SOURCE	IDENTIFIER
**Deposited data**		
Previously published scRNA-seq dataset on pulmonary fibrosis	Reyfman et al.[11]	GEO accession #: GSE122960
Previously published scRNA-seq dataset on pulmonary fibrosis	Adams et al.[12]	GEO accession #: GSE136831
Previously published scRNA-seq dataset on pulmonary fibrosis	Habermann et al.[13]	GEO accession #: GSE135893
Previously published scRNA-seq dataset on pulmonary fibrosis	Carraro et al.[14]	GEO accession #: GSE143705
Previously published scRNA-seq dataset on pulmonary fibrosis	DePianto et al.[15]	GEO accession #: GSE159354
Previously published Visium data	Madissoon et al.[26]	ArrayExpress accession #: E-MTAB-11640
Previously published Visium data	Murthy et al.[28]	GEO accession #: GSE178361
snRNA-seq, spatial transcriptomics and bulk RNA-seq on AT2 cultures	This study	dbGAP accession #: phs004408.v1.p1<br>Broad Single Cell Portal #: SCP2879
snRNA-seq on HSJ samples	This study	SRA accession #: PRJNA1390708
**Experimental models: Cell lines**		
Human: Normal human lung fibroblasts (NHLF)	Lonza	Cat #: CC-2512
**Oligonucleotides**		
siRNA and qRT-PCR primers	Sigma-Aldrich	See Table S2
**Software and algorithms**		
Cell Ranger v5.0.1	10× Genomics	RRID:SCR_017344
Cumulus v2.1.0	Li et al.[65]	https://github.com/lilab-bcb/cumulus; RRID:SCR_021644
CellBender v0.2.0	Fleming et al.[66]	https://github.com/broadinstitute/CellBender; RRID:SCR_025990
Terra		https://app.terra.bio; RRID:SCR_021648
Leiden clustering algorithm	Traag et al.[67]	N/A
scCODA	Büttner et al.[68]	https://github.com/theislab/scCODA
squidpy	Palla et al.[69]	https://squidpy.readthedocs.io/en/stable/; RRID:SCR_026157
Robust cell type decomposition method	Cable et al.[70]	N/A
Loupe Browser	10× Genomics	RRID:SCR_018555
CellPhoneDB v5.0.0	Troulé et al.[71]	https://www.cellphonedb.org/; RRID:SCR_017054
igraph	Csárdi et al.[72]	RRID:SCR_019225
Harrell Miscellaneous (*hmisc)*	Harrell[73]	https://cran.r-project.org/package=Hmisc; RRID:SCR_022497
velocyto	La Manno et al.[74]	http://velocyto.org/; RRID:SCR_018167
scVelo	Weiler et al.[75]	https://github.com/theislab/scvelo; RRID:SCR_018168
DoRothEA	Badia-i-Mompel et al.[76]	https://www.bioconductor.org/packages/release/data/experiment/html/dorothea.html
NicheNet	Browaeys et al.[77]	https://github.com/saeyslab/nichenetr; RRID:SCR_023158
kallisto	Bray et al.[78]	https://pachterlab.github.io/kallisto/; RRID:SCR_016582
decoupleR	Badia-I-Mompel et al.[79]	https://saezlab.github.io/decoupleR/
DESeq2	Love et al.[80]	https://github.com/thelovelab/DESeq2
QuPath	Bankhead et al.[81]	https://qupath.github.io/; RRID:SCR_01825

EXPERIMENTAL MODEL AND STUDY PARTICIPANT DETAILS

For tissues obtained at Massachusetts General Hospital, subjects were either enrolled in the IRB-approved Lung Tissue Biorepository (protocol 2020P002765, used for all non-ILD controls) or the Transplant Lung Biorepository (protocol 2013P002332, used for all other samples including AT2 cultures). A sample of bronchial epithelial cells used for validating the keratin stainings was obtained from an endobronchial brush specimen of a subject without lung disease (collected under protocol 2007P001050). Potential 'transplant' subjects were identified through the MGH Lung Transplant Program. Potential 'resection surgery' subjects (i.e., non-transplant lung procedure) were identified through MGH Thoracic Surgery and Pulmonary Programs. Non-ILD controls were patients undergoing lung resection for a nodule without a history of ILD. ILD patients were included based on having a clinical diagnosis of IPF based on ATS criteria, or of another type of ILD. Patients were mailed study information and informed consent was obtained from all interested patients in accordance with the respective protocol. Sequencing, data storage, and publication plans were approved by the MGH IRB and the Office for Research Subject Protection at the Broad Institute.

Patients from the HSJ cohort were enrolled within the FIBRALUNG cohort study (protocol CES72-19, NCT05635032). Patients are generally recruited when undergoing an invasive diagnostic procedure, such as bronchoscopy with bronchoalveolar lavage fluid collection or lung biopsy. Transbronchial lung cryo- or forceps-biopsies were snap-frozen immediately after sampling and stored until use. All patients signed informed consent before enrollment in the study.

Tissues for spatial transcriptomics profiling were collected only at the MGH site from end-stage ILD patients and non-ILD Controls. Clinical information and metadata for the samples in this study are provided in Table S1.

The sample used for AT2 cultures was obtained from one female donor undergoing a lung transplant, with no history of smoking.

Sample sizes in this study are shown in Figures 1 and 2. Clinical and demographic information, as well as functional metadata for the samples in this study are provided in Table S1.

METHOD DETAILS

Nuclei isolation and snRNA-seq

Buffers

A 2× stock of ST buffer was prepared in ultrapure water with the following salts: 292 mM NaCl (Thermo Fisher Scientific, cat. no. AM9759), 20 mM Tris-HCl pH 7.5 (Thermo Fisher Scientific, cat. no. 15567027), 2 mM CaCl2 (VWR International Ltd, cat. no. 97062-820) and 42 mM MgCl2 (Sigma Aldrich, cat. no. M1028). This stock was used to prepare CST (1 mL of 2X ST buffer, 980 μL of 1% CHAPS (Millipore, cat. no. 220201), 10 μL of 2% BSA (New England BioLabs, cat. no. B9000S) and 10 μL of nuclease-free water) TST (1 mL of 2× ST buffer, 60 μL of 1% Tween 20 (Sigma Aldrich, cat. no. P-7949), 10 μL of 2% BSA (New England Biolabs, cat. no. B9000S) and 930 μL of nuclease-free water) and 1X ST (1mL 2X ST and 1mL ultrapure water).

Tissue dissociation

Tissues were dissociated either manually or automatically. For samples that were manually dissociated, tissues were chopped in TST buffer or CST buffer with Noyes Spring Scissors as previously described.[82] For samples that underwent automated dissociation, frozen tissue was placed into a gentleMACS C Tube (Miltenyi Biotec, cat. no. 130-093-237) containing 2mL of TST buffer with 1U/mL Protector RNase inhibitor (Millipore Sigma, cat. no. 3335402001). Tissue was dissociated by running the gentleMAC Dissociator (Miltenyi Biotec, cat. no. 130-096-427) "m_Spleen_01" program twice. The sample in the C tube was then incubated on ice for 5 min. C tubes were spun at 4°C for 2 min at 500 g. The pellet was resuspended in the TST buffer and then filtered through a 40μm Falcon cell strainer (VWR International, LLC, cat. no. 43-50040-51) into a 50 mL conical tube. The strainer was washed with 1mL 1XST buffer +0.5U/mL Protector RNase inhibitor. An additional 1mL of 1XST buffer+0.5U/mL Protector RNase inhibitor was used to wash the gentleMACS C Tube and then passed through the filter. A final filter wash was completed with 1mL of 1XST buffer +0.5U/mL Protector RNase inhibitor. The sample was transferred to a 15mL conical tube and centrifuged at 4°C for 10 min at 500 g. The pellet was resuspended in between 100 and 200 μl of 1XPBS (-Mg/-Ca)+ 1% BSA+1U/mL Protector RNase inhibitor buffer and filtered through a 35 μm Falcon cell strainer (Corning, cat. no. 352235).

Nuclei counting and encapsulation

Nuclei were counted using a INCYTO C-chip disposable hemocytometer (VWR International, cat. no. 22-600-100). 8,000–12,000 nuclei were loaded onto the Chromium Chips for the Chromium Next GEM Single Cell 3′ assay and libraries were constructed according to manufacturer instructions (Chromium Next GEM Single Cell 3′ Kit v3.1, cat. no. PN-1000268, PN-1000120, PN-1000215, 10× Genomics). Three libraries were prepared by pooling either three or two individual samples which were also processed individually.

Sample preparation for Visium profiling

Human lung tissue blocks were embedded in optimal cutting temperature (OCT) compound and flash frozen in −60°C isopentane. The OCT tissue blocks were sectioned in a cryostat (Leica, CM1950) at 10 μm tissue thickness at −22°C and placed on glass slides (VWR, Superfrost plus). Assessment of RNA quality was done by estimating RNA integrity numbers (RIN) from 100 μm tissue sections by RNA extraction using an AllPrep DNA/RNA Mini Kit (Qiagen, # 80204) and a 2100 Bioanalyzer (Agilent). Only samples with RIN scores >7 were considered for further processing. Hematoxylin and eosin (H&E) staining was performed to assess tissue morphology

and allow for pathology-guided selection of samples, and imaged using the Axio Imager.Z2 (ZEISS) light microscope at 10X magnification. 10 μm tissue sections were then placed on the Visium slides (10× Genomics) and processed according to the manufacturer's protocol. Briefly, the tissues were fixed in methanol and permeabilized for 18 min, a time point selected based on a tissue optimization experiment (10× Genomics, user guide CG000238). Libraries for sequencing were prepared with the Dual Index Kit TT Set A (cat. no. 1000215, 10× Genomics), pooled separately and sequenced on a NovaSeq 6000 (Illumina) with an SP or S1 flowcell.

Histopathology annotations

H&E-stained histologic sections of each tissue were used to manually identify histologic structures pertaining to salient anatomical and pathological regions. Using the Loupe Browser (10× Genomics), tissue regions were defined into one of the histopathological categories described below and the spots within those regions were selected for analysis. We ascertained the following anatomical regions: the alveolar parenchyma; airway bronchioles (Bronch) and vessels (Vasc); pleura; septum; and pathological structures such as fibroblastic foci, HC, muscle hyperplasia, lymphoid aggregates, and organizing pneumonia. Since each spot is 55 μm in size and spans regions that constitute divergent tissue structures, some spots may be situated at the interface of distinct regions.

Parenchyma from non-ILD controls was defined as Parenchyma-NIC. Parenchyma from ILD subjects was categorized as preserved zone (Parenchyma-P) with mildly thickened interstitial walls; fibrotic zone (Parenchyma-F) that appeared destructively fibrotic without visible parenchymal walls; transitional zone (Parenchyma-P/F TZ), located between the former two; or fibrotic inflamed zone (Parenchyma-FI), exhibiting marked chronic inflammation. We aimed to exclude spots that were present in large empty parenchymal airspaces. HC were categorized into the lumen space (HC-Lumen), epithelial lining (HC), and surrounding adventitial inflamed tissue (HC-Parenchyma FI). Within the pleura, we also identified regions of pleural parenchymal fibroelastosis in two non-ILD control tissues.

To more clearly associate histopathological communities with transcriptional profiles, we specifically did not label spots where the tissues were (1) folded on top of each other or (2) ambiguous in histological appearance/at the border of two distinct structures.

We inspected each tissue block via H&E staining, and observed a varying degree of the proportion of PP (Parenchyma-P) in these tissues. To account for these variations in our analysis, we categorized the ILD tissues with ≥ 50% of spots annotated as PP as ILD-PP^{high} and otherwise ILD-PP^{low} (Figure S4).

AT2 cell isolation

Excess lung tissue was obtained from surgical lung resections in accordance with MGH IRB (#2020P002765). Lung specimens were processed as previously described.[45] In brief, 1.5-3 g lung tissue was minced using a scalpel and digested using Collagenase, Dispase, and Dnase for 1 h at 37°C. The resulting single-cell suspension was filtered using a 100 μm strainer, washed with 10% FBS in DMEM/F12 and pelleted at 450 g for 10 min at 4°C. Cell pellets were resuspended in 1 mL MACS buffer (PBS containing 1% BSA and 2mM EDTA). Bead-based RBC depletion was performed (StemCell cat# 18170) using 10 μL of RBC depletion beads, followed by negative selection in a 5 mL tube in an EasyEight magnet. The remaining cells were filtered through a 40 μm strainer, pelleted, resuspended in MACS buffer, and incubated in TruStain FcX (BioLegend Cat.# 422302) block for 15 min at 4°C. Next, cells were washed and resuspended in MACS buffer, followed by incubation with HTII-280 mouse IgM antibody (Terrace Biotech Cat # TB-27AHT2-280, 1:60) for 1 h at 4°C while rocking. After washing, cells were incubated with secondary anti-mouse IgM-magnetic beads (1:10, Miltenyi Biotec Cat # 130-047-302) at 4°C for 30 min. The sample was washed two times with MACS buffer, then loaded onto a LS column (Miltenyi Biotec, 130-042-401). After 3 washes with MACS buffer, retained HTII-280 positive cells were eluted and plated for culture.

AT2 cell culture and cryopreservation

AT2 cells were resuspended at high concentration in a serum-free, feeder-free media (SFFF) prepared as described previously[45]: advanced DMEM/F-12 supplemented with the following (all final concentrations, 1x unless indicated otherwise: HEPES (15mM), Insulin-Transferrin-Selenium, GlutaMAX, Antibiotic-antimycotic, B27, N2, N-Acetyl-Cysteine (1.25mM), heparin (5μg/mL), hEGF (50ng/mL), hFGF10 (10ng/mL), hIL-1β (10ng/mL, only used for culture setup media), SB431542 (10μM), CHIR99021 (3μM), BIRB796 (1μM) and Y27632 (10μM). AT2 cells were then mixed with basement membrane extract (BME, R&D # 3533-005-02) to generate domes containing organoids. 2000–3000 AT2 cells in 5 μL of SFFF media were mixed with 45 μL of BME for each dome. The domes were seeded into 6-well plates such that individual wells contained 3–4 50 μL domes. The domes were cultured in SFFF media (2 mL/well, changed 3x per week) in a 37°C incubator under 5% CO2 atmosphere. Recombinant IL-1β (Thermo Fisher Scientific, #200-01B) was added to the media for the first 48–72 h for newly derived AT2 cell lines.[45]

AT2 cells were passaged similarly to previous descriptions.[45] After 14–16 days of growth, the organoids were digested using TrypLE Select (Fisher Scientific # 50-591-419) for 15 min. Subsequently, the digested organoids were sheared to a single cell suspension by pipetting through a non-filter 10 μL pipette tip, washed with PBS, and centrifuged at 450 g x 5 min at 4°C. The cell pellet was resuspended in SFFF and mixed with BME to form additional domes as described above. A small aliquot of cells from each passage was separated to assess for continued purity and cell quality by flow cytometry. These cells were fixed in 1% PFA for 20 min at room temperature and later stained for HTII-280 and anti-SPC according to the flow cytometry staining protocol detailed below.

Excess cells were cryopreserved by resuspension in CryoStor CS10 (Stem Cell Technologies #07930) at 1-2e6 cells/mL. The cells underwent slow freezing in a −80°C freezer and were transferred to liquid nitrogen storage the following day. Cryopreserved cells were recovered for culture by rapidly thawing in a 37°C water bath, diluted 1:10 in warmed DMEM/F12, centrifuged 450g x 5 min

at 4°C, and resuspended in SFFF media. To account for a reduction in colony-forming efficacy immediately after cryorecovery, 6000–7000 cells were plated per dome. Cells were cultured for one passage after cryorecovery under normal conditions with validation of cell quality by HTII-280/SPC staining as described before subsequent passages could be considered for experimental use.

AT2 organoids ligand screening

Cells were plated in BME domes at a concentration of 8000-10,000 cells per dome to account for the shorter endpoint compared to a normal passage. The cells were cultured for 7 days under standard condition, then stimulated with different ligands as described in Figures 4D–4F. The ligands used were as follows: TGF-β1 (Human TGF-beta 1 Recombinant Protein, PeproTech; Thermo Fisher Scientific, #100-21), LTF (Recombinant Human Holo Lactoferrin; Prospec, #PRO-592), IL-1β (Human IL-1 beta Recombinant Protein, PeproTech; Thermo Fisher Scientific, #200-01B), IL-6 (Human IL-6 Recombinant Protein, PeproTech; Thermo Fisher Scientific, #200-06), IL-23α (Recombinant Human IL-23 Protein; R&D Systems, #1290-IL-010), IL-12β (Recombinant Human IL-12/IL-23 p40 Monomer Protein; R&D Systems, #309-IL-010), BMP-2 (Human/Mouse/Rat BMP-2 Recombinant Protein, PeproTech; Thermo Fisher Scientific, #120-02), TNFSF10 (Human TRAIL (TNFSF10) (soluble) Recombinant Protein, PeproTech; Thermo Fisher Scientific, #310-04), TNF-α (Human TNF-alpha Recombinant Protein, PeproTech; Thermo Fisher Scientific, #300-01A), IFN-γ (Human IFN-gamma Recombinant Protein, PeproTech; Thermo Fisher Scientific, #300-02), TSLP (Human TSLP Recombinant Protein, PeproTech; Thermo Fisher Scientific, #300-62), APP (Recombinant Human APP/Protease Nexin II Protein, CF; R&D Systems, #3466-PI-010), PLAU (Recombinant Human u-Plasminogen Activator/Urokinase, CF; R&D Systems, #1310-SE), IGF-1 (Human IGF-I Recombinant Protein, PeproTech; Thermo Fisher Scientific, #100-11), and SPP1 (Recombinant Human Osteopontin (OPN) Protein; R&D Systems, #1433-OP-050). Subsequently, RNA isolation of organoids was performed after 7 days of stimulation. Domes containing organoids were lifted from the plate and dissolved in Trizol LS (Invitrogen, #10296010). Chloroform was added to induce phase separation and the aqueous phase was combined with ethanol and loaded with a Zymo RNA isolation kit (Zymo Research, #R2062) as per the manufacturer's instructions to purify each sample's total RNA.

Transcriptome sequencing using SMART-seq2

10 μg of RNA was added for reverse transcription with Maxima H Minus Reverse Transcriptase (EP0743, Thermo Fisher Scientific, MA, USA) and whole-transcription amplification (WTA) using KAPA HotStart HIFI 2 × ReadyMix (07958927001, Roche, Switzerland) for 13 cycles. The WTA products were then purified with Ampure XP beads (A63881, Beckman Coulter, CA, USA), quantified using the Qubit dsDNA HS Assay Kit (Q32854, Thermo Fisher, MA, USA), and analyzed with Agilent Bioanalyzer 2100 and High Sensitivity DNA BioAnalyzer Kit (5067-4626, Agilent, CA, USA). The diluted WTA products underwent tagmentation and indexing with the Nextera XT DNA Library Preparation Kit (FC-131-1096, Illumina, CA, USA), followed by size selection using Ampure XP beads. The size and concentration were then evaluated with the Agilent High Sensitivity DNA BioAnalyzer Kit. Libraries were pooled and sequenced using the Illumina NextSeq2000 P2 100cy kit (20046811, Illumina, CA, USA) and Illumina NextSeq2000 system. Raw FASTQ reads were processed using FastQC and pseudoaligned to the human reference transcriptome (GRCh38) using *kallisto*[78] to quantify gene abundances. Rounded counts were used for downstream analysis.

Detection of KRT17+KRT5- AT2 in organoids

Immunofluorescence

AT2 cells were plated and grown under normal conditions for the first seven days. They were then stimulated with ligand cocktail (TGF-β+TNF-α+ IL-1β) in SFFF media lacking TGF-β inhibitor SB431542 for 7 days. On day 14, domes containing organoids were lifted with the help of a scraper (Cell treat # 229306), embedded in a cryomold filled with OCT, and snap frozen. Subsequently, OCT-sectioned slides were processed for staining. In brief, the slides were thawed at 37°C for 1 h, washed with PBS 3 times, and fixed with 4% PFA at room temperature for 15 min. Antigen retrieval was performed using sodium citrate buffer pH 6.0 (Thermo Fisher Scientific # 005000) for 15 min at 95°C. Sections were permeabilized using PBST (0.1% Triton X-100 in PBS) for 15 min at room temperature followed by blocking in block-stain solution (1% BSA in PBST, Millipore Sigma, A9647-100G) for 1h at room temperature. Slides were incubated with primary mouse anti-KRT17 (diluted in block stain 1:100, Santa Cruz sc-393002) overnight at 4°C. Afterward, the slides were rinsed with PBST and then incubated with secondary antibody Donkey anti-Mouse AF647 (1:250, Thermo Fisher Scientific #A32787) and primary conjugated KRT5-AF488 (1:100, Abcam #AB193894) diluted in block-stain buffer for 2 h at room temperature. Hoechst (Thermo Fisher Scientific #H3570) was used for nuclear counterstaining for 5 min followed by washing and mounting with Fluoromount-G (Electron Microscopy Science # 17984-25). Image acquisition was performed using the Zeiss LSM780 confocal microscope at 63X.

Flow cytometry

AT2 cells were stimulated with the ligand cocktail as described above for confocal microscopy. On day 14, a single-cell suspension was generated as described above through digestion with TrypLE Select followed by mechanical shearing. Cells were fixed in 1% PFA for 20 min at room temperature. A sample of HBEC cells were fixed, stained, and analyzed alongside the AT2 cells using the same protocol to serve as a reference control for KRT5 and KRT17 staining. HBEC cells were generated from an endobronchial brush specimen and cultured in 2D on lab-made laminin-coated dishes in Pneumacult Ex-plus media (Stem Cell Technologies #05041). HBEC were detached with TrypLE Select and simultaneously fixed, stained, and analyzed with the above AT2 cells. Cells were permeabilized using 0.5% TWEEN 20 (Sigma-Aldrich #P1379-500ML) in PBS for 15 min at room temperature. Blocking was carried out

with 1% BSA in PBST for 1 h, at room temperature. A portion of the unstimulated AT2 cell sample was set aside to validate the purity of the input AT2 population. To evaluate KRT17 and KRT5 staining, the remaining portions of the samples were incubated with a mouse anti-KRT17 (Santa Cruz sc-393002, 1:100) primary antibody diluted in block-stain overnight at 4°C. Subsequently, the cells were washed, and the secondary antibody Donkey anti-Mouse AF-647 (Thermo Fisher Scientific #A32787, 1:250), and primary conjugated KRT5-AF488 (Abcam #AB193894, 1:100) were diluted in block-stain for 2 h at room temperature. Sample acquisition was performed using a CytoFLEX S flow cytometer (Beckman Coulter) and the results analyzed using Flowjo (version 10.10.0; BD Life Sciences).

To evaluate AT2 purity, cells were stained with HTII-280 (Terrace Biotech # TB-27AHT2-280, 1:100) and SPC (Millipore # ab3786, 1:200) primary antibodies diluted in block-stain overnight at 4°C. The cells were washed, followed by Alexa Fluor plus 647 donkey anti-rabbit IgG (Thermo Fisher Scientific # A32795) and Alexa Fluor 488 goat anti-mouse IgG, IgM (Thermo Fisher Scientific # A10680) conjugated secondary staining for two hours at room temperature diluted 1:250 in block stain. Flow cytometry acquisition and analysis was performed as described above.

Human lung fibroblasts culture

Primary human lung fibroblasts (HLFs) from IPF or healthy control donors were collected through the MGH Fibrosis Translational Research program from de-identified discarded excess tissue from clinically indicated surgical lung resections or lung transplant explants. Cells were grown in DMEM (Lonza) supplemented with 10% FBS (Lonza), 2 mM L-Glutamine (Lonza), 100 U/mL penicillin and 100 μg/mL streptomycin (Lonza) in a humidified incubator with 5% CO2 at 37°C. Unless otherwise stated, experiments were conducted using fibroblasts from a healthy control donor and IPF donor.

siRNA knockdown in primary lung fibroblasts

Arrayed siRNA screening of fibroblasts was performed as previously described.[83] Normal human lung fibroblasts (NHLF) were obtained from Lonza (CC-2512). Fibroblasts were maintained in DMEM containing GlutaMAX (Thermo Fisher, Catalog #10566016), supplemented with 10% (vol/vol) heat-inactivated FBS, NEAA (Gibco), penicillin/streptomycin (Corning). Cells were cultured at 37°C with 5% CO2.

Pre-designed pooled duplexes of siRNA oligomers were purchased from Sigma-Aldrich and re-suspended in nuclease-free water at 20μM. Sequences and catalog numbers are included in Table S2. Seeded NHLFs were transfected with 20 nmol siRNA complexed with Lipofectamine RNAiMAX (Thermo Fisher Scientific, #13778150) in Opti-MEM media (Thermo Fisher). 24 h later, cells were washed with PBS, then replenished with fresh media with or without the addition of 10 ng/mL of human TGF-β (Invivogen, #rcyc-htgfb1) for 24 h. Cells were washed in PBS, then resuspended in TRIzol reagent (Thermo Fisher Scientific, #15596026) for RNA isolation.

RNA isolation and quantitative RT-PCR

RNA was extracted from fibroblasts in TRIzol reagent following the manufacturer's protocol (Thermo Fisher). Equal amounts of RNA were used to synthesize cDNA with the iScript cDNA synthesis kit (Bio-Rad Laboratories, # 1708891). iTaq Universal SYBR Green Supermix (Bio-Rad Laboratories, # 1725124) was used for qRT-PCR on the C1000 Touch Thermal Cycler (Bio-Rad Laboratories). Gene expression was calculated with the ΔΔCt calculation with Hprt as the reference housekeeping gene. Oligos used for qRT-PCR can be found in Table S2.

Tissue culture plate stiffness assay

Healthy- or IPF-donor lung fibroblasts were perturbed with scrambled control siRNA or NFATC4-siRNA and seeded onto tissue culture plates coated either soft (0.5 kPa) or hard (50 kPa) hydrogel (Matrigen, SW12-COL-0.5-PK, SW12-COL-50-PK). Following 24 h of culture, with or without TGF-β stimulation (10 ng/mL), RNA was extracted from fibroblasts in TRIzol reagent followed by cDNA synthesis and qPCR as described in the siRNA experiments.

Immunofluorescence of pro-fibrotic markers

Cells were seeded on a coverglass (Celltreat, #229173) in a 12-well tissue-culture dish and after completion of experiment, cells were fixed in 2% PFA followed by three washes in PBS for five minutes each. Cells were permeabilized with 0.2% Triton X-100, then washed in PBS for five minutes each. Cells were blocked with 4% BSA-PBS and then incubated with 1:500 COL6A2 antibody (Thermo Fisher Scientific, PA5-106556), 1:200 COL1A2 antibody (Thermo Fisher Scientific, MA5-38503), or 1:1000 αSMA (Abcam, #ab7817) in 4% BSA-PBS for one hour at room temperature. Cells were washed with PBS and incubated with a 1:1000 dilution of AF-488 conjugated anti-rabbit antibody (Thermo Fisher Scientific, #A-11034) or a 1:1000 dilution of AF-594 anti-mouse antibody (Thermo Fisher Scientific, #A-11032) in 4% BSA-PBS for one hour. Cells were washed three times in PBS, rinsed in distilled water, and mounted with ProLong Diamond Antifade Mountant with DAPI (Thermo Fisher Scientific, #P36962) onto a glass slide for 24 h. Images were captured on a Nikon Ti2-E inverted microscope equipped with a CSU-W1 spinning disc confocal.

QuPath[81] imaging quantification software was used to quantify the fluorescence intensity of several collagen proteins or smooth-muscle actin. Nuclei were segmented and cell boundaries determined by the cell detection method, and cellular median fluorescence intensities of the different channels were quantified in each individual fibroblast.

QUANTIFICATION AND STATISTICAL ANALYSIS

snRNA-seq data pre-processing

Raw sequence files were demultiplexed using the *cellranger mkfastq* command (Cell Ranger[84] v5.0.1 10× Genomics) as implemented in the Docker image from Cumulus[65] (https://github.com/lilab-bcb/cumulus, version 2.1.0). Digital gene expression (DGE) matrices for each individual sample were obtained by aligning FASTQ sequence reads to the reference transcriptome. A customized reference of pre-mRNAs, including both introns and exons, was built from GRCh38 *cellranger* reference 1.2.0 and Ensembl v84 gene annotation following the recommendations provided by 10X's Cell Ranger pipeline. Cells with background or ambient RNA were removed by processing the raw DGE matrix through CellBender[66] (v0.2.0). CellBender was run on a Terra cloud computing environment (https://app.terra.bio) on all raw gene expression matrices using the *remove-background-v2-alpha* workflow with FPR = 0.01 option. Following ambient RNA correction, poor-quality cells or nuclei were identified and removed based on the following exclusion criteria: 1) cells or nuclei with <200 detected genes; 2) cells or nuclei having an outlier number of unique molecular identifiers (UMIs), i.e., >15,000; 3) cells or nuclei having an outlier number of identified genes, i.e., >5000; and 4) proportion of mitochondrial gene expression >2%

Demultiplexing of pooled samples

Genotype-based demultiplexing for the three pooled libraries was performed using *souporcell* (version 2021.03) as implemented in the Docker image from Cumulus. We also individually processed sample specific libraries for all pooled samples except one, which we utilized to demultiplex the pooled samples. First, for each pooled library, *souporcell* was run on *denovo* mode, i.e., without reference genotypes, by specifying the expected number of unique clusters to obtain cluster-specific genotypes. Unassigned cell barcodes or doublet barcodes were excluded from further analysis, Next, we also ran the individually processed samples using *souporcell* on *denovo* mode by specifying 1 as the number of expected clusters to obtain each individual sample-specific genotype. Then, using *bedtools intersect,* we overlapped the sample-specific genotypes with the demultiplexed cluster specific genotypes. Ultimately, clusters were assigned the sample identity with the maximum overlap of genotypes.

snRNA-seq atlas integration and annotation

Gene expression normalization was performed on the combined snRNA-seq dataset from all samples to account for differences in sequencing depth across cells. Count matrices of each individual cell were read-depth normalized using the logTP10K normalization procedure, i.e., number of transcripts per 10,000 transcripts in a cell. To minimize the effect of highly expressed genes, genes with >5% counts of the total count per cell were excluded from the computation of normalization factors. After natural logarithm conversion and scaling of the gene expression matrix, the top 2000 highly variable genes were selected for a first round of dimensionality reduction. PCA was conducted on the residual scaled expression matrix, and the top 60 eigenvectors were used to construct a k-nearest neighbor (k-NN) graph. Subsequently, the *leiden* clustering algorithm[67] was applied to cluster the cells and then visualized by the uniform manifold approximation and projection (UMAP) embedding algorithm. Based on the expression of known lineage markers of epithelial (*KRT8, EPCAM*), stromal (*PDGFRA, ACTA2, PDGFRB, RGS5, ITLN1*), endothelial (*PECAM1, PLVAP, VWF*) and immune populations (*CD79A, MZB1, CD3D, TRAC, C1QA, TPSAB, CSF3R*), the clusters were then sub-divided into the four major compartments for subsequent rounds of clustering and analysis.

In the epithelial compartment dataset, dimensionality reduction and batch correction was performed by adjusting for the following covariates: 10× Genomics Single Cell Gene Expression Solution chemistry (V1, V2 or V3); patient; study; dissociation protocol (TST or CST); and sample loading (pooled or single). The top 60 adjusted principal components were considered for neighborhood clustering using the *leiden* algorithm and visualized using UMAP embedding. Post-hoc analysis was performed on identified clusters to remove poor-quality cells, i.e., clusters with low UMI counts or high mitochondrial gene fraction, or expressing lineage markers of non-epithelial cells, were removed as doublet cells. Iterative rounds of clustering were performed until no doublet clusters were observed. The Wilcoxon rank-sum test was performed to define the markers specific to individual clusters and annotate each cluster. Markers used to annotate cell types in each compartment have been provided in Figures S1A–S1D.

Analyses for the stromal, endothelial and immune compartments followed a similar workflow as described above.

Generating the integratedILD scRNA-seq atlas

Previously published scRNA-seq datasets on pulmonary fibrosis[11–15] were downloaded from the Gene Expression Omnibus (GEO; GSE136831, GSE122960, GSE135893, GSE143705, GSE159354). Samples from chronic obstructive pulmonary disorder patients in Adams et al.[11–15] were removed from the meta-analysis. Altogether, the combined dataset consisted of 888,039 cells from 130 subjects after performing downstream quality control filtering. Data analysis was performed using the *scanpy* implementation. Poor-quality cells were removed based on the following exclusion criteria: 1) cells with <200 detected genes; 2) cells having an outlier number of UMIs, i.e., >25,000; 3) cells having an outlier number of identified genes, i.e., >6000; and 4) proportion of mitochondrial gene expression >35%. In general, the downstream analysis followed a similar workflow as described in the snRNA-seq atlas integration and annotation section above. Batch correction was performed by *harmony*[85] using the following covariates: Study + Chemistry + Patient. Further quality control was done post-hoc after iterative rounds of clustering to remove clusters with low

CellPress
OPEN ACCESS

UMI counts, high mitochondrial fraction, or expressing multi-lineage markers. Annotation of clusters was carried out by inspecting the expression of markers described in the earlier section. The final dataset consisted of 620,644 cells.

Mapping of snRNA-seq and scRNA-seq atlases

Cell type-specific genes (CSGs) were derived from the snRNA-seq atlas for each compartment using the FindAllMarkers function in Seurat.[86] The receiver operating characteristics (ROC) test was implemented, and all genes with area under the curve (AUC) values $\geq$0.65 were considered to be specific to a given cell type. The list of CSGs was then used to compute aggregated module scores in the scRNA-seq atlas for the matching compartment using the AddModuleScore function. Heatmaps of average module scores per cell type were visualized.

Cell composition analysis

To identify changes in the proportions of cell types between healthy and disease patients, we used the *scCODA*[68] package, which provides a Bayesian implementation of the Dirichlet-multinomial regression model. We combined the cell count data from both modalities, i.e., snRNA-seq and scRNA-seq atlases for differential abundance analysis, since we observed maximal overlap between the cell types labels in the two atlases (45 common cell types defined labels).

To combine the two datasets, we first noted that the compartment-level proportions in scRNA-seq data were vastly different from the snRNA-seq data (Figures S1E; S2J). For the scRNA-seq data, except for the Carraro et al.[11–15] which sorted only the epithelial cells, the protocol for sample processing in the other four studies was not clearly provided. Moreover, the snRNA-seq data were generated by processing only whole tissue fractions. Since sample processing can be a major factor influencing cell type composition, we addressed this using the following approach. To account for the uncertainty of tissue fractionation, we categorized each library from the remaining four studies in the scRNA-seq data into one of the following fractions: (1) epithelial enriched; (2) stromal enriched; (3) immune enriched; or (4) whole tissue, based on the total proportions of cell types from each compartment. A library was assigned to be epithelial enriched if the total fraction of epithelial cell types was >80%. Otherwise, if the total fraction of cells in the stromal or immune compartment was >60%, they were assigned to the respective fraction. If none of the above criteria were fulfilled, the library was assigned as a whole tissue fraction.

In the snRNA-seq data, we aggregated cell type count data at each patient level for each individual study. In the scRNA-seq data, we aggregated cell type count data at library level for each individual study, also keeping library-specific tissue fraction assignments from the earlier step. On the resulting combined cell count matrix, scCODA was implemented by adding a pseudocount of 0.1 and considering the following covariates: *modality* + *chemistry* + *study* + *fraction* + *disease status.* The relative abundances of each cell type in non-ILD controls and ILD subjects were visualized to identify a reference cell type whose abundance did not change between the two conditions. Finally, the scCODA model was run using the Arterial cells as the reference cell type, and cell types were considered to be significantly changing in abundance at FDR < 20%. Disease specific effect sizes and log fold change in abundances of only cell types with credible effects were visualized.

Community composition analysis

To identify changes in the proportions of communities between healthy and disease patients, we used the *scCODA* package. The patient-level community count matrix was first obtained, and the relative abundances of each community in non-ILD controls and ILD-PP^{high} and ILD-PP^{low} samples were visualized to identify a reference community whose abundance did not change between the two conditions. Finally, the scCODA model was implemented by adding a pseudocount of 0.1 and using the community c13 cells as the reference. Only communities that had credible effects at FDR < 20%) were considered to significantly change in abundance with reference to the Non-ILD Controls. Disease-specific effect sizes and log fold change in abundances of communities with credible effects were visualized.

Visium data pre-processing

Raw sequenced BCL files were demultiplexed using Space Ranger 2.1.1 (10$\times$ Genomics). Count matrices of gene expression were generated by aligning FASTQ reads to the GRCh38 (GENCODE v32/Ensembl 98) human reference transcriptome. Manual alignment to the fiducial frame and identification of tissue-covered spots were conducted using Loupe Browser (v.6, 10$\times$ Genomics). Spots corresponding to empty areas were excluded.

Visium data from published studies

Previously published Visium data of control lungs were integrated with data generated in the current study.[26,28] Madissoon et al.[26] profiled a total of 8 non-ILD control tissues from distal lung and was downloaded from ArrayExpress (accession number E-MTAB-11640). However, two tissues were deemed unsuitable for further analysis, as an inspection of the H&E stains revealed a bubble airspace. Murthy et al.[28] profiled distal lung tissue from one non-ILD control subject, and data were downloaded from GSE178361 at GEO.

Spatial transcriptomics analysis and community identification

Sample-specific gene expression matrices representing count of genes per spot (gene $\times$ spot) generated in the current study were combined with previously published Visium datasets and read depth-normalized using the logTP10K method. Quality control was

performed to remove poor-quality spots with UMI counts <500 or >35000 and/or mitochondrial gene fraction >20%. To minimize the effect of highly expressed genes, genes with >5% counts of the total count per cell were excluded from the computation of normalization factors. Subsequently, the top 2000 highly variable genes were identified on the natural logarithm transformed data. The normalized expression matrix was centered, the effect of total UMI count per cell was regressed out, and PCA was conducted to generate the top 100 pcs. Batch correction was then performed using the *harmony* package, considering the following variables as covariates: *Individual* + *Study*. After inspection of the elbow plot, the top 35 eigenvectors were used to construct a k-NN graph with *n_neighbors* = 20. Subsequently, the *leiden* clustering algorithm[67] was applied (*resolution* = 1.5) to cluster the spots and the resulting clusters were visualized by the uniform manifold approximation and projection (UMAP) embedding algorithm. Each cluster was considered to be a spatial domain of cellular communities (c0-c18).

Estimating cell count per Visium spot

Image processing was performed on the Visium H&E TIFF images using the *squidpy* package.[69] Each image underwent smoothing by Gaussian filtering, followed by nuclei segmentation using the *watershed* algorithm. Image features were then extracted and the number of unique segmentation objects (i.e., nuclei) under each spot were considered as the estimated number of cells per spot.

Integration of sc/snRNA-seq and spatial transcriptomics

Cell-type abundance estimates at each spot in the merged Visium data were obtained by implementing the robust cell type decomposition (RCTD) method[70] using either snRNA-seq atlas or scRNA-seq atlas as the reference. Raw count data were used as the reference and the RCTD was implemented in a *full mode* with default parameters, which infers abundance for all cell types in the reference atlas. For integration with scRNA-seq, the data were first downsampled to have a maximum of 7500 cells per unique cell type. The resulting weights per spot were normalized to sum to 1. Since a spot cannot contain every cell type, we devised a strategy to assign only the probable cell types to each spot. First, we inspected the distribution of the estimated number of cells per spot derived in the estimating cell count per Visium spot section, which averaged around 20. Using a threshold of >0.01 on the normalized weights, we observed that the number of unique cell types per spot approximated the number of cells/spot obtained from image processing. Therefore, for further analysis, normalized weights <0.01 for each spot were set to NaN.

We attributed more confidence to estimates based on scRNA-seq reference atlas for the following reasons: (1) the large dataset size of the scRNA-seq atlas in comparison to snRNA-seq, which allows more representative cells; (2) the inclusion of cytoplasmic mRNA in the scRNA-seq data as the cytoplasm generally accounts for a higher proportion of the cell volume than the nucleus, hence a higher fraction of the mRNA recovered by capture-based spatial transcriptomics.

Cell-type enrichment in spatial communities and histopathological groups

One-sided Student's *t* test was performed to identify cell types enriched in each of the spatial communities or histopathological groups. The RCTD-derived cell type abundance estimates at each spot, as described in the integration of sc/snRNA-seq and spatial transcriptomics section, were used. For each spatial community or histopathological group, abundances in all spots pertaining to that category were compared to the rest of the spots. Since the parenchyma was the most frequently annotated structure, to account for the imbalance in statistical comparisons, for each parenchymal histopathological group all spots pertaining to other parenchymal structures were removed from the background category. Only cell types with Benjamini-Hochberg adjusted *p*-value <0.05 were considered to be significantly enriched in a given community or histopathological group.

Cell communication network analysis

Intercellular crosstalk analysis was performed using the *cellphonedb (v5.0)* package.[71] The scRNA-seq atlas was used as the reference, and the DEG analysis method was implemented. First, the scRNA-seq dataset was subset to ILD and non-ILD controls and CSGs were computed using the Wilcoxon test separately for both conditions. Only statistically significant genes (*p*-value <0.05) and genes expressed in >10% of cells were considered for ligand-receptor analysis.

For inferring spatially constrained communication networks, condition-specific spatial neighborhoods of cell types were derived based on the RCTD-estimated cell type abundances at each spot, i.e., separately in ILD and non-ILD controls. One-sided Student's *t* test was performed to identify cell types enriched in each of the spatial communities identified in the spatial transcriptomics analysis and community identification section. Only cell types with Benjamini-Hochberg adjusted *p*-value <0.01 were considered to be significantly enriched in a given community. The list mapping each community to enriched cell types was supplied to *cellphonedb* for inference of cellular crosstalk.

The total number of significant L-R interactions were counted between a given *sender - receiver* pair of cell types and used to derive the adjacency matrix for network analysis. The *igraph* package[72] was used to construct a directed and weighted network graph on the adjacency matrix. Eigenvector centrality scores were computed to determine the influence of each node, i.e., cell type, in the condition-specific intercellular communication network graphs.

Co-localization analysis

We reasoned that the covariance between cell type abundance estimates at each Visium spot could be used to study their spatial neighborhoods. Co-localization estimates (CoLS) were obtained by performing Spearman's correlation between cell types

separately in non-ILD controls and ILD-PP^{low} tissues. ILD-PP^{high} tissues were not considered. Correlation analysis was performed using the *Hmisc* package[73] in R. Benjamini-Hochberg adjusted *p*-value <0.05 were considered statistically significant. Only cell type pairs with CoLS >0 were considered to be neighbors of each other.

Network analysis on CoLS estimates

CoLS estimates between cell types were obtained as described above and used to systematically construct separate network graphs of spatial proximity in non-ILD controls and diseased patients. To identify robust, high-confidence spatial neighborhoods, we used stringent thresholds to separate signal from noise. Only cell-type pairs with CoLS >0 and Benjamini-Hochberg adjusted *p*-value <0.01 were used for constructing the adjacency matrix of the network graph. The fast greedy algorithm was used to partition the network. Analysis was performed using the *igraph* package in R.

RNA velocity analysis

RNA velocity analysis of cells from the epithelial lineage was performed on a subset of 61,552 cells in the snRNA-seq atlas, for which the UMAP embeddings were recomputed. Count matrices for spliced and unspliced reads were generated for each 10x run using the *velocyto* software's[74] *run_10x* command mapping cell specific *bam* files to the *hg38* genome annotation file. The output *loom* files were merged and the proportion of spliced/unspliced reads for each cell subset were computed using the *scvelo* package[82]. Further filtering and normalization of the data were performed to estimate the RNA velocities using the dynamical model implementation. Finally, the velocity graph vector fields were visualized on top of the UMAP embedding. RNA velocity analysis of cells from the fibroblast lineage was performed on a subset of 11,151 cells from the scRNA-seq atlas and followed a similar pipeline as described above.

Identification of putative driver ligands of the Aberrant trAT gene program

First, we identified TFs highly enriched in Aberrant trAT cells based on their expression levels in either the snRNA-seq or the scRNA-seq atlas. Cluster-specific enrichment analysis was performed to identify genes enriched in Aberrant trAT compared to other alveolar epithelial cells: AT2, AT1, and trAT (only in snRNA-seq data). One-sided Wilcoxon rank-sum test was performed and TFs with adjusted *p*-value < 0.025 and mean expression log fold change $\geq$ 0.5 were considered to be enriched in Aberrant trAT cells, yielding a list of 38 putative TFs. Next, we identified TFs based on their inferred regulon activity using the *DoRothEA* package.[76] Enriched TFs were identified in Aberrant trAT cells using the one-sided Wilcoxon test by considering only the alveolar epithelial cells, AT2, AT1, and (only in RNA-seq data) trAT, as the background. We selected TFs with a mean activity difference of >0.5 and adjusted *p*-value < 0.025, yielding a list of 15 putative TFs. The above two lists were combined to yield a final list consisting of 51 unique TFs putatively driving the Aberrant trAT program.

Next, we utilized the ligand-target regulatory potential model available from *NicheNet*[77] to identify potential ligands whose signaling converges onto the enriched TFs. For each TF in our list, we selected the top 10 ligands based on the strongest regulatory potential scores derived from *NicheNet*. Only the ligands that appeared $\geq$ 2 times in the top 10 list were selected for further analysis. Subsequently, we restricted the list of ligands to those whose receptors were expressed in >5% of Aberrant trAT cells in both snRNA-seq and scRNA-seq data, ultimately yielding a total of 12 ligands. We further selected 3 ligands; *PLAU, IGF1* and *SPP1* based on the high enrichment of their receptors (*PLAUR*, *IGF1R* and *ITGAV/ITGB8*) in Aberrant trAT cells. Ultimately, this pipeline yielded 15 putative ligands driving the Aberrant trAT program.

Analysis of bulk RNA sequencing of AT2 organoids

Gene count matrix was processed using the *DESeq2*[80] package. Normalized expression was obtained by variance stabilization transformation, and top 500 genes were used for computing top 2 principal components. Pearson correlation was performed on normalized expression profiles followed by hierarchical clustering and visualized as a dendrogram.

Mapping of epithelial cell type specific signatures to bulk RNA transcriptome profiles was performed using *Seurat*. First, we derived epithelial cell type specific expression profiles from the scRNA-seq based atlas of the epithelial compartment, using the ROC curve test. Genes with AUC values $\geq$ 0.6 were considered part of the cell type specific expression signature. Lastly, we computed module scores for the CSG set in each sample using the *AddModuleScore* function and visualized the scores at group level.

Selection of MyoF.CTHRC1-hi specific TFs

TFs regulating the MyoF.CTHRC1-hi state were identified using two approaches. First, TF activity scores were inferred for each cell using the *decoupleR* package[79] in both snRNA-seq and scRNA-seq atlases. MyoF.CTHRC1-hi enriched TFs were identified using the Wilcoxon test by considering only all the cells in the stromal compartment as the background. We selected TFs with a mean activity difference of >0.5 and adjusted *p*-value < 0.01 that were statistically enriched in both snRNA-seq and scRNA-seq. Secondly, we inferred spot-level TF activity in Visium data and identified TFs enriched for activity in spatial community c8 using the criteria described above. Finally, 7 TFs (*TWIST1, TWIST2, RFX1, RUNX2, HES1, PIN1, NFATC4*) identified as enriched using both approaches were prioritized for siRNA screening. SMAD3 was used as a positive control.

Cell Genomics, Volume *6*

Supplemental information

Spatial transcriptomics reveals altered communities and drivers of aberrant epithelia and pro-fibrotic fibroblasts in interstitial lung diseases

Alok Jaiswal, Tristan Kooistra, Vladislav Pokatayev, Hélder N. Bastos, Rita F. Santos, Tresa R. Sarraf, Åsa Segerstolpe, Crystal Lin, Liat Amir-Zilberstein, Shaina Twardus, Kevin Shannon, Shane P. Murphy, Rachel Knipe, Ingo K. Ganzleben, Katharine E. Black, Toni M. Delorey, Daniel B. Graham, Yin P. Hung, Lida P. Hariri, Jacques Deguine, Agostinho Carvalho, Benjamin D. Medoff, and Ramnik J. Xavier

Supplemental Information

Spatial transcriptomics reveals altered communities and drivers of aberrant epithelia and pro-fibrotic fibroblasts in interstitial lung diseases

Alok Jaiswal,[1,2] Tristan Kooistra,[3,4] Vladislav Pokatayev,[1,2,5] Hélder N. Bastos,[6,7,8] Rita F. Santos,[7,9] Tresa R. Sarraf,[3,4] Asa Segerstolpe,[10] Crystal Lin,[10] Liat Amir-Zilberstein,[10] Shaina Twardus,[11] Kevin Shannon,[11] Shane P. Murphy,[10] Rachel Knipe,[3,4] Ingo K. Ganzleben,[3,4] Katharine E. Black,[3,4] Toni M. Delorey,[10] Daniel B. Graham,[1,2,5,10], Yin P. Hung,[12] Lida P. Hariri,[3,12] Jacques Deguine,[1,10] Agostinho Carvalho,[13,14] Benjamin D. Medoff,[3,4] Ramnik J. Xavier[1,2,5,10,15,16,17*]

[1]Broad Institute of MIT and Harvard, Cambridge, MA, 02142, USA
[2]Department of Molecular Biology, Massachusetts General Hospital, Boston, MA, 02114, USA
[3]Division of Pulmonary and Critical Care Medicine, Massachusetts General Hospital and Harvard Medical School, Boston, MA, 02114, USA
[4]Center for Immunology and Inflammatory Diseases, Massachusetts General Hospital and Harvard Medical School, Boston, MA, 02114, USA
[5]Center for Computational and Integrative Biology, Massachusetts General Hospital and Harvard Medical School, Boston, MA, 02114, USA
[6]Department of Pneumology, Hospital de São João, 4200-319 Porto, Portugal
[7]i3S – Instituto de Investigação e Inovação em Saúde, Universidade do Porto, 4200-135 Porto, Portugal
[8]Faculty of Medicine/RISE-Health, University of Porto, 4200-319 Porto, Portugal
[9]School of Health Sciences – Polytechnic of Porto, 4200-072 Porto, Portugal
[10]Klarman Cell Observatory, Broad Institute of MIT and Harvard, Cambridge, MA, 02142, USA
[11]Division of Gastroenterology, Massachusetts General Hospital and Harvard Medical School, Boston, MA, 02114, USA
[12]Department of Pathology, Massachusetts General Hospital and Harvard Medical School, Boston, MA, 02114, USA
[13]Life and Health Sciences Research Institute (ICVS), School of Medicine, University of Minho, 4710-057 Braga, Portugal
[14]ICVS/3B's - PT Government Associate Laboratory, 4710-057 Braga/4806-909 Guimarães, Portugal
[15]Center for the Study of Inflammatory Bowel Disease, Massachusetts General Hospital, Boston, MA, 02114, USA
[16]The Gene Lay Institute of Immunology and Inflammation, Brigham and Women's Hospital, Massachusetts General Hospital, Harvard Medical School, Boston, MA, 02115, USA
[17]Lead contact
*Correspondence: xavier@molbio.mgh.harvard.edu

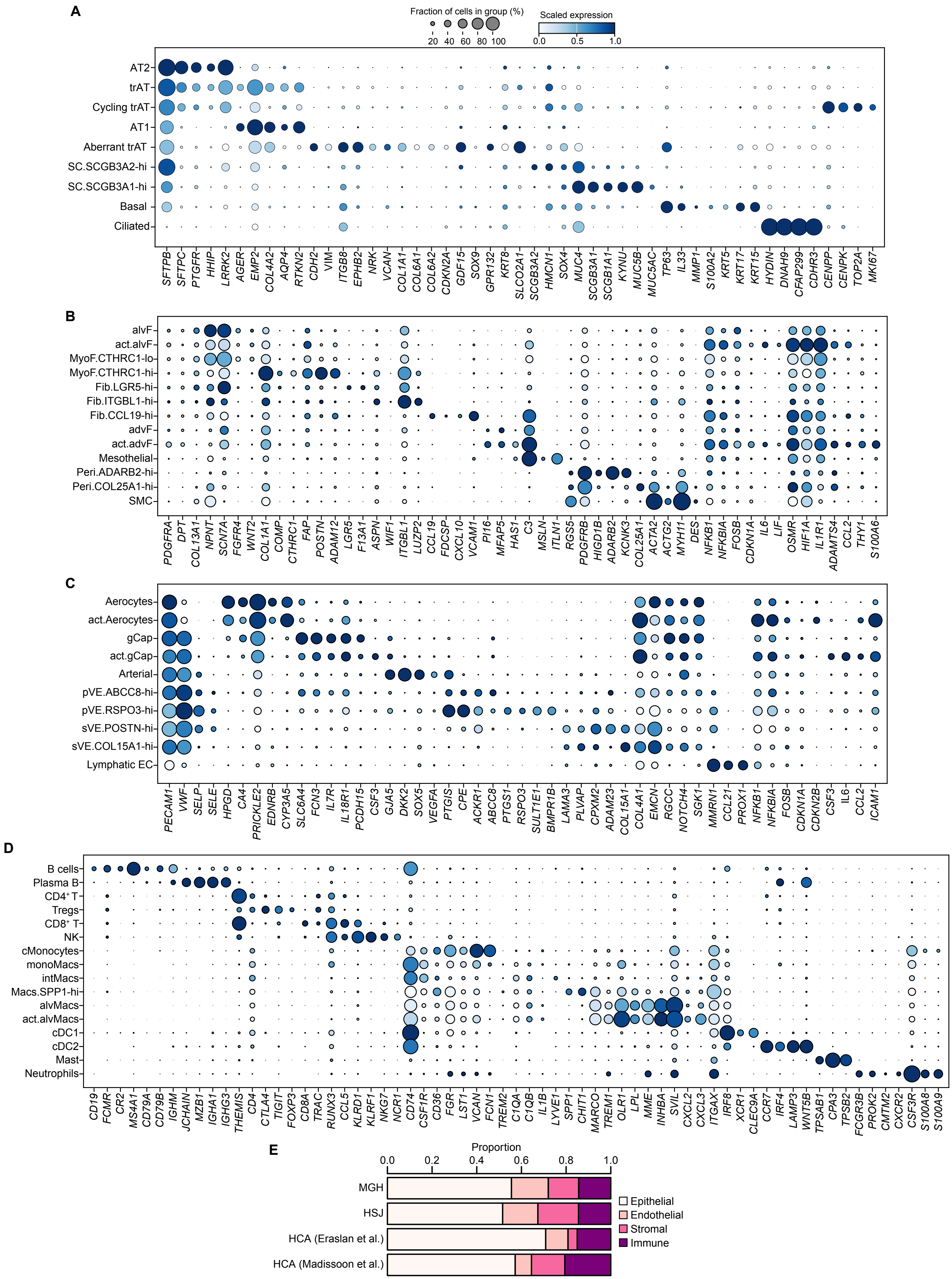
A
Fraction of cells in group (%)
20 40 60 80 100
Scaled expression
0.0 0.5 1.0
AT2
trAT
Cycling trAT
AT1
Aberrant trAT
SC.SCGB3A2-hi
SC.SCGB3A1-hi
Basal
Ciliated
SFTPB SFTPC PTGFR HHIP LRRK2 AGER EMP2 COL4A2 AQP4 RTKN2 CDH2 VIM ITGB8 EPHB2 NRK VCAN COL1A1 COL6A1 COL6A2 CDKN2A GDF15 SOX9 GPR132 KRT8 SLCO2A1 SCGB3A2 HMCN1 SOX4 MUC4 SCGB3A1 SCGB1A1 KYNU MUC5B MUC5AC TP63 IL33 MMP1 S100A2 KRT5 KRT17 KRT15 HYDIN DNAH9 CFAP299 CDHR3 CENPP CENPK TOP2A MKI67
B
alvF
act.alvF
MyoF.CTHRC1-lo
MyoF.CTHRC1-hi
Fib.LGR5-hi
Fib.ITGBL1-hi
Fib.CCL19-hi
advF
act.advF
Mesothelial
Peri.ADARB2-hi
Peri.COL25A1-hi
SMC
PDGFRA DPT COL13A1 NPNT SCN7A FGFR4 WNT2 COL1A1 COMP CTHRC1 FAP POSTN ADAM12 LGR5 F13A1 ASPN WIF1 ITGBL1 LUZP2 CCL19 FDCSP CXCL10 VCAM1 PI16 MFAP5 HAS1 C3 MSLN ITLN1 RGS5 PDGFRB HIGD1B ADARB2 KCNK3 COL25A1 ACTA2 ACTG2 MYH11 DES NFKB1 NFKBIA FOSB CDKN1A IL6 LIF OSMR HIF1A IL1R1 ADAMTS4 CCL2 THY1 S100A6
C
Aerocytes
act.Aerocytes
gCap
act.gCap
Arterial
pVE.ABCC8-hi
pVE.RSPO3-hi
sVE.POSTN-hi
sVE.COL15A1-hi
Lymphatic EC
PECAM1 VWF SELP SELE HPGD CA4 PRICKLE2 EDNRB CYP3A5 SLC6A4 FCN3 IL7R IL18R1 PCDH15 CSF3 GJA5 DKK2 SOX5 VEGFA PTGIS CPE ACKR1 ABCC8 PTGS1 RSPO3 SULT1E1 BMPR1B LAMA3 PLVAP CPXM2 ADAM23 COL15A1 COL4A1 EMCN RGCC NOTCH4 SGK1 MMRN1 CCL21 PROX1 NFKB1 NFKBIA FOSB CDKN1A CDKN2B CSF3 IL6 CCL2 ICAM1
D
B cells
Plasma B
CD4+ T
Tregs
CD8+ T
NK
cMonocytes
monoMacs
intMacs
Macs.SPP1-hi
alvMacs
act.alvMacs
cDC1
cDC2
Mast
Neutrophils
CD19 FCMR CR2 MS4A1 CD79A CD79B IGHM JCHAIN MZB1 IGHA1 IGHG3 THEMIS CD4 CTLA4 TIGIT FOXP3 CD8A TRAC RUNX3 CCL5 KLRD1 KLRF1 NKG7 NCR1 CD74 CSF1R CD36 FGR LST1 VCAN FCN1 TREM2 C1QA C1QB IL1B LYVE1 SPP1 CHIT1 MARCO TREM1 OLR1 LPL MME INHBA SVIL CXCL2 CXCL3 ITGAX IRF8 XCR1 CLEC9A CCR7 IRF4 LAMP3 WNT5B TPSAB1 CPA3 TPSB2 FCGR3B PROK2 CMTM2 CXCR2 CSF3R S100A8 S100A9
E
Proportion
0.0 0.2 0.4 0.6 0.8 1.0
MGH
HSJ
HCA (Eraslan et al.)
HCA (Madissoon et al.)
Epithelial
Endothelial
Stromal
Immune

Figure S1: Annotation of snRNA-seq atlas subsets, related to Figure 1.
A-D. Cell type-specific markers used to annotate snRNA-seq clusters among epithelial (A), stromal (B), endothelial (C), and immune (D) compartments. Dot size indicates fractions of cells expressing the gene and color indicates scaled expression values.
E. Proportions of cells in the epithelial, endothelial, stromal, and immune compartments in each cohort used to generate the snRNA-seq atlas.

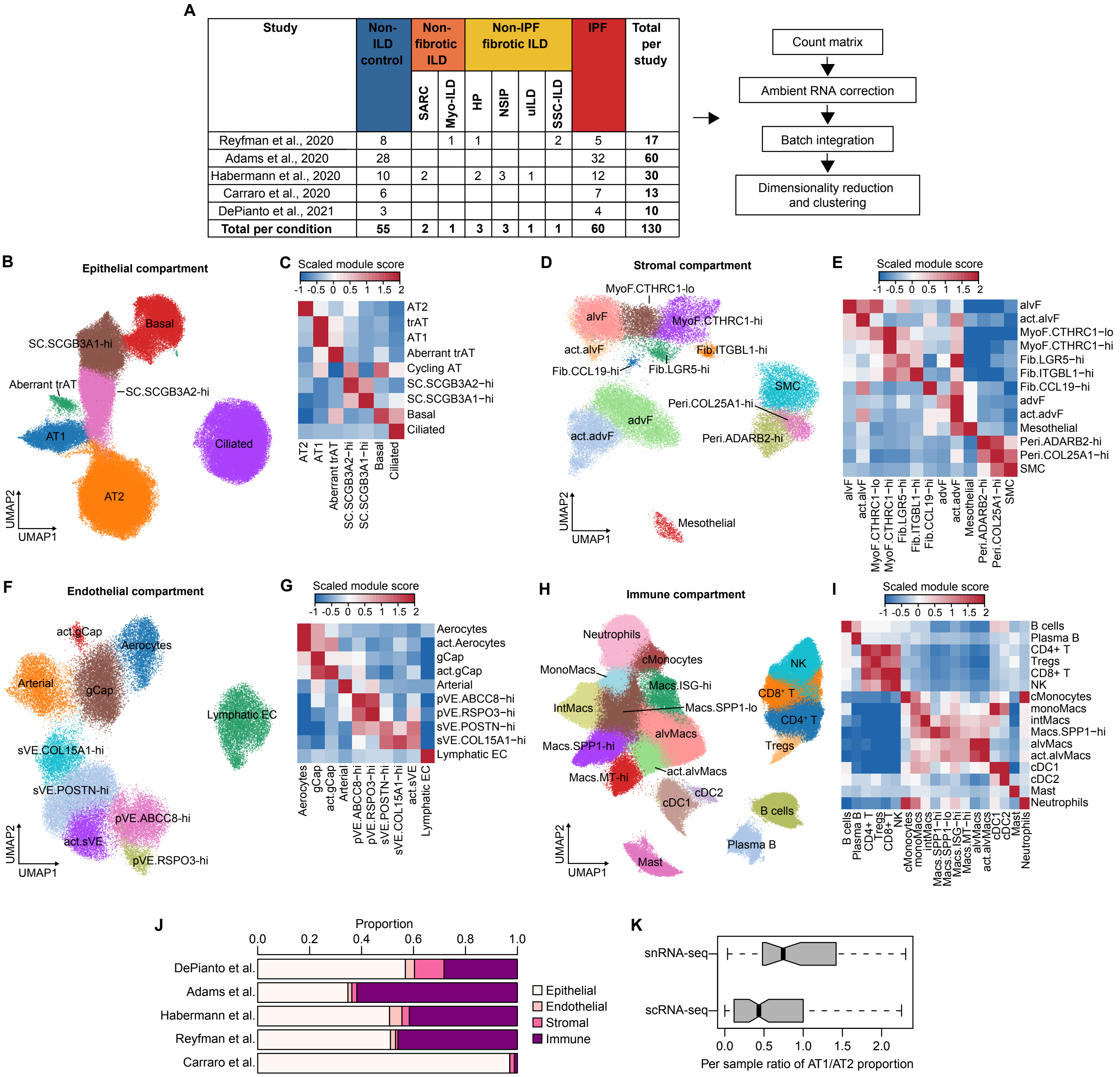

Study	Non-ILD control	Non-fibrotic ILD		Non-IPF fibrotic ILD				IPF	Total per study
		SARC	Myo-ILD	HP	NSIP	uILD	SSC-ILD		
Reyfman et al., 2020	8		1	1			2	5	**17**
Adams et al., 2020	28							32	**60**
Habermann et al., 2020	10	2		2	3	1		12	**30**
Carraro et al., 2020	6							7	**13**
DePianto et al., 2021	3							4	**10**
**Total per condition**	**55**	**2**	**1**	**3**	**3**	**1**	**1**	**60**	**130**

Figure S2: Assembly of a scRNA-seq atlas of ILD from published datasets, related to Figure 1.
A. Left: Sample sizes, by disease status, of published scRNA-seq datasets included in a meta-analysis for comparison with the snRNA-seq atlas. Right: Meta-analysis pipeline overview.
B-H. UMAP representation of the epithelial (B), stromal (D), endothelial (F) and immune (H) compartments of the joint scRNA-seq atlas, alongside the heatmap of module scores derived from the snRNA-seq atlas populations (y-axis) and calculated across the scRNA-seq subsets (x-axis) for the same epithelial (C), stromal (E), endothelial (G) and immune (I) compartments. **J.** Compartment proportions in the scRNA-seq data, separated by study.
K. Box plot of AT1/AT2 cell proportion per patient, showing that snRNA-seq has better recovery of AT1 cells compared to scRNA-seq. Bar plots represent the quartiles and whiskers represent the 10-90% range of values.

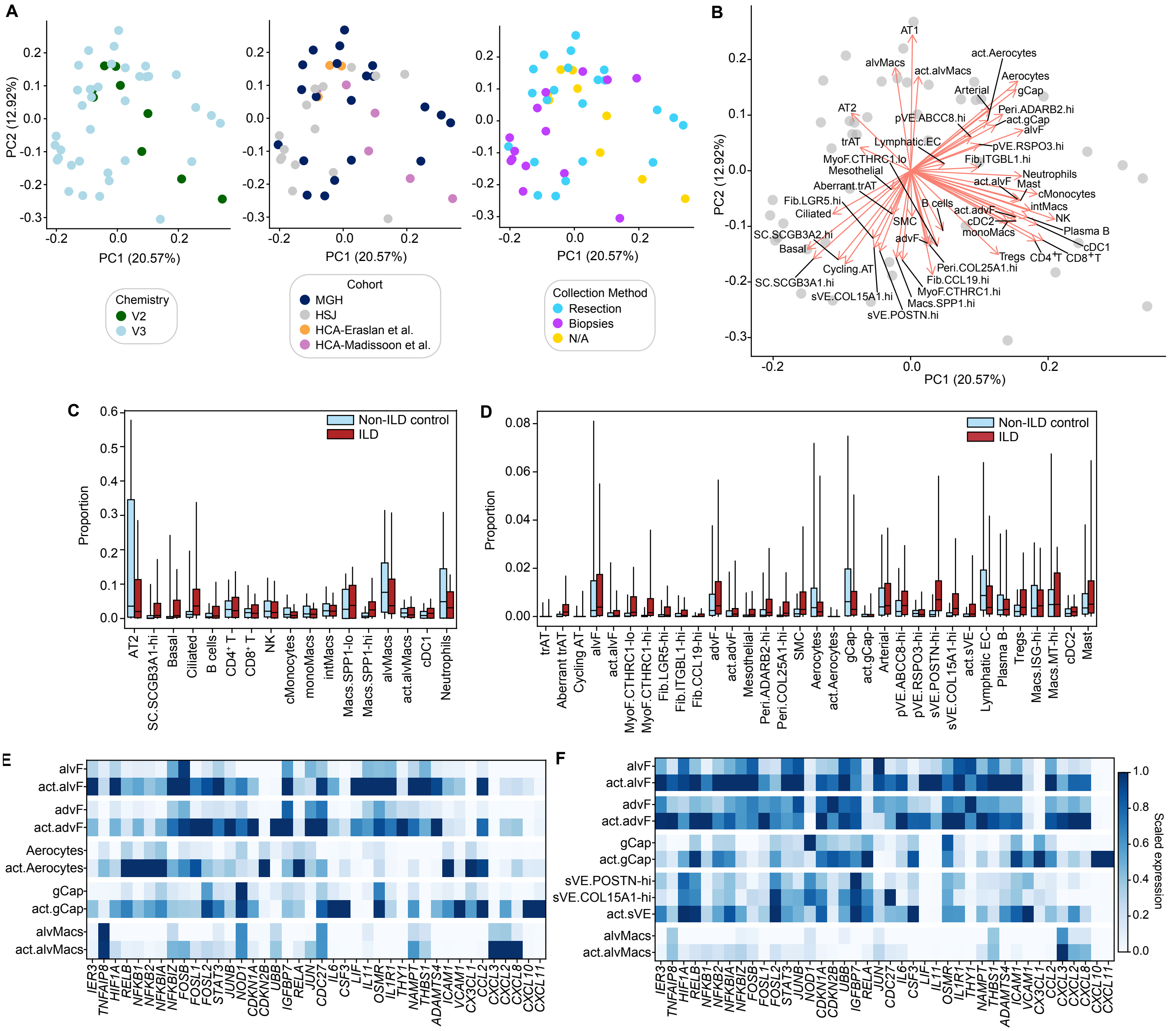
A
PC2 (12.92%)
PC1 (20.57%)
Chemistry
V2
V3
Cohort
MGH
HSJ
HCA-Eraslan et al.
HCA-Madissoon et al.
Collection Method
Resection
Biopsies
N/A
B
PC2 (12.92%)
PC1 (20.57%)
AT1
act.Aerocytes
alvMacs
act.alvMacs
Aerocytes
Arterial
gCap
AT2
pVE.ABCC8.hi
Peri.ADARB2.hi
act.gCap
alvF
trAT
Lymphatic.EC
pVE.RSPO3.hi
MyoF.CTHRC1.lo
Fib.ITGBL1.hi
Mesothelial
Neutrophils
act.alvF
Mast
Aberrant.trAT
cMonocytes
Fib.LGR5.hi
B cells
intMacs
Ciliated
act.advF
NK
SMC
cDC2
Plasma B
SC.SCGB3A2.hi
monoMacs
advF
cDC1
Basal
Tregs
CD4⁺T
CD8⁺T
Cycling.AT
Peri.COL25A1.hi
SC.SCGB3A1.hi
Fib.CCL19.hi
MyoF.CTHRC1.hi
sVE.COL15A1.hi
Macs.SPP1.hi
sVE.POSTN.hi
C
Proportion
Non-ILD control
ILD
AT2
SC.SCGB3A1-hi
Basal
Ciliated
B cells
CD4⁺ T
CD8⁺ T
NK
cMonocytes
monoMacs
intMacs
Macs.SPP1-lo
Macs.SPP1-hi
alvMacs
act.alvMacs
cDC1
Neutrophils
D
Proportion
Non-ILD control
ILD
trAT
Aberrant trAT
Cycling AT
alvF
act.alvF
MyoF.CTHRC1-lo
MyoF.CTHRC1-hi
Fib.LGR5-hi
Fib.ITGBL1-hi
Fib.CCL19-hi
advF
act.advF
Mesothelial
Peri.ADARB2-hi
Peri.COL25A1-hi
SMC
Aerocytes
act.Aerocytes
gCap
act.gCap
Arterial
pVE.ABCC8-hi
pVE.RSPO3-hi
sVE.POSTN-hi
sVE.COL15A1-hi
act.sVE
Lymphatic EC
Plasma B
Tregs
Macs.ISG-hi
Macs.MT-hi
cDC2
Mast
E
alvF
act.alvF
advF
act.advF
Aerocytes
act.Aerocytes
gCap
act.gCap
alvMacs
act.alvMacs
F
alvF
act.alvF
advF
act.advF
gCap
act.gCap
sVE.POSTN-hi
sVE.COL15A1-hi
act.sVE
alvMacs
act.alvMacs
Scaled expression
IER3
TNFAIP8
HIF1A
RELB
NFKB1
NFKB2
NFKBIA
NFKBIZ
FOSB
FOSL1
FOSL2
STAT3
JUNB
NOD1
CDKN1A
CDKN2B
UBB
IGFBP7
RELA
JUN
CDC27
IL6
CSF3
LIF
IL11
OSMR
IL1R1
THY1
NAMPT
THBS1
ADAMTS4
ICAM1
VCAM1
CX3CL1
CCL2
CXCL3
CXCL2
CXCL8
CXCL10
CXCL11

Figure S3: Compositional analyses of the snRNA-seq atlas, related to Figure 1.

A. Principal component analysis of annotated cell type composition colored by 10x chemistry, cohort, and tissue collection method (left to right).

B. Cell type loading weights on principal components. Arrow lengths are proportional to weights

C-D. Proportion changes of all cell types stratified by disease condition. Bar plots represent the quartiles and whiskers represent the 10-90% range of values.

E-F. Heatmaps of expression of selected genes specific to activated subsets and their respective basal cell states, in the (**E**) snRNA-seq and (**F**) scRNA-seq atlases. Genes that were differentially enriched in at least one activated subset were plotted. Differential expression analysis was performed using the Wilcoxon test in each broader cell type and genes with adjusted P-value < 0.05 were considered to be statistically significant.

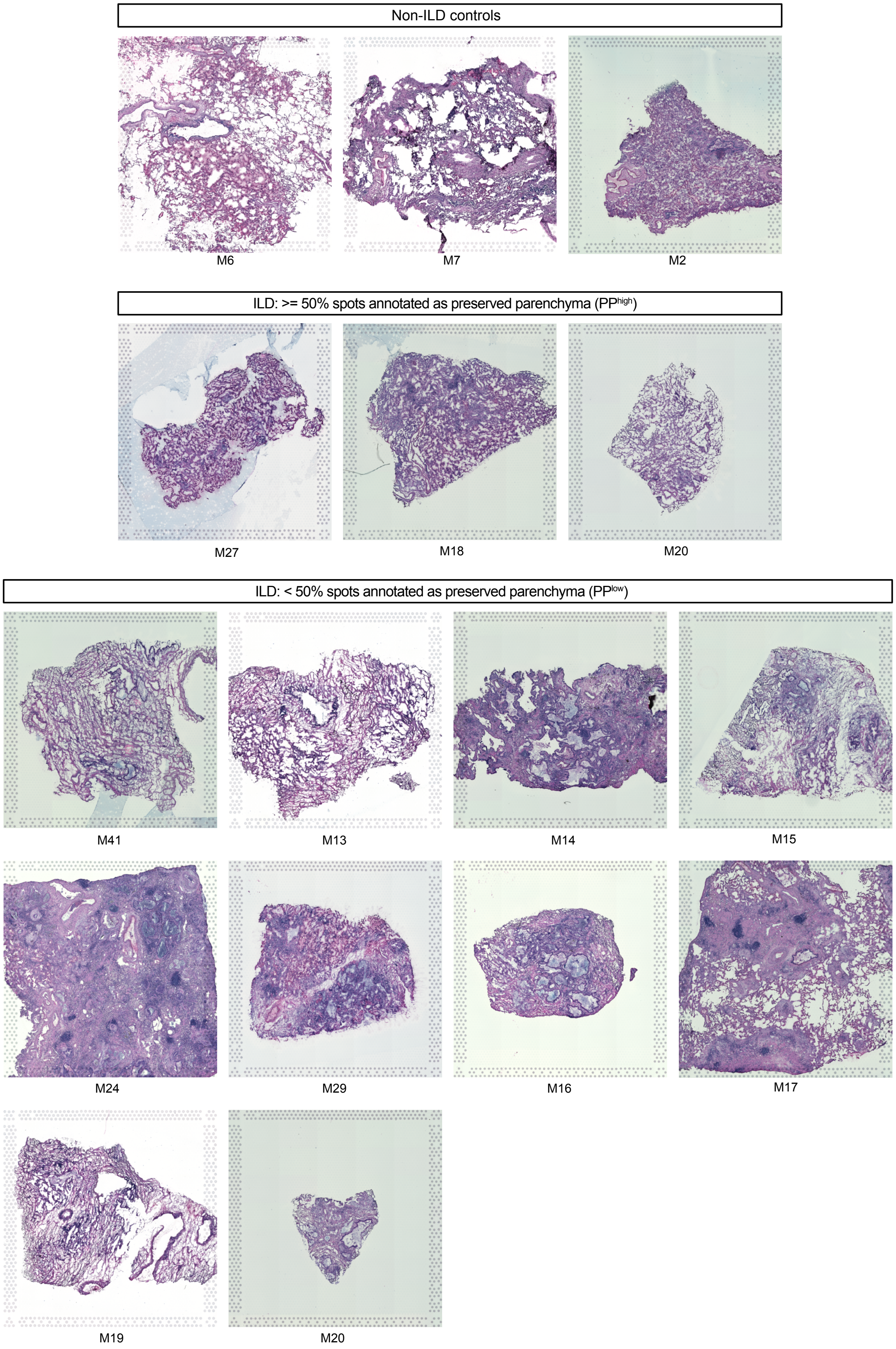
Non-ILD controls
M6
M7
M2
ILD: >= 50% spots annotated as preserved parenchyma (PPhigh)
M27
M18
M20
ILD: < 50% spots annotated as preserved parenchyma (PPlow)
M41
M13
M14
M15
M24
M29
M16
M17
M19
M20

Figure S4: Histological images of tissues analyzed by spatial transcriptomics, related to Figure 2. H&E stains of tissues profiled by 10x Visium. Each ILD tissue block was inspected and categorized as ILD-PP^{high} (with >= 50% of spots annotated as preserved parenchyma) or ILD-PP^{low} (with < 50% of spots annotated as preserved parenchyma).

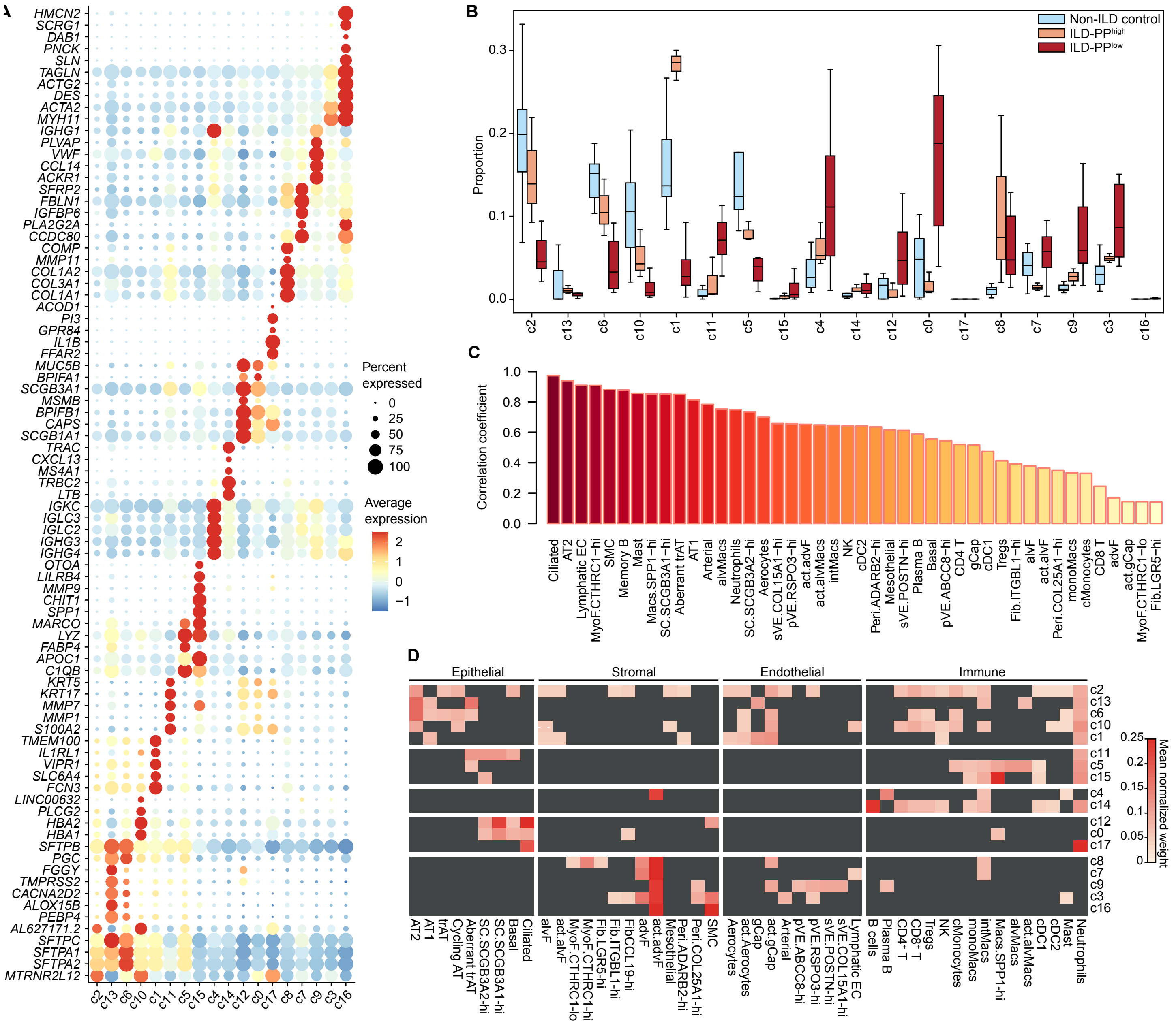
A
B
C
D
Percent expressed
Average expression
Non-ILD control
ILD-PPhigh
ILD-PPlow
Proportion
Correlation coefficient
Epithelial
Stromal
Endothelial
Immune
Mean normalized weight

Figure S5: Annotation of cellular communities detected by spatial transcriptomics, related to Figure 2.

A. Dot plot of expression for the top five genes specific to each cellular community identified in Figure 2B. Dot size represents the percentage of spots with at least 1 count of the gene, and color represents scaled expression values.

B. Proportion of all communities present in individual samples, grouped by disease condition and tissue histology. Bar plots represent the quartiles and whiskers represent the 10-90% range of values for individual samples.

C. Pearson correlation coefficients of RCTD-derived normalized cell type weights at each spot using either the scRNA-seq atlas or the snRNA-seq atlas as the reference.

D. Mean RCTD-estimated normalized weights of each cell type and cellular community using the snRNA-seq reference atlas. Cell type weights were statistically compared for each cellular community group to the weights of the rest using the one-sided Student's t-test. Legend color scale represents the mean normalized weight in the respective category for statistically significant (Benjamini-Hochberg adjusted P-value < 0.05) comparisons; insignificant comparisons are colored dark grey.

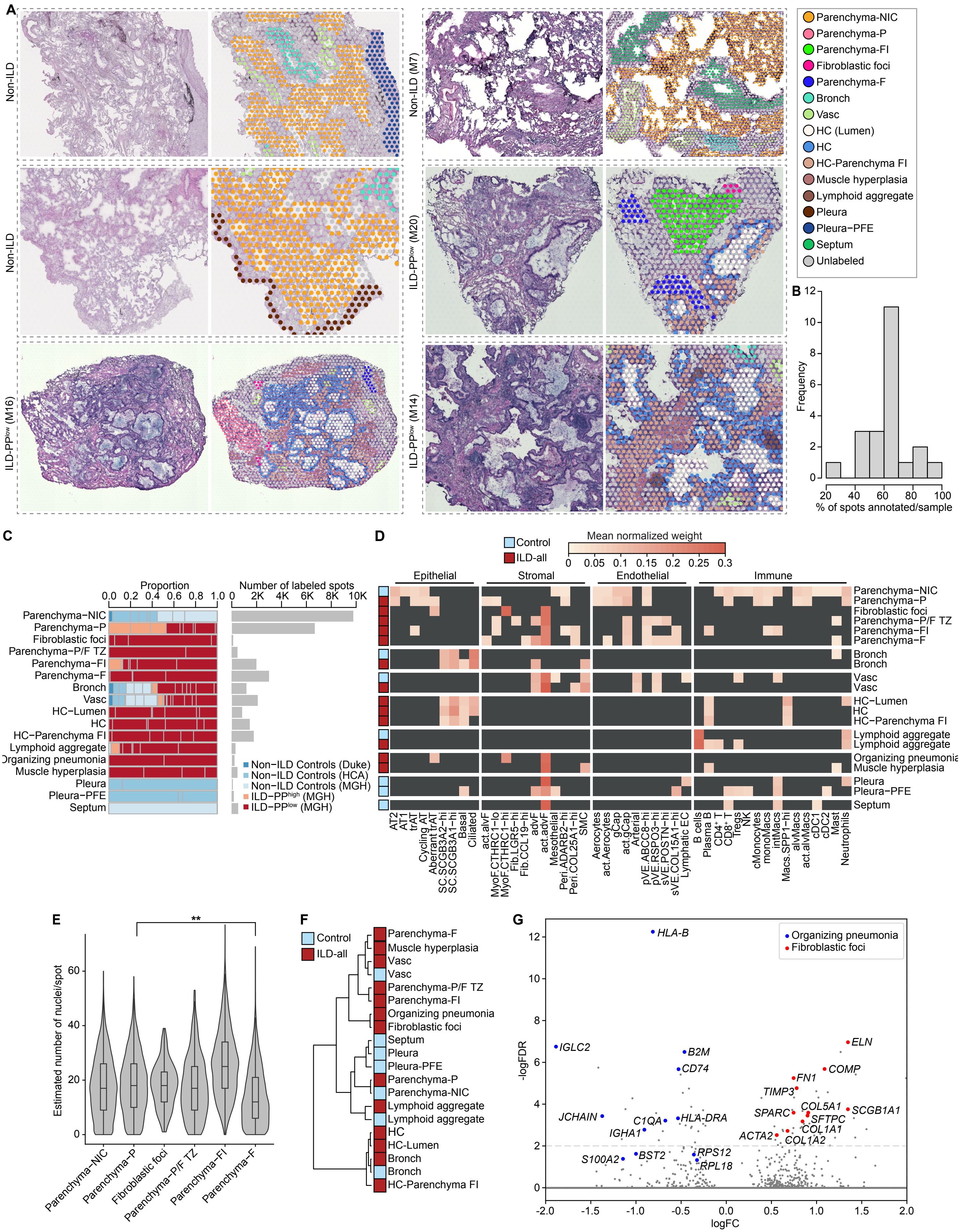
A
Non-ILD
Non-ILD
ILD-PP^low (M16)
Non-ILD (M7)
ILD-PP^low (M20)
ILD-PP^low (M14)
Parenchyma-NIC
Parenchyma-P
Parenchyma-FI
Fibroblastic foci
Parenchyma-F
Bronch
Vasc
HC (Lumen)
HC
HC-Parenchyma FI
Muscle hyperplasia
Lymphoid aggregate
Pleura
Pleura−PFE
Septum
Unlabeled
B
Frequency
% of spots annotated/sample
C
Proportion
Number of labeled spots
Parenchyma−NIC
Parenchyma−P
Fibroblastic foci
Parenchyma−P/F TZ
Parenchyma−FI
Parenchyma−F
Bronch
Vasc
HC−Lumen
HC
HC−Parenchyma FI
Lymphoid aggregate
Organizing pneumonia
Muscle hyperplasia
Pleura
Pleura−PFE
Septum
Non−ILD Controls (Duke)
Non−ILD Controls (HCA)
Non−ILD Controls (MGH)
ILD−PP^high (MGH)
ILD−PP^low (MGH)
D
Control
ILD-all
Mean normalized weight
Epithelial
Stromal
Endothelial
Immune
AT2
AT1
trAT
Cycling AT
Aberrant trAT
SC.SCGB3A2−hi
SC.SCGB3A1−hi
Basal
Ciliated
act.alvF
MyoF.CTHRC1−lo
MyoF.CTHRC1−hi
Fib.LGR5−hi
Fib.CCL19−hi
advF
act.advF
Mesothelial
Peri.ADARB2−hi
Peri.COL25A1−hi
SMC
Aerocytes
act.Aerocytes
gCap
act.gCap
Arterial
pVE.ABCC8−hi
pVE.RSPO3−hi
sVE.POSTN−hi
sVE.COL15A1−hi
Lymphatic EC
B cells
Plasma B
CD4+ T
CD8+ T
Tregs
NK
cMonocytes
monoMacs
intMacs
Macs.SPP1−hi
alvMacs
act.alvMacs
cDC1
cDC2
Mast
Neutrophils
E
Estimated number of nuclei/spot
**
Parenchyma-NIC
Parenchyma-P
Fibroblastic foci
Parenchyma-P/F TZ
Parenchyma-FI
Parenchyma-F
F
Control
ILD-all
Parenchyma-F
Muscle hyperplasia
Vasc
Vasc
Parenchyma-P/F TZ
Parenchyma-FI
Organizing pneumonia
Fibroblastic foci
Septum
Pleura
Pleura-PFE
Parenchyma-P
Parenchyma-NIC
Lymphoid aggregate
Lymphoid aggregate
HC
HC-Lumen
Bronch
Bronch
HC-Parenchyma FI
G
Organizing pneumonia
Fibroblastic foci
-logFDR
logFC
HLA-B
IGLC2
B2M
CD74
JCHAIN
C1QA
HLA-DRA
IGHA1
S100A2
BST2
RPS12
RPL18
ELN
COMP
FN1
TIMP3
SPARC
COL5A1
SCGB1A1
SFTPC
COL1A1
ACTA2
COL1A2

Figure S6: Analysis of disease-associated cellular communities, related to Figure 2.

A. Schema of identification of anatomical and histopathological features in representative H&E images for each sample.

B. Histogram of the number of samples, binned by fraction of annotated spots.

C. Left: Normalized proportion of sample level histopathological features colored by diseased status and cohort. Right: Number of annotated spots for each histopathological feature in the total dataset.

D. Mean RCTD-estimated normalized weights of each cell type and histopathological structure using the snRNA-seq reference atlas. Cell type weights were statistically compared for each group vs. the rest using the one-sided Student's t-test. Legend color scale represents the mean normalized weight in the respective category for statistically significant (Benjamini-Hochberg adjusted P-value < 0.05) comparisons; insignificant comparisons are colored dark gray.

E. Violin plot of estimated number of nuclei per spot in the annotated histopathological regions pertaining to the distal parenchyma. Nuclei segmentation was performed using the watershed algorithm. Nuclei counts per spot were statistically compared between Parenchyma-P and Parenchyma-F using the Wilcoxon test; **P < 0.001.

F. Dendrogram of hierarchical clustering of aggregate transcriptomic profiles of annotated histopathological features separated by patient groups. Dendrogram analysis was performed on the top 35 principal components.

G. Volcano plot showing differentially expressed genes between organizing pneumonia and fibroblastic foci. Wilcoxon test was performed and genes with adjusted P-value < 0.05 were considered statistically significant. Selected genes are highlighted.

For panels A, C-F: NIC - non-ILD control; P - preserved zone; F - fibrotic zone; TZ - transitional zone; FI - fibrotic inflamed zone; Bronch - bronchiole; Vasc - vasculature; HC - honeycomb cyst.

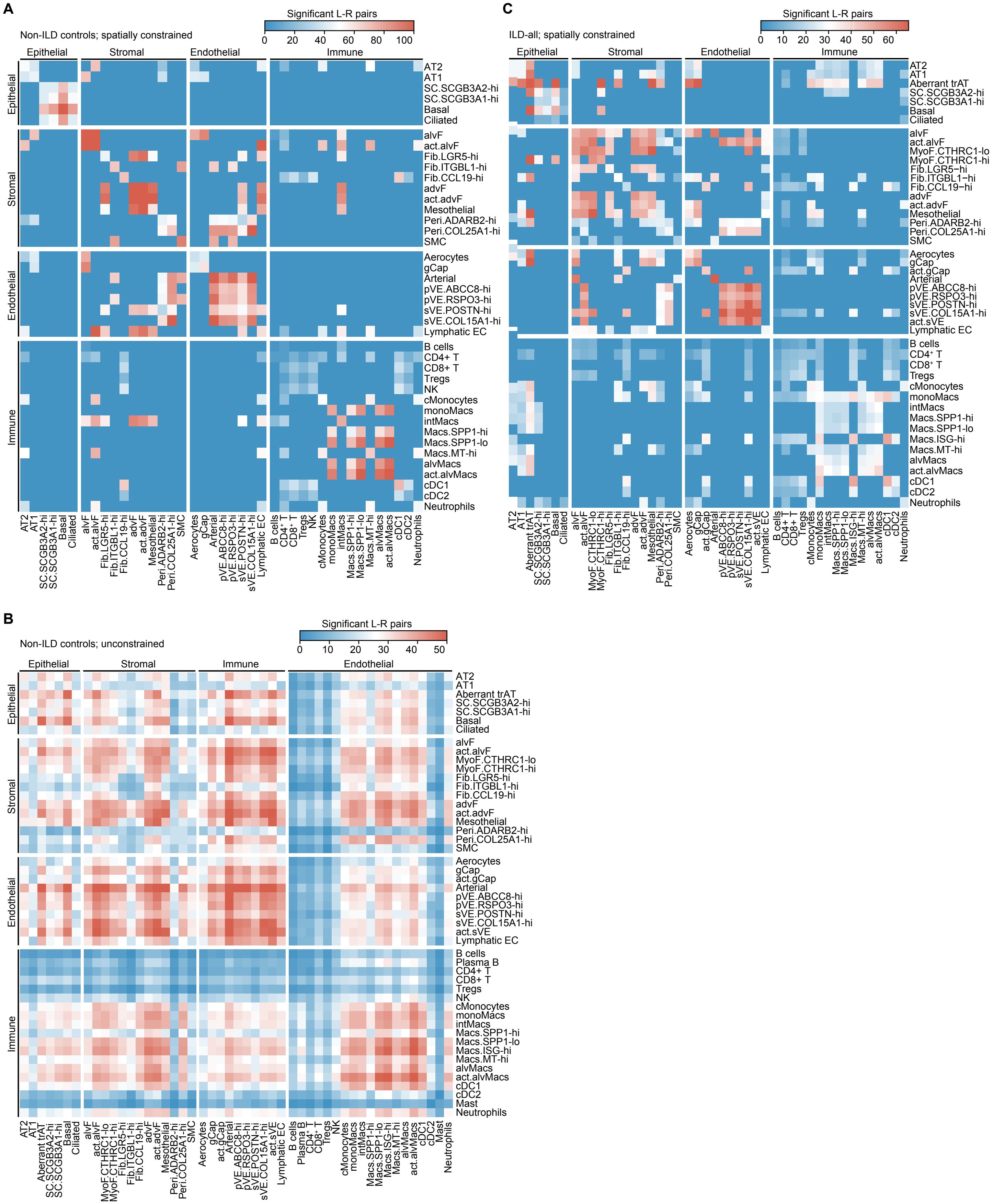
A
Non-ILD controls; spatially constrained
Significant L-R pairs
0 20 40 60 80 100
Epithelial
Stromal
Endothelial
Immune
AT2
AT1
SC.SCGB3A2-hi
SC.SCGB3A1-hi
Basal
Ciliated
alvF
act.alvF
Fib.LGR5-hi
Fib.ITGBL1-hi
Fib.CCL19-hi
advF
act.advF
Mesothelial
Peri.ADARB2-hi
Peri.COL25A1-hi
SMC
Aerocytes
gCap
Arterial
pVE.ABCC8-hi
pVE.RSPO3-hi
sVE.POSTN-hi
sVE.COL15A1-hi
Lymphatic EC
B cells
CD4+ T
CD8+ T
Tregs
NK
cMonocytes
monoMacs
intMacs
Macs.SPP1-hi
Macs.SPP1-lo
Macs.MT-hi
alvMacs
act.alvMacs
cDC1
cDC2
Neutrophils
C
ILD-all; spatially constrained
Significant L-R pairs
0 10 20 30 40 50 60
Epithelial
Stromal
Endothelial
Immune
AT2
AT1
Aberrant trAT
SC.SCGB3A2-hi
SC.SCGB3A1-hi
Basal
Ciliated
alvF
act.alvF
MyoF.CTHRC1-lo
MyoF.CTHRC1-hi
Fib.LGR5-hi
Fib.ITGBL1-hi
Fib.CCL19-hi
advF
act.advF
Mesothelial
Peri.ADARB2-hi
Peri.COL25A1-hi
SMC
Aerocytes
gCap
act.gCap
Arterial
pVE.ABCC8-hi
pVE.RSPO3-hi
sVE.POSTN-hi
sVE.COL15A1-hi
act.sVE
Lymphatic EC
B cells
CD4+ T
CD8+ T
Tregs
cMonocytes
monoMacs
intMacs
Macs.SPP1-hi
Macs.SPP1-lo
Macs.ISG-hi
Macs.MT-hi
alvMacs
act.alvMacs
cDC1
cDC2
Neutrophils
B
Non-ILD controls; unconstrained
Significant L-R pairs
0 10 20 30 40 50
Epithelial
Stromal
Immune
Endothelial
AT2
AT1
Aberrant trAT
SC.SCGB3A2-hi
SC.SCGB3A1-hi
Basal
Ciliated
alvF
act.alvF
MyoF.CTHRC1-lo
MyoF.CTHRC1-hi
Fib.LGR5-hi
Fib.ITGBL1-hi
Fib.CCL19-hi
advF
act.advF
Mesothelial
Peri.ADARB2-hi
Peri.COL25A1-hi
SMC
Aerocytes
gCap
act.gCap
Arterial
pVE.ABCC8-hi
pVE.RSPO3-hi
sVE.POSTN-hi
sVE.COL15A1-hi
act.sVE
Lymphatic EC
B cells
Plasma B
CD4+ T
CD8+ T
Tregs
NK
cMonocytes
monoMacs
intMacs
Macs.SPP1-hi
Macs.SPP1-lo
Macs.ISG-hi
Macs.MT-hi
alvMacs
act.alvMacs
cDC1
cDC2
Mast
Neutrophils

Figure S7: Cellular interaction analyses, related to Figure 3.

A. Heatmap of total sum of interaction scores between cell types, representing the landscape of intercellular signaling in scRNA-seq atlas profiles of non-ILD controls. Significant ligand-receptor interaction scores were computed using *cellphonedb* with constraints on spatial communities.

B. As in **A**, but scores were computed without constraints on spatial communities.

C. As in **A,** but using scRNA-seq atlas profiles of all ILD patients, with constraints on spatial communities.

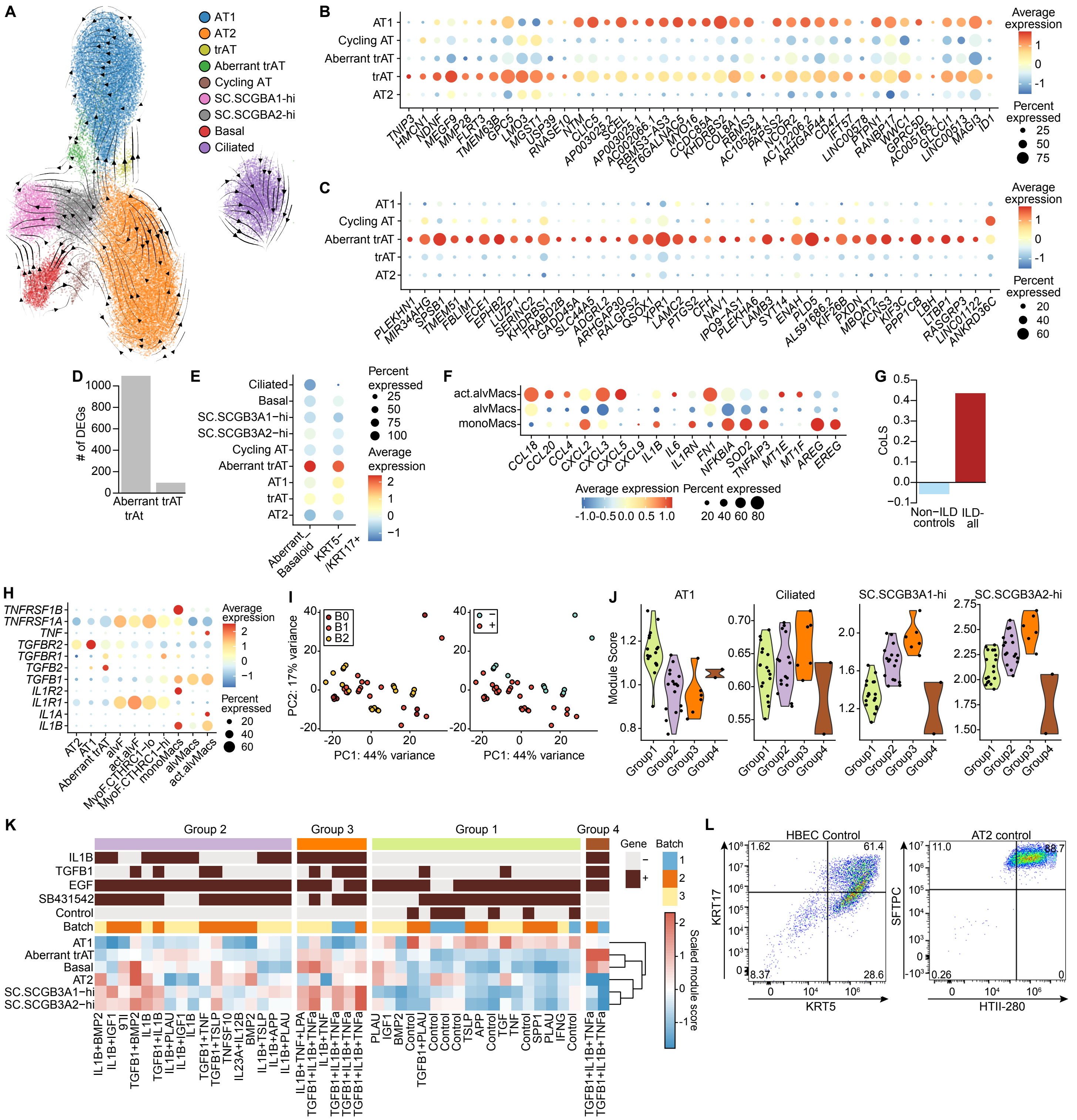

A
AT1
AT2
trAT
Aberrant trAT
Cycling AT
SC.SCGBA1-hi
SC.SCGBA2-hi
Basal
Ciliated
B
Average expression
Percent expressed
C
D
# of DEGs
Aberrant trAT
trAt
E
Aberrant_Basaloid
KRT5-/KRT17+
F
act.alvMacs
alvMacs
monoMacs
G
CoLS
Non-ILD controls
ILD-all
H
I
PC1: 44% variance
PC2: 17% variance
J
Module Score
Group1
Group2
Group3
Group4
K
Group 2
Group 3
Group 1
Group 4
Gene
Batch
Scaled module score
L
HBEC Control
AT2 control
KRT17
KRT5
SFTPC
HTII-280

Figure S8: Identification and validation of aberrant trAT cell programs, related to Figure 4.
A. UMAP of epithelial cells from snRNA-seq data embedded with RNA velocity vector fields, suggesting that Aberrant trAT and trAT cells differentiate from AT2 cells.
B-C. Dot plots of expression of selected (**B**) trAT-enriched and (**C**) Aberrant trAT-enriched markers.
D. Bar plot of number of differentially expressed genes enriched in Aberrant trAT and trAT cells.
E. Cell type signatures of previously published aberrant epithelial populations mapped to current snRNA-seq atlas.
F. Dot plots of expression of selected chemokines, cytokines and growth factors in selected cell types in the scRNA-seq data.
G. Bar plot of co-localization scores (CoLS) of AT1 and AT2 cells in non-ILD controls and all ILD patients.
H. Dot plots of expression of selected TGF-β, IL-1β and TNF-α family ligands and receptors in selected cell types in the scRNA-seq data.
I. Principal component analysis of transcriptional profiles in Figure 3F labeled by batch and presence (+) or absence (-) of TGF-β signaling inhibitor SB431542 in the culture media.
J. Violin plot of groupwise cell type-specific signature scores computed on transcriptome profiles using cell type-specific markers from the scRNA-seq atlas.
K. Heatmap of cell type-specific signatures for each stimulation.
L. FACS plots showing (left) validating gates for KRT5 and KRT17 in HBECs and (right) a strategy for isolating AT2 cells from human lung tissue by gating for SPTPC+ HTII280+ cells.

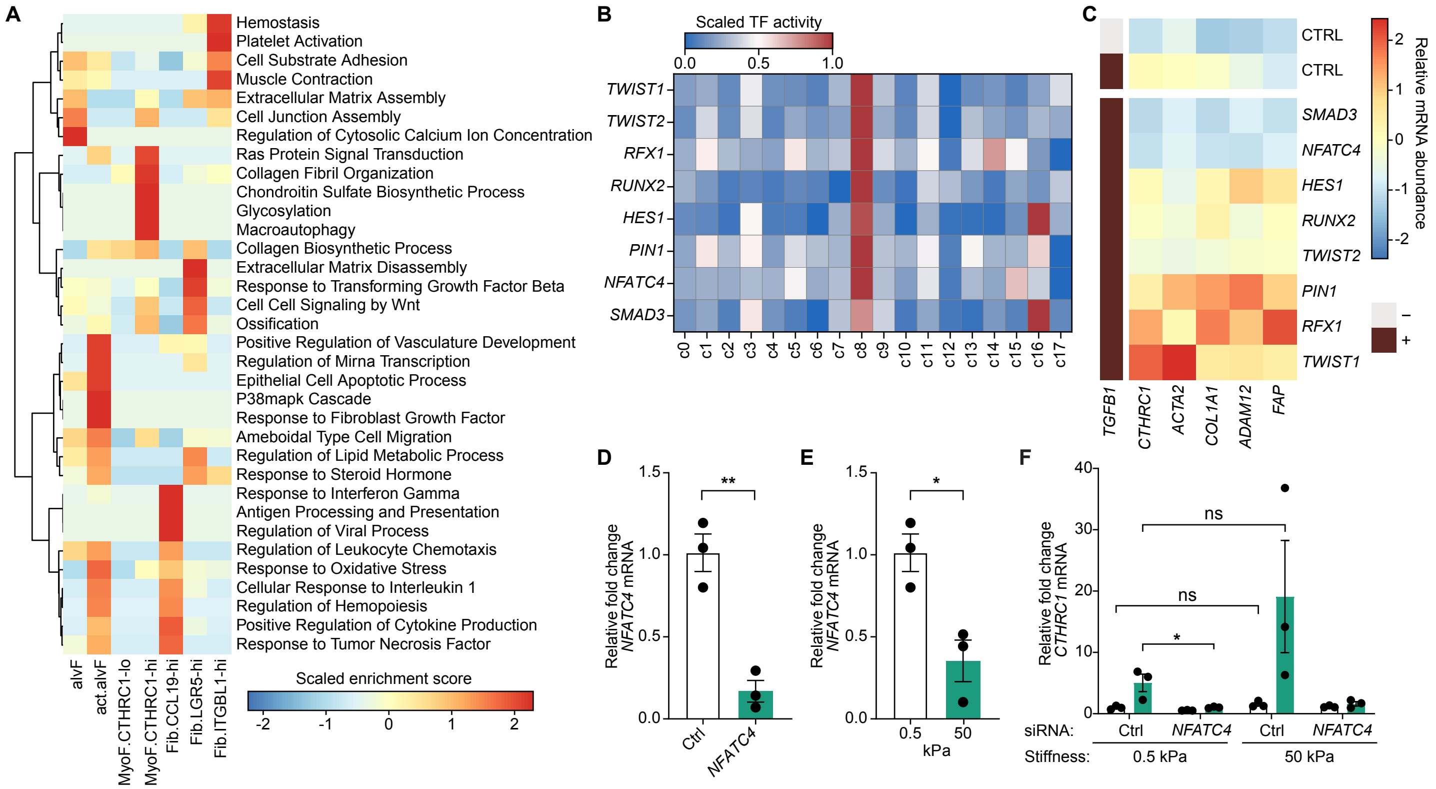
A
Hemostasis
Platelet Activation
Cell Substrate Adhesion
Muscle Contraction
Extracellular Matrix Assembly
Cell Junction Assembly
Regulation of Cytosolic Calcium Ion Concentration
Ras Protein Signal Transduction
Collagen Fibril Organization
Chondroitin Sulfate Biosynthetic Process
Glycosylation
Macroautophagy
Collagen Biosynthetic Process
Extracellular Matrix Disassembly
Response to Transforming Growth Factor Beta
Cell Cell Signaling by Wnt
Ossification
Positive Regulation of Vasculature Development
Regulation of Mirna Transcription
Epithelial Cell Apoptotic Process
P38mapk Cascade
Response to Fibroblast Growth Factor
Ameboidal Type Cell Migration
Regulation of Lipid Metabolic Process
Response to Steroid Hormone
Response to Interferon Gamma
Antigen Processing and Presentation
Regulation of Viral Process
Regulation of Leukocyte Chemotaxis
Response to Oxidative Stress
Cellular Response to Interleukin 1
Regulation of Hemopoiesis
Positive Regulation of Cytokine Production
Response to Tumor Necrosis Factor
alvF
act.alvF
MyoF.CTHRC1-lo
MyoF.CTHRC1-hi
Fib.CCL19-hi
Fib.LGR5-hi
Fib.ITGBL1-hi
Scaled enrichment score
-2
-1
0
1
2
B
Scaled TF activity
0.0
0.5
1.0
TWIST1
TWIST2
RFX1
RUNX2
HES1
PIN1
NFATC4
SMAD3
c0
c1
c2
c3
c4
c5
c6
c7
c8
c9
c10
c11
c12
c13
c14
c15
c16
c17
C
CTRL
CTRL
SMAD3
NFATC4
HES1
RUNX2
TWIST2
PIN1
RFX1
TWIST1
TGFB1
CTHRC1
ACTA2
COL1A1
ADAM12
FAP
Relative mRNA abundance
2
1
0
-1
-2
–
+
D
1.5
1.0
0.5
0.0
**
Relative fold change
NFATC4 mRNA
Ctrl
NFATC4
E
1.5
1.0
0.5
0.0
*
Relative fold change
NFATC4 mRNA
0.5
50
kPa
F
40
30
20
10
0
ns
ns
*
Relative fold change
CTHRC1 mRNA
siRNA:
Ctrl
NFATC4
Ctrl
NFATC4
Stiffness:
0.5 kPa
50 kPa

Figure S9: Regulation of fibroblast gene programs, related to Figure 5.
A. Heatmap of scaled enrichment scores of selected gene ontology (GO) pathways enriched in fibroblast cell types. Enrichment analysis was performed using *clusterprofiler* R package. Enrichment scores were derived by -log (P-values) and mean scaled.
B. Heatmap of selected transcription factors (TFs) enriched in estimated activity in cellular communities identified in the spatial transcriptomics data.
C. Heatmap of ECM-related genes detected by qPCR after siRNA-mediated knockdown of indicated TFs (X-axis).
D-E. Levels of *NFATC4* mRNA in healthy fibroblasts detected by qPCR (**D**) without (Ctrl) or with knockdown of *NFATC4* (**E**), stimulated with TGF-β, and cultured in soft (0.5 kPa) or rigid (50 kPa) substrates. $^*P < 0.05$, $^{**}P < 0.001$. Error bars represent SEM. Unpaired Student's t-test (two-sided). n = 3.
F. Relative levels of *CTHRC1* mRNA after TGFβ stimulation of IPF patient-derived Ctrl and *NFATC4*-knockdown fibroblasts cultured in soft (0.5 kPa) or rigid (50 kPa) substrates. $^*P < 0.05$. Error bars represent SEM. Unpaired Student's t-test (two-sided). n = 3.
